# Quantitative determination and validation of 96 pesticides in cannabis by LC-MS/MS and GC-MS/MS

**DOI:** 10.1007/s00216-025-05918-9

**Published:** 2025-06-11

**Authors:** D. A. MacKenzie, A. M. Anyanwu, G. McRae, J. E. Melanson

**Affiliations:** 1 National Research Council of Canada, Metrology, 1200 Montreal Road, Ottawa, ON K1A0R6 Canada; 2 Environment and Climate Change Canada, Air Quality Research Division, 335 River Road, Ottawa, ON K1V1C7 Canada

**Keywords:** Pesticides, Cannabis, LC-MS/MS, GC-MS/MS

## Abstract

Canada has established a strict set of testing requirements for pesticides that are unauthorized for use on cannabis, currently holding the most extensive list of analytes in North America to date, listing minimum method performance limits rather than maximum allowable concentration limits. These requirements establish the need for validated analytical methods capable of quantifying pesticides and growth regulators in highly variable cannabis inflorescence prior to distribution and sale. We have developed quantitative LC-MS/MS and GC-MS/MS methods capable of quantifying the 96 pesticides unauthorized in Canada for use on cannabis in dried cannabis flower and hemp. Herein, we report the validation results for linearity, precision, within- and between-sample accuracy, recovery, ion suppression, and limits of quantitation in dried cannabis inflorescence. Accuracy is evaluated in 10 cultivars varying in major cannabinoid content as well as ground whole-plant hemp to demonstrate method performance over a range of sample types. Results of the application of the current method to six cannabis samples seized from illegal storefronts by the Ontario Provincial Police are also reported.

**Graphical Abstract:**

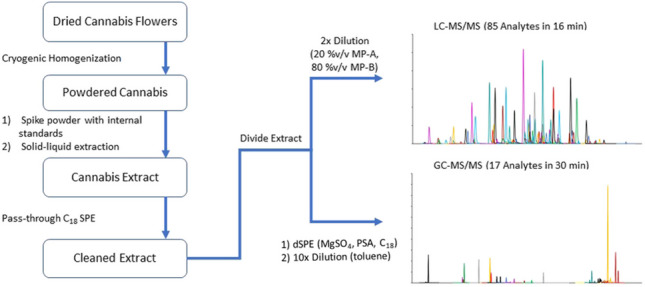

**Supplementary Information:**

The online version contains supplementary material available at 10.1007/s00216-025-05918-9.

## Introduction

The need for accurate evaluation of contaminants found in cannabis and cannabis products is becoming increasingly apparent as some jurisdictions around the world legalize cannabis for either medical or recreational usage. Canada is the second nation worldwide to establish a legal framework at the federal level for recreational cannabis with the intention of reducing harm to users through regulation and mandatory analytical testing [1]. This framework regulates all aspects of an emerging cannabis industry from cultivation to packaging and responsible distribution. While each province and territory has established its own system of distribution, the testing and reporting requirements remain unified nation-wide [2]. Testing requirements include the detection and quantitation of 96 pesticides listed in the Pesticide Active Ingredient List and Limits guide [3, 4]. Pesticide quantitation represents a notable technical challenge of this framework given the complexity of cannabis matrices and extensive list of analytes. The importance of pesticide testing, however, is underscored by a growing body of evidence of widespread pesticide use in unregulated cannabis production [5–10].

Illegal or unregulated cannabis samples in several countries have been found to contain a variety of pesticides and growth regulators that are known or suspected to be hazardous to humans upon consumption [5, 6, 11, 12]. Daminozide has a debated history of human carcinogenicity, myclobutanil has been shown to inhibit cholesterol synthesis in mammals and exacerbate fatty acid–induced steatosis, while imazalil can cause abnormal hormone production, as just a few examples [13–17]. Consumption of cannabis products contaminated with such substances poses a health risk to users and inhibits medicinal or therapeutic applications of cannabis products for those who may be immunocompromised or otherwise ill [11]. The World Health Organization classifies pesticides based primarily on their acute toxicity following oral ingestion or dermal contact; however, inhalation of smoke or vapor represents a common consumption method of cannabis products [18]. Limited research is available pertaining to the toxic effects of inhaled pesticides, particularly in human models, although spinosad has been shown to have deleterious effects on human lung A549 cells [19]. In rats, inhalation of cyfluthrin has been shown to reduce body weight and temperature, while cypermethrin can lead to reduced activity and reflexes in rats, as well as acute intoxication in dogs [20]. All cannabis products produced legally in Canada destined for the recreational market must be tested by a certified analytical testing lab as either the final formulation sold or as the batch of cannabis that was used to create the final product. Products not passing the rigorous testing standards are withheld from sale [21].

The challenge in pesticide quantitation in cannabis stems from three main obstacles: the complexity and diversity of the cannabis plant itself with strong interference caused by the abundant cannabinoids, the differing and extensive lists of analytes to be tested for in legalized jurisdictions, and the relatively low limits of quantitation (LoQ) required in many of these regulated markets. The plant itself has been found to contain up to 568 unique molecules as recently as 2017 including more than 113 cannabinoids which can comprise upwards of 250 mg/g (25% w/w) of dried inflorescence while varying widely in abundance, particularly of the major naturally occurring cannabinoids Δ9-tetrahydrocannabinolic acid (THCA) and cannabidiolic acid (CBDA), and their associated neutral forms, tetrahydrocannabinol (THC) and cannabidiol (CBD) [22–27]. These components have the potential to be co-extracted along with the target analytes of a given assay, complicating analysis in terms of chromatographic separation, direct interference in the case of optical detection methods, or ion suppression in mass spectrometry applications. The list of required analytes in Canada is extensive and broad, encompassing residues that are poorly suited to traditional LC-MS/MS techniques, and a wide range in polarity of analytes with daminozide and acequinocyl representing the extremities. In addition to the diverse list of analytes is the method performance requirements which stipulate maximum lower limits of quantitation ranging from 10 to 3000 ng/g (ppb) for pesticide and plant growth regulators in dried inflorescence. Extraction and detection of such a structurally diverse list of analytes in the face of hundreds of potentially co-extracted components present in up to 7 orders of magnitude greater than the lowest required LoQ in dried cannabis flower makes for a substantial analytical challenge [3, 28]. Overcoming these challenges often requires the use of two analytical separation techniques (typically liquid and gas chromatography) coupled to sensitive and specific detectors capable of distinguishing between coeluting analytes [29–32]. Blais et al. have employed QuEChERS (Quick, Easy, Cheap, Effective, Rugged, and Safe) extractions for quantifying pesticides in cannabis leaves, flowers, and oil, noting QuEChERS to be an effective strategy in cannabis leaves and oil, but resulted in high matrix effects that prevented reliable quantification of the GC-amenable analytes on Canada’s list of pesticides in cannabis flowers [29]. This prior knowledge has led us to develop an alternative extraction method.

Herein, we report the development and validation of LC-MS/MS and GC-MS/MS methods for a combined 96 pesticides in dried cannabis flower using 10 internal standards and a blend of 3 dried cannabis varieties from licenced producers in Ontario ranging widely in major cannabinoid content, used as a blank matrix for matrix-matched calibration. This blend has allowed us to develop our methods with approximately average levels of major interferences and evaluate its efficacy in more extreme cases by applying the methods to individual cultivars as well as ground whole-plant hemp. Further, we report the linearity, precision, accuracy, LoQs, recovery, ion suppression, and between-sample accuracy of our LC-MS/MS and GC-MS/MS methods for the determination of pesticide concentrations in dried cannabis inflorescence. The presented methods were also applied to six dried cannabis samples recently seized from illegal storefronts by the Ontario Provincial Police, identifying unauthorized pesticide residues in all samples tested.

## Materials and methods

### Chemicals, materials, and standards

All solvents were Optima™ grade and purchased from Fisher Scientific (Ottawa, Ontario, Canada) unless otherwise stated. Pesticide standards were purchased from SPEX CertiPrep® (Metuchen, NJ, USA) and encompass the entire suite of pesticides unauthorized in Canada for use on cannabis. Isotopically labelled pesticides were purchased from Toronto Research Chemicals (Toronto, Ontario, Canada) and Millipore-Sigma (Oakville, Ontario, Canada), and used without further purification. Cannabis was cultivated and prepared by licenced producers in Canada and was purchased from the Ontario Cannabis Store (https://ocs.ca).

### Instrumentation

Particle size reduction and homogenization of dried cannabis flowers were accomplished using a SPEX 6875D Freezer Mill (Metuchen, NJ, USA). Extraction was carried out using a Fisherbrand™ (Fisher Scientific Company, Ottawa, ON, CAN) multi-tube vortexer and centrifuge. A Fisherbrand™ accuSpin™ Micro 17R microcentrifuge (Fisher Scientific Company, Ottawa, ON, CAN) was used to pellet the dispersive solid-phase extraction (dSPE) solids in the GC-MS/MS cleanup step. LC-MS/MS was carried out using a Vanquish Flex UHPLC system coupled to a TSQ Altis triple quadrupole mass spectrometer (Thermo Scientific, San Jose, CA, USA) with the settings outlined in Table S1. The Vanquish LC is equipped with a 150-µL solvent mixing chamber, an Acclaim Trinity Q1 (5 µm, 120 Å, 10 mm) guard column, and an Acclaim Polar Advantage II (2.2 µm, 120 Å, 2.1 mm × 100 mm) reverse phase analytical column. A 16-min gradient elution is employed according to Table S2 using 3 mM ammonium formate in ultrapure water + 0.1% formic acid as mobile phase A and 3 mM ammonium formate in methanol + 0.02% formic acid as mobile phase B. GC-MS/MS was carried out using a Trace 1310 GC (Thermo Scientific, San Jose, CA, USA) coupled to a TSQ 9000 triple quadrupole mass spectrometer (Thermo Scientific, San Jose, CA, USA) with the settings outlined in Table S3. The TSQ 9000 is equipped with a splitless PTV injector, Restek (Restek Corporation, Bellefonte, PA, USA) Topaz 6 baffled liner (2 mm I.D., 2.75 mm O.D. × 120 mm) and a TG-5SILMS (30 m × 0.25 mm × 0.25 µm film) GC column connected to the MS transfer line using a SilTite nut and ferrule. A 30.3-min method is employed using 1.2 mL/min of helium carrier gas with an oven temperature ramping stepwise from 40 to 325 °C over the duration of the method. The specific inlet and oven temperature programs can be found in Tables S4 and S5. LC-MS/MS MRM and GC-MS/MS SRM transitions can be found in Tables S6 and S7, respectively. Integration and data analysis were completed using Chromeleon 7.2.9 and 7.2.10 (Thermo Scientific, San Jose, CA, USA) software.

### Sample preparation—extraction of pesticides from dried cannabis flower

A Master Calibration Solution (8.33 µg/mL in acetonitrile) of pesticides was prepared by combining the solutions provided in the SPEX Canadian Pesticide kit (100 µg/mL for 94 of the analytes and 1000 µg/mL for naled) and additional daminozide (1000 µg/mL) standard. This stock solution was diluted in acetonitrile 1–100 fold to prepare a series of spiking solutions appropriate for each calibration level according to Tables S8 and S9. An internal standard spiking (ISS) solution containing 2 ng/µL of isotopically labelled myclobutanil (d_4_), piperonylbutoxide (d_9_), diazinon (d_10_), trans-permethrin (d_6_), and thiamethoxam (d_4_), as well as 3 ng/µL of daminozide (d_4_), deltamethrin (d_5_), and quintozene (^13^C_6_), and 4 ng/µL of kresoxim-methyl (d_7_) and imazalil (d_5_) was prepared according to Table S10. Dried cannabis flower was reduced to a fine powder under liquid nitrogen using a SPEX freezer mill programmed for 3 2-min milling cycles separated by 2-min resting periods. One gram of each milled sample was weighed into 15-mL polypropylene centrifuge tubes and spiked with 60 µL of the appropriate spiking solution for the 5–500 ppb standards followed by 100 µL of the internal standard spiking solution. The 1000–2000 ppb standards require larger volumes of spiking solutions, which can be found in Table S11. Samples were shaken by hand to facilitate mixing of the matrix and the spiking solutions. After mixing, 5 mL of acetonitrile + 1% acetic acid was transferred to each tube via mechanical pipette followed by vortexing at 2500 rpm for 5 min and centrifugation at 5000 rpm (4696 RCF) for 5 min. A 3.6 mL aliquot of the supernatant is passed through a 500-mg (6 mL) Hypersep™ C_18_ SPE (Thermo Scientific, San Jose, CA, USA) and partitioned for either dilution and analysis by LC-MS/MS, or additional cleanup using dispersive SPE prior to GC-MS/MS analysis. Enough supernatant is produced from a single extraction to allow for both GC- and LC-MS/MS analysis to be performed on the same extracted sample.

### LC-MS/MS extract cleanup

A 3.6 mL aliquot of the crude extract obtained from the procedure in the “Sample preparation—extraction of pesticides from dried cannabis flower” section is pushed through a 500-mg HyperSep™ C_18_ (Thermo Fisher Scientific, MA, USA) solid-phase extraction tube by applying positive pressure using a 24-mL syringe fitted with a flexible septum. The effluent is vortexed and diluted 2-fold in a 20:80 mixture of LC mobile phase A:mobile phase B. One microliter of the cleaned and diluted extract is injected on the LC-MS/MS system for analysis.

### GC-MS/MS extract cleanup

An 800 µL aliquot of the cleaned extract obtained in the “Sample preparation—extraction of pesticides from dried cannabis flower” section is transferred to a HyperSep™ dispersive solid-phase extraction microcentrifuge tube containing 150 mg MgSO_4_, 50 mg primary-secondary amine (PSA), and 50 mg C_18_ (Thermo Fisher Scientific, MA, USA). The dSPE tube is vortexed for 90 s, centrifuged at 13,000 rpm (17,000 RCF) for 5 min. One hundred microliters of the supernatant is transferred to a 2-mL silanized amber glass autosampler vial and diluted 10× with toluene and vortexed. One microliter of the cleaned and diluted extract is injected on the GC-MS/MS system for analysis.

### Validation data analysis

Linearity was evaluated using weighted (1/×) linear regression of a minimum of five calibration points using peak-area ratios (analyte area/internal standard area) of the analyte to the nearest-eluting internal standard. In situations where there is a large difference in recovery of an analyte and its nearest-eluting internal standard (e.g., acephate and daminozide-d_4_), the next nearest-eluting internal standard is used. Several related analytes separate chromatographically, requiring separate integration of each signal and naming them to a unique group. In these cases, Chromeleon combines the integration of all signals within the group and generates a single calibration curve. Mevinphos, for example (Figure S1), is an insecticide made up of two geometric isomers that differ only by the orientation of a C=C double bond. In the present LC-MS/MS method, the two isomers are baseline separated by ~40 s and both contribute to the minimum method performance limit of 50 ppb for the quantitation of mevinphos, as required by the Canadian regulations. Integration in this manner allows accurate quantitation of chromatographically separated isomers without prior knowledge of the concentration ratios of the individual isomers. Concentrations for seven LC-MS/MS and five GC-MS/MS analytes are calculated in this manner and are referred to as “group analytes” in this work. Precision was evaluated as the coefficient of variation (%CV) of replicate quality control (QC) injections carried out over the course of validation. QC samples were prepared in the same cannabis blend as the calibrants and were spiked with pesticides at 25, 75, 250, and 1250 ppb. Within-blend accuracy was evaluated as the percent difference between the measured concentration values and the calculated concentration values of QC samples above an analyte’s LoQ while correcting for the exact mass of cannabis used in a given extraction. LoQs were determined from the slope and standard error of the residuals of the regression line for each analyte according to Eq. 1, below [33, 34].

1 $$\mathrm{LoQ}=\frac{10*\text{Standard Error}}{\text{Slope of Analyte Regression Line}}$$

Between-sample accuracy was evaluated in the same manner as within-blend accuracy: we spiked 10 cannabis cultivars (termed CS-1 through 10) varying in major cannabinoid content (ranging from 0 – 272 mg/g total THC and 0 – 146 mg/g total CBD) at 25 ppb, 75 ppb, 250 ppb, and 1250 ppb, and subjected six samples to each instrument with two cultivars (CS-1 and CS-6), hemp and the calibration blend overlapping both methods. Recovery was evaluated by comparing the mean area counts (*n*=3) of QC samples spiked prior to extraction to blank cannabis extracts spiked post-extraction. Ion suppression was evaluated by comparing mean area counts of cannabis extracts spiked post-extraction (*n*=3) to clean extraction solvent in the absence of cannabis matrix.

## Results and discussion

### Linearity

Matrix-matched calibration (linear, 1/× weighted) using peak-area ratios of the analyte to its associated internal standard afforded high degrees of linearity (*R*^2^ > 0.99) over 3–4 orders of magnitude, depending on the analyte’s LoQ. Peak-area ratios were used to correct for losses during the extraction process, and the nearest-eluting internal standards were used to compensate for ion suppression/enhancement that may be occurring in areas of the chromatogram nearest the target analyte. *R*^2^ values and calibration curves for all analytes can be found in the supplementary information (Figures S2–S12). Linearity of many GC-MS/MS analytes are influenced by the cleanliness of the GC-MS/MS system, particularly the PTV inlet liner and the analytical column. The analytical column can be baked out periodically to restore its performance, while the inlet liner and septum are replaced prior to each sequence. Degradation of the instrument response for some analytes (particularly etridiazole) has been observed as a function of the number of injections performed. Therefore, GC-MS/MS sequences have been limited to 75 injections (~41.25 h) each to allow for regular consumable replacement.

### Precision

The same blank matrix blend that was used for calibration was spiked at three levels for LC-MS/MS and four levels for GC-MS/MS within the calibration ranges. Twenty-five parts per billion was chosen to evaluate the precision of the extraction and instrument response nearing 20 ppb, which is the most frequent quantitation limit stipulated by the Canadian Regulations. Thus, 75, 250, and 1250 ppb were chosen to evaluate the precision of pesticides that yielded lower instrumental response, such as azadirachtin, allethrin, fenvalerate, and kinoprene whose LoQ requirements approach or exceed 25 ppb. Each QC sample was injected a minimum of three times in each sequence and the mean, standard deviation, and %CV for each sequence were calculated. The mean of the %CV values was calculated and reported as the precision in Tables 1 and 2 for individual LC-MS/MS analytes and Table 3 for individual GC-MS/MS analytes. Generally, precision across all analytes and concentrations falls below 10%CV, with the extremes being 2.9 %CV (etoxazole, 75 ppb) and 38%CV (azadirachtin, 25 ppb). Coextracted cannabinoids elute with retention times between 7 and 10 min, offering a potential explanation for increased %CV for late eluting analytes.

**Table 1:** Precision of LC-MS/MS analytes expressed as the %CV of 11 replicate injections at 25 ppb, 75 ppb, and 250 ppb stemming from three separate extractions carried out on three separate days. Analytes are listed in order of elution. “BLQ” indicates that the indicated concentration falls below the analyte’s limit of quantitation

	%CV (*n*=11)				%CV (*n*=11)		
Analyte	25 ppb	75 ppb	250 ppb	Analyte	25 ppb	75 ppb	250 ppb
Daminozide	BLQ	12.3	4.7	Bifenazate	9.7	5.3	4.3
Acephate	6.6	5.3	8.0	Tetrachlorvinphos	5.4	5.9	7.3
Dinotefuran	4.7	3.8	5.2	Kresoxim methyl	16.1	12.5	8.5
Oxamyl (+NH4)	4.6	3.7	5.0	Tebufenozide	12.4	10.7	8.9
Methomyl	5.4	5.9	5.8	Propiconazole	BLQ	6.8	5.9
Flonicamid	7.2	5.8	7.4	Cyprodinil	13.0	8.0	10.8
Thiamethoxam	3.6	5.8	6.3	Diazinon	2.9	6.7	4.6
Pirimicarb	2.9	5.2	4.4	Fenoxycarb	6.6	6.4	10.0
Imidacloprid	7.1	4.6	5.7	Fludioxonil	20.1	9.5	9.3
Dimethoate	5.8	4.1	6.0	Tebuconazole	BLQ	6.3	9.3
Clothianidin	3.4	3.5	6.0	Fenthion	7.3	6.7	6.6
Acetamiprid	6.0	3.6	5.9	Fipronil	8.9	6.0	4.9
Aldicarb (+NH4)	17.3	8.1	6.8	Benzovindiflupyr	10.0	6.4	3.6
Thiacloprid	4.6	3.5	5.3	Prallethrin	BLQ	20.3	6.0
Dichlorvos	6.5	5.5	5.7	Pyraclostrobin	5.2	4.9	5.1
Propoxur	5.5	4.1	5.3	Trifloxystrobin	5.1	4.8	5.4
Carbofuran	5.7	4.4	7.0	Coumaphos	4.8	5.8	4.3
Imazalil	15.4	14.5	6.8	Buprofezin	6.3	5.0	4.7
Dodemorph	13.0	5.8	4.9	Piperonyl butoxide (+NH4)	6.6	6.6	3.8
Thiophanate methyl	7.7	5.0	9.9	Clofentezine	BLQ	8.0	6.4
Metalaxyl	6.0	3.2	3.8	Allethrin	BLQ	15.5	8.6
Azadirachtin	37.4	13.3	6.2	Novaluron	8.7	6.1	8.1
Carbaryl	7.3	5.6	7.1	Spiromesifen (+NH4)	9.2	4.6	4.8
Spiroxamine	7.9	4.2	9.0	Etoxazole	5.7	2.9	4.8
Cyantraniliprole	7.3	3.4	6.5	Hexythiazox	5.1	5.5	7.1
Fensulfothion	6.4	5.5	6.8	Chlorpyrifos	6.0	4.2	4.9
Naled	4.8	5.0	5.8	Fenpyroximate	4.9	4.5	4.2
Azoxystrobin	7.3	5.0	6.6	Spirodiclofen	15.2	13.8	6.3
Chlorantraniliprole	10.2	7.5	8.9	Abamectin B1a (+NH4)	BLQ	BLQ	12.6
Phosmet	4.2	5.7	8.6	Teflubenzuron	BLQ	19.6	10.1
Iprodione	BLQ	6.3	8.4	Methoprene	18.9	8.8	6.2
Spirotetramat	9.3	4.0	5.5	Pyridaben	6.3	3.4	5.6
Malathion	6.3	7.6	7.9	Deltamethrin (+NH4)	14.2	5.9	5.6
Methiocarb	6.2	3.0	7.5	Fenvalerate	BLQ	26.6	12.1
Ethoprophos	6.8	5.4	5.1	trans-Permethrin (+NH4)	BLQ	8.8	5.4
Paclobutrazol	17.8	10.8	4.3	Phenothrin	15.4	7.0	3.6
Myclobutanil	7.1	5.6	5.8	Etofenprox (+NH4)	5.5	5.8	4.8
Boscalid	14.0	7.6	7.5	Bifenthrin (+NH4)	33.1	6.1	3.8
Fluopyram	4.3	4.2	5.8	Acequinocyl (+NH4)	10.0	3.5	3.9

**Table 2:** Precision of LC-MS/MS group–calibrated analytes expressed as the %CV of 11 replicate injections at 25 ppb, 75 ppb, and 250 ppb stemming from 3 separate extractions carried out on 3 separate days. Analytes are listed in order of elution

	%CV (*n*=11)		
Analyte	25 ppb	75 ppb	250 ppb
Dimethomorph group	4.8	6.3	7.6
Mevinphos group	7.3	6.7	6.5
Pyrethrins	BLQ	27.0	9.9
Resmethrins	BLQ	6.5	3.9
Spinetoram group	BLQ	12.0	8.3
Spinosad	BLQ	6.6	7.9
Tetramethrins	16.4	8.5	4.7

**Table 3:** Precision of GC-MS/MS analytes expressed as the %CV of 23 replicate injections at 25 ppb, 75 ppb, 250 ppb, and 1250 ppb stemming from 3 separate extractions carried out on 3 separate days. Analytes are listed in order of elution

	%CV (*n*=23)			
Analyte	25 ppb	75 ppb	250 ppb	1250 ppb
Etridiazole	7.6	6.1	6.3	3.3
Quintozene	13.5	11.2	8.2	3.3
Diazinon	8.9	5.9	5.7	3.5
Methyl parathion	8.8	6.4	4.2	3.0
Kinoprene	BLQ	BLQ	BLQ	4.9
Chlorpyrifos	25.0	4.5	5.5	3.5
Fenthion	10.4	9.0	4.7	2.7
Endosulfan alpha	14.5	6.4	5.8	3.6
Chlorfenapyr	BLQ	16.0	7.4	4.1
Endosulfan beta	17.9	11.7	8.2	4.6
Endosulfan sulfate	5.7	4.4	4.7	4.4
Etofenprox	BLQ	7.3	5.8	4.8

Group analyte data was treated analogously using the group calibration strategy described in the “Linearity” section. Precision similar to individual analytes was observed and is tabulated in Table 2 (LC-MS/MS) and Table 4 (GC-MS/MS) with %CVs ranging from 3.9% (resmethrin, 250 ppb) to 27.0% (pyrethrins, 75 ppb). The %CV for pyrethrins is larger than for other group analytes primarily due to pyrethrin I eluting as the shoulder of an isobaric matrix component, reducing the precision of pyrethrin analysis.

**Table 4:** Precision of GC-MS/MS group-calibrated analytes expressed as the %CV of 23 replicate injections at 25 ppb, 75 ppb, 250 ppb, and 1250 ppb stemming from 3 separate extractions carried out on 3 separate days. Analytes are listed in order of elution

	%CV (*n*=23)			
Analyte	25 ppb	75 ppb	250 ppb	1250 ppb
Cyfluthrin group	BLQ	10.1	8.3	8.0
Cypermethrin group	BLQ	BLQ	10.0	7.2
Fenvalerate group	BLQ	5.6	5.0	5.6
MGK group	6.8	5.4	4.7	2.9
Permethrin group	BLQ	BLQ	5.3	4.8

### Accuracy

#### Within-blend

Within-blend accuracy was evaluated as the difference between the measured value and the calculated value at each of the QC levels, expressed as the percent difference from the calculated value using Eq. 2. The comparison was made using the same cannabis blend used to build the calibration curve, ensuring minimal matrix variation.

2 $$\mathrm{Accuracy}=1-\left(\frac{{C}_{\mathrm{Measured}}-{C}_{\mathrm{Calculated}}}{{C}_{\mathrm{Calculated}}}\right)$$

Accuracy data was collected over a minimum of three sequences with a minimum of three injections at each QC level in each sequence. The data was combined via weighted mean based on the number of replicate injections in a given sequence to ensure that each injection contributed equally to the reported accuracy values. Within-sample accuracy tends to increase with concentration and is negatively impacted by the presence of co-extracted matrix components. Within the calibration blend, accuracy values ranging from 85.5 to 111.3% are observed above an analyte’s LoQ. Within-sample accuracy results are tabulated in the rightmost column of Tables 5, 6, 7, and 8 alongside the between-sample accuracy results.

**Table 5:** Accuracy of individual LC-MS/MS analytes spiked in dried cannabis inflorescence purchased from the Ontario Cannabis Store. Cannabis was spiked at 75 ppb in each cultivar

Analyte	CS-1	CS-2	CS-3	CS-4	CS-5	CS-6	Hemp	Cal blend
THCA (mg/g)	127	5.0	0.4	146	272	47.1	1.0	57.3
CBDA (mg/g)	0.3	146	56.4	0.4	0.6	108	14.6	84.5
Accuracy (%)								
Daminozide	122.0	95.6	85.5	112.0	122.2	108.4	87.1	93.6
Acephate	104.9	110.5	109.4	110.2	105.5	103.7	110.8	110.0
Dinotefuran	97.5	107.4	103.8	103.0	103.0	103.1	101.4	107.7
Oxamyl (+NH_4_)	102.8	106.1	106.8	103.1	98.9	102.0	105.6	104.7
Methomyl	102.6	105.7	111.6	107.7	99.7	103.5	103.7	108.3
Flonicamid	90.8	97.5	95.8	104.6	97.8	100.0	97.2	103.9
Thiamethoxam	99.0	99.5	101.5	97.0	102.3	94.3	102.2	104.1
Pirimicarb	95.9	108.2	94.4	96.2	115.4	98.0	97.7	103.5
Imidacloprid	103.3	100.7	95.3	97.1	98.8	98.2	92.4	105.9
Dimethoate	98.4	106.3	98.8	102.1	101.1	97.7	103.6	105.6
Clothianidin	100.8	103.0	99.6	104.5	104.7	100.2	89.4	106.3
Acetamiprid	103.3	105.5	99.7	104.6	102.4	98.3	94.2	107.6
Aldicarb (+NH_4_)	100.9	98.2	95.7	98.3	97.7	90.8	96.3	103.0
Thiacloprid	104.6	98.9	90.9	104.1	108.5	92.6	87.9	105.0
Dichlorvos	103.7	101.7	99.0	104.1	106.7	103.9	103.3	109.8
Propoxur	104.9	107.5	102.4	102.7	107.1	100.8	101.9	107.0
Carbofuran	95.7	104.8	101.0	100.4	102.1	94.2	97.3	103.7
Imazalil	110.4	99.2	88.9	101.6	121.3	104.1	99.0	98.2
Dodemorph	150.2	86.4	104.7	95.0	105.2	94.2	109.7	96.7
Thiophanate methyl	125.1	78.4	68.8	87.7	63.0	92.2	130.6	104.3
Metalaxyl	90.8	104.3	104.1	101.7	105.1	104.0	80.7	101.6
Azadirachtin	69.4	82.5	105.3	100.4	54.0	89.1	71.0	97.9
Carbaryl	84.8	106.8	100.9	97.5	96.2	98.4	91.6	105.1
Spiroxamine	137.1	72.1	75.5	154.8	168.7	102.1	82.5	95.9
Cyantraniliprole	76.3	116.8	95.6	90.8	77.7	98.4	68.7	101.0
Fensulfothion	101.6	107.0	114.2	107.9	112.2	108.0	91.5	102.3
Naled	91.6	104.6	93.8	106.2	102.3	94.3	86.5	102.3
Azoxystrobin	117.4	107.7	109.6	118.7	124.0	95.1	76.2	106.4
Chlorantraniliprole	113.6	104.2	103.7	109.0	111.2	99.5	99.2	102.6
Phosmet	96.0	94.6	95.9	103.1	95.9	91.2	90.8	104.2
Iprodione	126.3	86.9	92.8	108.2	113.9	97.0	77.3	103.7
Spirotetramat	119.2	81.3	88.4	103.2	100.1	94.3	94.7	104.2
Malathion	97.3	107.1	108.0	102.9	111.0	104.2	98.5	106.0
Methiocarb	96.2	110.4	108.0	103.6	106.0	103.5	98.1	103.1
Ethoprophos	98.5	105.4	107.6	105.8	106.5	97.3	101.7	104.2
Paclobutrazol	118.2	101.1	93.7	111.4	123.6	99.6	113.7	104.1
Myclobutanil	99.6	102.5	97.7	102.0	112.5	102.8	96.1	100.8
Boscalid	98.1	108.4	103.7	104.1	103.7	97.2	−8.8	103.1
Fluopyram	84.9	102.6	120.7	89.5	84.9	110.1	96.9	102.4
Bifenazate	97.4	105.7	116.6	97.5	99.9	106.3	94.6	102.3
Tetrachlorvinphos	112.9	99.8	102.2	103.2	104.0	106.8	134.6	105.9
Kresoxim methyl	110.3	82.8	100.0	103.6	101.9	104.4	135.4	97.6
Tebufenozide	112.0	96.2	109.2	102.7	100.2	103.3	137.1	103.1
Propiconazole	138.8	95.4	101.8	111.4	134.5	117.9	102.6	107.1
Cyprodinil	134.7	83.9	74.2	121.1	144.0	101.2	72.4	106.1
Diazinon	105.9	102.4	104.4	104.8	104.1	103.1	102.7	103.9
Fenoxycarb	123.8	100.2	104.4	112.9	120.1	106.6	139.5	108.9
Fludioxonil	92.9	113.6	122.5	91.9	82.4	84.0	115.7	94.6
Tebuconazole	127.2	105.6	108.0	114.0	132.1	101.8	120.8	111.1
Fenthion	120.7	101.7	112.1	109.6	116.1	96.7	108.5	110.9
Fipronil	102.0	109.0	103.4	110.8	99.3	103.7	97.4	98.9
Benzovindiflupyr	115.0	96.6	109.9	112.1	112.0	94.2	105.6	102.6
Prallethrin	117.3	100.2	103.4	87.1	145.9	92.9	8.0	101.8
Pyraclostrobin	108.9	97.7	110.3	113.1	106.0	94.4	105.8	104.4
Trifloxystrobin	105.2	102.8	107.3	107.8	99.5	96.2	109.0	103.0
Coumaphos	116.7	93.2	103.7	104.2	110.8	90.8	95.3	105.1
Buprofezin	103.5	100.5	103.6	105.2	110.4	102.0	103.7	104.4
Piperonyl butoxide (+NH_4_)	107.0	105.3	106.9	108.0	103.1	99.1	97.9	103.2
Clofentezine	75.2	82.0	111.7	41.0	33.6	88.3	75.2	102.1
Allethrin	67.6	92.6	35.1	45.3	23.9	98.2	21.5	91.6
Novaluron	150.9	99.9	92.8	94.6	128.1	90.4	151.7	101.2
Spiromesifen (+NH_4_)	107.7	114.5	106.8	101.8	122.6	107.5	100.7	101.7
Etoxazole	99.5	111.4	109.2	96.5	110.7	111.3	99.4	104.8
Hexythiazox	131.5	112.9	121.5	97.5	116.4	96.9	138.8	104.9
Chlorpyrifos	118.8	109.5	114.1	99.2	117.0	105.5	110.8	104.3
Fenpyroximate	86.5	117.0	90.7	80.6	124.8	114.3	86.0	103.0
Spirodiclofen	100.8	122.8	105.1	102.0	132.0	121.4	64.6	102.4
Abamectin B1a (+NH_4_)	BLQ	BLQ	BLQ	BLQ	BLQ	BLQ	BLQ	BLQ
Teflubenzuron	136.1	114.8	43.0	96.8	118.4	102.7	110.8	88.9
Methoprene	109.7	105.9	108.5	101.9	104.9	102.2	104.4	99.4
Pyridaben	108.5	107.7	100.6	100.2	108.3	103.8	110.2	102.7
Deltamethrin (+NH_4_)	115.0	97.9	100.4	109.2	103.9	95.2	98.9	102.7
Fenvalerate	105.7	113.4	108.2	102.5	119.0	95.2	88.8	97.0
trans-Permethrin (+NH_4_)	72.1	104.2	99.0	92.6	100.5	103.4	100.3	102.8
Phenothrin	115.5	113.6	99.8	116.0	134.3	95.8	121.4	104.1
Etofenprox (+NH_4_)	−71.0	128.1	19.9	−129.8	−104.6	129.0	−110.8	103.3
Bifenthrin (+NH_4_)	93.7	125.7	105.2	103.4	137.4	102.9	55.8	103.2
Acequinocyl (+NH_4_)	82.5	105.9	109.8	108.7	114.0	110.1	117.2	101.8

**Table 6:** Accuracy of group-calibrated LC-MS/MS analytes spiked in dried cannabis inflorescence purchased from the Ontario Cannabis Store. Cannabis was spiked at 75 ppb in each cultivar

Analyte	CS-1	CS-2	CS-3	CS-4	CS-5	CS-6	Hemp	Cal blend
THCA (mg/g)	127	5.0	0.4	146	272	47.1	1.0	57.3
CBDA (mg/g)	0.3	146	56.4	0.4	0.6	108	14.6	84.5
Accuracy (%)								
Dimethomorph group	127.2	96.6	97.5	112.1	121.3	93.5	94.8	104.5
Mevinphos group	101.2	104.3	103.9	103.3	97.2	100.3	101.8	103.0
Pyrethrins	35.4	99.9	128.1	117.6	115.7	88.2	68.2	105.1
Resmethrins	67.8	104.4	84.8	71.5	83.6	90.3	54.3	102.4
Spinetoram group	131.0	81.1	106.6	117.0	132.1	112.8	162.9	97.8
Spinosad group	113.1	84.7	102.5	110.9	117.4	116.7	158.7	96.7
Tetramethrins	93.8	90.3	76.6	90.0	93.0	92.0	54.3	99.6

**Table 7:** Accuracy of individual GC-MS/MS analytes in dried cannabis inflorescence purchased from the Ontario Cannabis Store. Cannabis was spiked at 250 ppb in each cultivar

Analyte	CS-1	CS-6	CS-7	CS-8	CS-9	CS-10	Hemp	Cal blend
THCA (mg/g)	127	47.1	3.4	37	177	44.5	1.0	57.3
CBDA (mg/g)	0.3	108	135	102	0.4	87.5	14.6	84.5
Accuracy (%)								
Etridiazole	94.1	92.3	97.0	104.3	99.3	104.3	110.0	100.5
Quintozene	101.7	95.5	106.7	98.5	96.8	98.5	94.5	97.9
Diazinon	88.9	95.9	94.1	105.2	102.9	105.2	95.8	99.3
Methyl parathion	102.5	101.1	105.6	109.1	103.5	109.1	111.7	97.1
Chlorpyrifos	92.3	94.5	97.2	105.9	101.4	105.9	88.7	99.6
Kinoprene	BLQ	BLQ	BLQ	BLQ	BLQ	BLQ	BLQ	BLQ
Fenthion	88.8	96.6	97.3	101.2	94.5	101.2	108.4	98.6
Endosulfan alpha	95.9	87.9	96.3	99.9	102.7	99.9	86.8	97.6
Chlorfenapyr	102.5	99.5	105.8	95.3	120.5	95.3	94.5	98.1
Endosulfan beta	91.6	97.8	98.6	108.6	103.8	108.6	87.4	100.3
Endosulfan sulfate	90.7	100.6	93.0	103.4	101.4	103.4	86.5	96.9
Etofenprox	96.2	104.7	100.7	103.2	97.1	103.2	93.3	97.9

**Table 8:** Accuracy of group-calibrated GC-MS/MS analytes spiked in dried cannabis inflorescence purchased from the Ontario Cannabis Store. Cannabis was spiked at 250 ppb in each cultivar

Analyte	CS-1	CS-6	CS-7	CS-8	CS-9	CS-10	Hemp	Cal blend
THCA (mg/g)	127	47.1	3.4	37	177	44.5	1.0	57.3
CBDA (mg/g)	0.3	108	135	102	0.4	87.5	14.6	84.5
Accuracy (%)								
Cyfluthrin group	105.4	114.6	102.7	103.5	91.1	103.5	90.6	98.3
Cypermethrin group	102.6	116.2	107.9	95.8	80.3	95.8	78.2	95.6
Fenvalerate group	98.5	110.8	110.4	98.6	88.8	98.6	84.1	97.8
MGK group	92.1	100.2	98.2	109.4	102.1	109.4	94.7	97.9
Permethrin group	105.9	127.3	97.3	99.5	77.9	99.5	124.3	95.1

#### Between-sample accuracy

Cannabis is a diverse species, and routine testing will not allow for cultivar-specific matrix-matched calibration to be performed. An evaluation of accuracy in a variety of cultivars provides a realistic picture of the method’s performance in such a variable matrix when the calibration matrix differs from that of the samples. To address this variability, we spiked 10 cannabis cultivars (termed CS-1 through 10) varying in major cannabinoid content (ranging from 0 to 272 mg/g total THC and 0 to 146 mg/g total CBD) at 25 ppb, 75 ppb, 250 ppb, and 1250 ppb, and subjected six samples to each instrument with two cultivars (CS-1 and CS-6), hemp and the calibration blend overlapping both methods. Cannabinoid content of these cultivars was determined in-house by LC-MS/MS following the method reported previously by McRae et al. [35]. Accuracy was calculated using Eq. 2 where the measured analytes were quantified using a calibration curve spiked in the calibration blend. Accuracy within the calibration blend falls within ±15% of the calculated values while a wider range was observed when the sample matrix is varied. Etofenprox, in particular, showed a heavy dependence on the matrix when analyzed by LC-MS/MS with an accuracy of 129.0% at 75 ppb in CS-6 (47.1 mg/g THCA and 108 mg/g CBDA) and −129.8% in CS-4 (146 mg/g THCA and 0.4 mg/g CBDA) at the same concentration; an unexpected result when compared to 103.3% at the same level in the calibration blend. The accuracy of etofenprox analyzed by LC-MS/MS is consistently poor and negative over all QC concentrations employed in each cultivar tested whose THCA content exceeds that of the calibration blend (57.3 mg/g THCA and 84.5 mg/g CBDA). Interestingly, the accuracy of etofenprox by LC-MS/MS in hemp (1.0 mg/g THCA and 14.6 mg/g CBDA) is also poor and negative despite the relatively low cannabinoid content. These negative accuracy values have not been observed in the GC-MS/MS results (Tables 7 and 8), which appear to be less affected by matrix composition than those acquired by LC-MS/MS and make GC-MS/MS the more appropriate choice of instrumentation for etofenprox quantification using the present method. The peak shape of some LC-MS/MS signals for analytes eluting between 4.5 and 6.5 min may be negatively impacted by high THCA concentrations extracted from the matrix. Peak shapes can be restored in these instances by performing an additional 10-fold dilution in the same 20:80 (v/v) mixture of mobile phase A and mobile phase B, or by injecting a smaller sample volume. Data presented here for the more challenging pesticides was obtained using the standard protocol, without further dilution or reduced injection volume.

Group-calibrated analyte data was treated similarly and is tabulated in Tables 6 and 8. LC-MS/MS data for 25 ppb and 250 ppb as well as GC-MS/MS data for 25, 75, and 1250 ppb can be found in Tables S12–21 in the supplementary information.

### Ion suppression and recovery

The matrix-matched calibration, use of a range of internal standards and pre-extraction spiking approach presented in this method is designed to compensate for ion suppression and recovery by spiking dried cannabis with pesticide standard solutions at known concentrations and subjecting these matrix calibration standards to the same extraction and cleanup procedures as the samples. This strategy, coupled with the use of a blend of cultivars for calibration, aims to minimize the apparent effects of co-extracted matrix components and reduce the variability between the calibration standards and the test samples. Given the high degree of variability in cannabis biomass, however, it is prudent to examine the extraction recoveries and matrix effects observed by the analytes in various regions of the chromatogram. As such, recovery was evaluated by comparing peak area counts of each analyte in QC samples spiked prior to extraction to those spiked at the same concentration after extraction and sample cleanup using Eq. 3, where “Pre” refers to the area count of an analyte in a sample spiked with pesticides prior to extraction and “post” refers to that in a sample spiked with an equivalent concentration of pesticides after liquid-solid extraction and solid-phase extraction.

3 $$\mathrm{Recovery}=100\times (\frac{\mathrm{Pre}}{\mathrm{Post}})$$

**Fig. 1:**
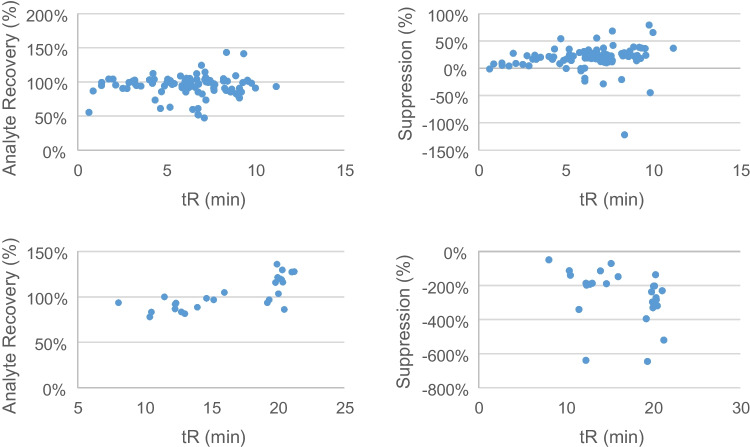
Recovery (upper left) and ion suppression (upper right) vs retention time for LC-MS/MS analytes at 75 ppb as well as recovery (lower left) and ion suppression (lower right) vs retention time for GC-MS/MS analytes at 250 ppb

Mean recoveries from samples spiked at 25, 75, and 250 ppb in cannabis and hemp are summarized in Fig. 1. The majority of analytes displayed recoveries of 60–120%, though recoveries dipped as low as 29% in cannabis and 13% in hemp for spinetoram L. Recovery data shows the importance of spiking calibrants prior to extraction in order to account for extraction losses when analyzing unknown samples.

Ion suppression was evaluated by comparing peak area counts of each analyte in extracts spiked post-extraction to area counts of clean extraction solvent spiked with the same concentration of analytes using Eq. 4, where “CS” refers to the area count of an analyte spiked in clean solvent and “Post” again refers to the area count of the same analyte spiked in extracted matrix following solid-phase extraction.

4 $$\text{Ion Suppression}=100\times (\frac{\mathrm{CS}-\mathrm{Post}}{\mathrm{CS}})$$

Ion suppression/enhancement versus retention time in cannabis is shown in the right-hand panels of Fig. 1. Positive values indicate ion suppression, while negative values indicate ion enhancement or suggest the presence of coeluting isobaric matrix components. Coextracted cannabinoids began to elute between 7 and 8 min under the current chromatographic conditions and are likely the major contributors to matrix-induced ion suppressing or enhancing effects given their abundance relative to the analytes. The data plotted in Fig. 1 can be found tabulated in the supplementary information (Tables S22–25). Ion suppression data show the importance of using a blank matrix closely resembling the test samples.

### Limits of quantitation

LoQs were calculated individually for each analyte from the slope and standard error of the regression line according to Eq. 1 [33, 34], and are listed in Table 9. The slope of the regression line and standard deviation of the calibration point residuals were computed by Chromeleon software. Standard error was calculated offline from the SD of the regression line residuals and the square root of the number of calibration points on the curve. LoQs were calculated from each sequence and the mean of all calculated values is reported. LoQ calculation in this manner provides larger LoQ values than those calculated by extrapolating signals from QC samples to a signal-to-noise ratio of 10 and is considered a more conservative approach as a result. Using this approach, some calculated LoQs fall above the Canadian LoQ requirements for a method quantifying pesticide residues in dried cannabis [3]. To demonstrate the method’s performance in these cases, the calibration blend of cannabis was spiked at 10, 20, and 50 ppb and extracted according to the procedure described above. Each extract was injected 10 times and the %CV was calculated for the area counts of each set of replicates, the results of which are presented in Table 10. The LoQ of a given analyte was deemed acceptable if the %CV ≤ 25 % at or below the published requirements, and all analyte LoQs verified this way meet or exceed the ≤ 25 %CV criterion. Therefore, we believe the method is fit-for-purpose to determine all pesticides at the method performance limits required by Health Canada [3].

**Table 9:** Calculated LoQs for analytes and their respective minimum method performance limit stipulated by Canadian cannabis regulations (see reference [3])

Analyte	LC-MS LoQ (ppb)	GC-MS LoQ (ppb)	Required LoQ (ppb)	Analyte	LC-MS LoQ (ppb)	GC-MS LoQ (ppb)	Required LoQ (ppb)
Abamectin B1a	87	-	100	Fluopyram	11	-	20
Acephate	16	-	20	Hexythiazox	13	-	10
Acequinocyl	16	-	30	Imazalil	19	-	50
Acetamiprid	14	-	100	Imidacloprid	14	-	20
Aldicarb	15	-	1000	Iprodione	51	-	1000
Allethrin	56	-	200	Kinoprene	-	315	500
Azadirachtin	20	-	1000	Kresoxim methyl	19	-	20
Azoxystrobin	14	-	20	Malathion	15	-	20
Benzovindiflupyr	9	-	20	Metalaxyl	14	-	20
Bifenazate	13	-	20	Methiocarb	13	-	20
Bifenthrin	16	-	1000	Methomyl	16	-	50
Boscalid	21	-	20	Methoprene	16	224	2000
Buprofezin	12	-	20	Methyl parathion	-	15	50
Carbaryl	14	-	50	Mevinphos group	13	-	50
Carbofuran	14	-	20	MGK-264 group	-	17	50
Chlorantraniliprole	12	-	20	Myclobutanil	13	-	20
Chlorfenapyr	-	26	50	Naled	13	-	100
Chlorpyrifos	15	18	40	Novaluron	16	-	50
Clofentezine	26	-	20	Oxamyl	13	-	3000
Clothianidin	12	-	50	Paclobutrazol	18	-	20
Coumaphos	14	-	20	Phenothrin	20	-	50
Cyantraniliprole	11	-	20	Phosmet	13	-	20
Cyfluthrins	-	49	200	Piperonyl butoxide	11	-	200
Cypermethrins	-	83	300	Pirimicarb	14	-	20
Cyprodinil	24	-	250	Prallethrin	47	-	50
Daminozide	38	-	100	Propiconazole	27	-	100
Deltamethrin	14	-	500	Propoxur	17	-	20
Diazinon	11	18	20	Pyraclostrobin	13	-	20
Dichlorvos	15	-	100	Pyrethrins	84	-	50
Dimethoate	12	-	20	Pyridaben	11	-	50
Dimethomorph Group	17	-	50	Quintozene	-	19	20
Dinotefuran	13	-	100	Resmethrins	32	-	100
Dodemorph	14	-	50	Spinetoram group	25	-	20
Endosulfan alpha	-	19	200	Spinosad	31	-	100
Endosulfan beta	-	21	50	Spirodiclofen	16	-	250
Endosulfan sulfate	-	17	50	Spiromesifen	11	-	3000
Ethoprophos	14	-	20	Spirotetramat	14	-	20
Etofenprox	12	29	50	Spiroxamine	19	-	100
Etoxazole	14	-	20	Tebuconazole	25	-	50
Etridiazole	-	19	30	Tebufenozide	14	-	20
Fenoxycarb	18	-	20	Teflubenzuron	42	-	50
Fenpyroximate	12	-	20	Tetrachlorvinphos	17	-	20
Fensulfothion	11	-	20	Tetramethrins	17	-	100
Fenthion	18	18	20	Thiacloprid	10	-	20
Fenvalerate	38	-	100	Thiamethoxam	9	-	20
Fipronil	14	-	60	Thiophanate Methyl	20	-	50
Flonicamid	13	-	50	Permethrins^a^	27	119	500
Fludioxonil	19	-	20	Trifloxystrobin	12	-	20

^a^LC-MS/MS method quantifies *trans*-permethrin, while the GC-MS/MS method quantifies both isomers

**Table 10:** Coefficients of variance of 10 replicate injections for analytes displaying calculated LoQ exceeds the minimum method performance limits in Canada. Spiked concentrations listed are the sums of the individual analytes making up a group (e.g., the sum of pyrethrin I and pyrethrin II in the 50 ppb condition is 50 ppb, rather than 50 ppb of each pyrethrin). Boscalid, clofentezine, and hexythiazox are present at the concentrations listed

Analyte	Calculated LoQ (ppb)	Required LoQ (ppb)	%CV (*n*=10)		
			10 ppb	20 ppb	50 ppb
Boscalid	21	20	17.5	6.6	7.6
Spinetoram J	25	20	10.5	12.7	8.3
Spinetoram L	25	20	32.3	19.4	22.5
Pyrethrin II	84	50	31.3	20.9	12.9
Clofentezine	26	20	9.8	8.8	3.4
Hexythiazox	13	10	4.3	6.5	3.9
Pyrethrin I	84	50	BLQ	36.8	14.7

## Application to unknown samples

**Fig. 2:**
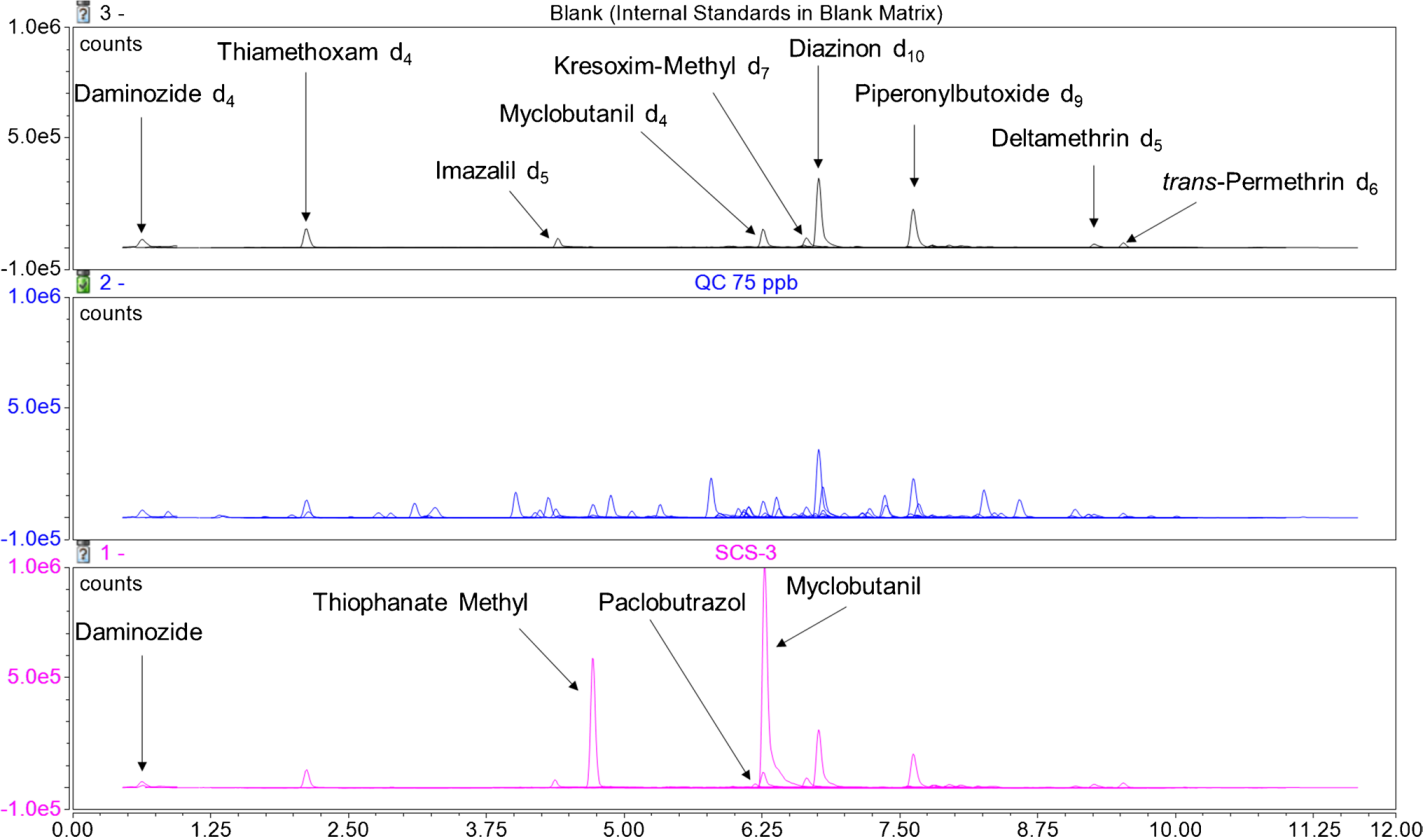
Stacked LC-MS/MS chromatograms of a blank matrix extract (internal standards only), a QC sample spiked at 75 ppb, and an illegal cannabis sample with incurred pesticide levels spiked with internal standards

**Fig. 3:**
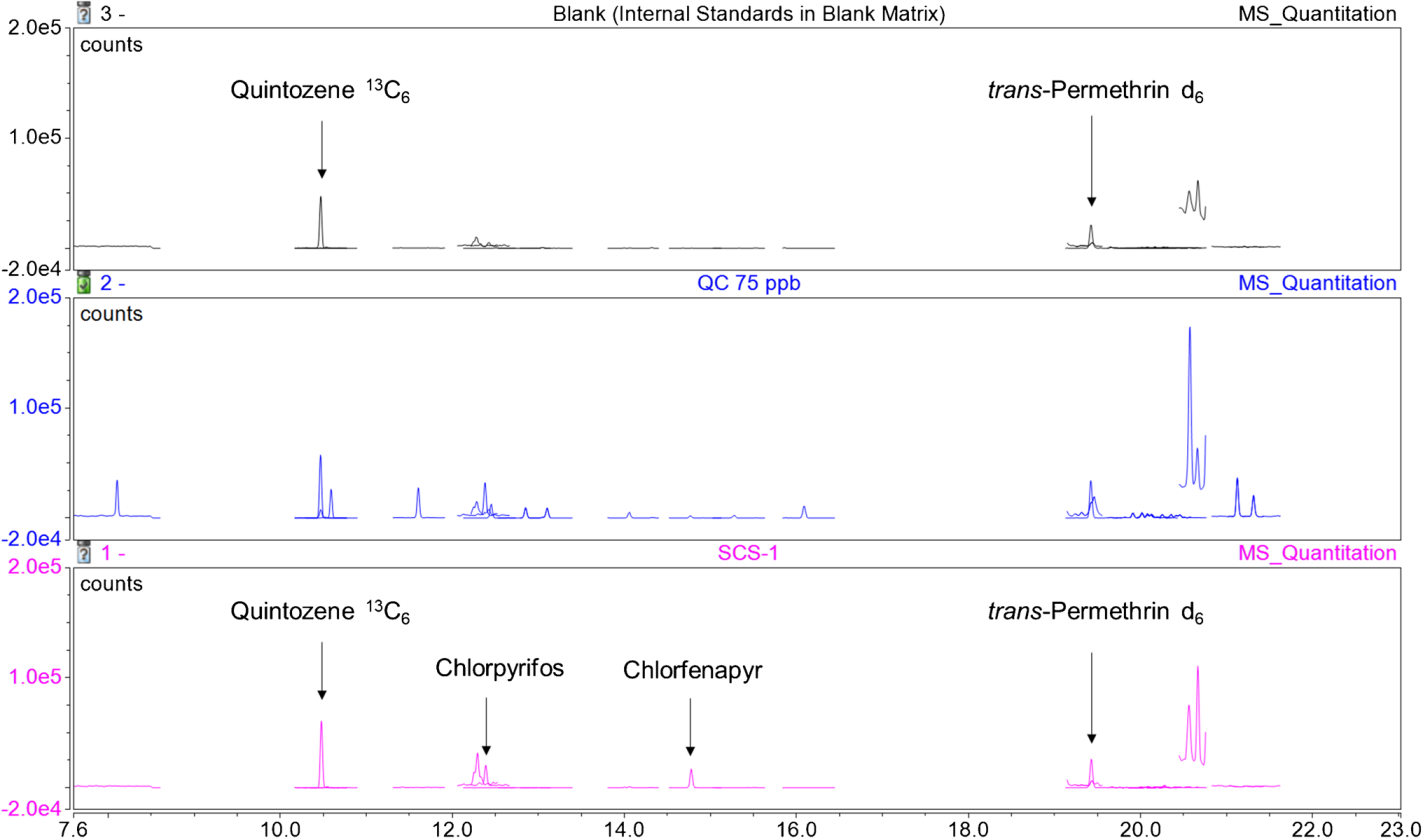
Stacked GC-MS/MS chromatograms of a blank matrix extract (internal standards only), a QC sample spiked at 75 ppb, and an illegal cannabis sample with incurred pesticide levels spiked with internal standards

A wide variety of cannabis samples purchased from the Ontario Cannabis Store were screened for pesticide content and employed in the development of this method. These legally produced samples did not display any detectable levels of the target analytes (data not shown). The current method was then applied to six samples of dried cannabis (termed SCS-1 through SCS-6) seized from illegal storefronts by the Ontario Provincial Police in an attempt to observe measurable pesticide levels. Figures 2 and 3 show the resulting stacked chromatograms of the blank sample condition spiked only with internal standards, a 75 ppb QC sample, and a selected illegal cannabis sample. The results of all unauthorized pesticides identified among the samples are tabulated in Table 11, below. SCS-3 was chosen as it displays daminozide and thiophanate methyl signals without being completely overshadowed by the size of the myclobutanil signal. Few GC-MS/MS-amenable pesticides were observed in the illegal samples, resulting in the selection of the SCS-1 chromatogram in Fig. 3, which shows signals for both chlorpyrifos and chlorfenapyr in addition to the internal standards. One sample, SCS-4, was found to contain only one of the analytes tested for, while 4 of the 6 samples were found to contain at least three unauthorized pesticide residues. Myclobutanil was found in every illegal sample tested and approached 10 ppm in its highest occurrence (SCS-1). SCS-1 was found to contain 10 unique pesticides and was not noticeably different from the other dried cannabis samples tested upon visual inspection. Both analytical methods agree on the quantitation of chlorpyrifos, the only residue identified in an illegal sample by both detection methods. SCS-1 was found to contain chlorpyrifos at a concentration of 45 ppb by LC-MS/MS, while the GC-MS/MS analysis of the same sample resulted in a measured concentration of 47 ppb. The discrepancy of 2 ppb between the two measurements shows excellent agreement between the two separation and detection techniques in this instance. All reported concentration values for analytes in excess of 1000 ppb were found via LC-MS/MS and were extrapolated from the standard curve. Since these high concentrations fall outside of our calibration range, their precision and accuracy are unknown and should be considered estimates.

**Table 11:** Concentrations of unauthorized pesticides (in ppb) identified in cannabis samples provided by the Ontario Provincial Police from illegal storefronts. BLQ indicates that the analyte was not observed, or was observed at concentrations below the analyte’s limit of quantitation

Analyte^a^	SCS-1	SCS-2	SCS-3	SCS-4	SCS-5	SCS-6
	Unauthorized pesticides identified by LC-MS/MS (ppb)					
Daminozide	BLQ	4915	790	BLQ	BLQ	40
Imidacloprid	23	BLQ	BLQ	BLQ	BLQ	BLQ
Thiophanate methyl	BLQ	BLQ	628	BLQ	BLQ	BLQ
Metalaxyl	105	BLQ	BLQ	BLQ	BLQ	BLQ
Paclobutrazol	255	629	278	BLQ	BLQ	BLQ
Myclobutanil	8276	7756	3462	357	27	4724
Piperonyl butoxide (+NH4)	486	BLQ	BLQ	BLQ	BLQ	461
Spiromesifen (+NH4)	20	BLQ	31	BLQ	BLQ	BLQ
Chlorpyrifos	45	BLQ	BLQ	BLQ	BLQ	BLQ
Pyridaben	46	BLQ	BLQ	BLQ	BLQ	BLQ
Pyrethrins	188	BLQ	BLQ	BLQ	BLQ	464
	Unauthorized pesticides identified by GC-MS/MS (ppb)					
Chlorpyrifos	47	BLQ	BLQ	BLQ	BLQ	BLQ
Endosulfan alpha	BLQ	BLQ	25	BLQ	24	BLQ
Chlorfenapyr	517	BLQ	187	BLQ	BLQ	806

^a^Values for LC-MS/MS analytes exceeding 1000 ppb fall outside the method’s calibration range and are considered estimates

## Summary

We have developed analytical methods that, when combined, are capable of quantifying each of the 96 analytes identified in Health Canada’s “Mandatory Cannabis Testing for Pesticide Active Ingredients – List and Limits” at or below the specified minimum method performance limits [3, 21]. The methods employ a common liquid-solid extraction and solid-phase extraction cleanup prior to dilution and analysis by LC-MS/MS or further cleanup by dispersive solid-phase extraction and subsequent dilution and analysis by GC-MS/MS. The linearity, precision, accuracy, recovery, ion suppression, and limits of quantitation of the method have been established using a blend of cannabis cultivars containing a balanced level of CBDA and THCA. Since cultivar-specific matrix-matched calibration is only possible under ideal circumstances, calibration has been established using a blend of cannabis samples designed to mimic an average of cultivars that might be sent to a testing facility. The accuracy of the method has been evaluated in hemp and 6 cannabis samples in addition to the calibration blend, finding that the accuracy of the GC-MS/MS method is less affected by cultivar differences than that of the LC-MS/MS method.

Application to six illegal cannabis samples from illegal storefronts in Ontario identified unauthorized pesticide residues in all samples tested ranging from 1 to 10 positive identifications measuring single pesticide concentrations approaching 10 ppm.

## Supplementary Information

Below is the link to the electronic supplementary material.

Supplementary file1 (DOCX 6746 KB)

## Data Availability

Data available by request from the authors.
