# Supplementary material for: Quantitative determination and validation of 96 pesticides in cannabis by LC-MS/MS and GC-MS/MS
Source: Anal Bioanal Chem. 2025 Jun 11;417(17):3959–73. doi: 10.1007/s00216-025-05918-9 (PMC12227361; doi:10.1007/s00216-025-05918-9)
Supplement: Supplementary file 1 — Supplementary file1 (DOCX 6746 KB) [file 216_2025_5918_MOESM1_ESM.docx]

Quantitative Determination and Validation of 96 Pesticides in Cannabis by LC-MS/MS and GC-MS/MS

MacKenzie, D.A.*^1^, Anyanwu, A.M.^2^, McRae, G.^1^, and Melanson, J.E.^1^

^1^ National Research Council of Canada, Metrology, 1200 Montreal Road, Ottawa, ON, K1A 0R6, Canada

^2^ Environment and Climate Change Canada, Air Quality Research Division, 335 River Road, Ottawa, ON, K1V 1C7, Canada

* Corresponding Author

E-mail Address: [Douglas.MacKenzie@nrc-cnrc.gc.ca](mailto:Douglas.MacKenzie@nrc-cnrc.gc.ca)

Supplementary Information

Contents

[Supplementary Tables 3](#_Toc169004000)

[Table S 1 - Global Settings for TSQ Altis 3](#_Toc169004001)

[Table S 2 - Vanquish Flex LC-MS/MS Gradient Parameters 3](#_Toc169004002)

[Table S 3 - TSQ 9000 Global Settings 3](#_Toc169004003)

[Table S 4 - GC-MS/MS PTV Inlet Parameters 3](#_Toc169004004)

[Table S 5 - GC-MS/MS Oven Temperature Program 4](#_Toc169004005)

[Table S 6 - LC-MS/MS Transitions (Quantification Transitions Identified in Bold) 4](#_Toc169004006)

[Table S 7 - GC-MS/MS Transitions (Quantification Transitions Identified in Bold) 9](#_Toc169004007)

[Table S 8 - Master Calibration Solution Preparation 10](#_Toc169004008)

[Table S 9 - Spiking Solution Preparation 11](#_Toc169004009)

[Table S 10 - Internal Standard Spiking Solution 11](#_Toc169004010)

[Table S 11 - Spiking Volumes Used 11](#_Toc169004011)

[Table S 12 - LC-MS/MS Between Sample Accuracy at 25 ppb (Individually Calibrated Analytes) 12](#_Toc169004012)

[Table S 13 - LC-MS/MS Between-Sample Accuracy at 25 ppb (Group Calibrated Analytes) 14](#_Toc169004013)

[Table S 14 - LC-MS/MS Between-Sample Accuracy at 250 ppb (Individually Calibrated Analytes) 14](#_Toc169004014)

[Table S 15 - LC-MS/MS Between Sample Accuracy at 250 ppb (Group Calibrated Analytes) 16](#_Toc169004015)

[Table S 16 - GC-MS/MS Between-Sample Accuracy at 25 ppb (Individually Calibrated Analytes) 17](#_Toc169004016)

[Table S 17 – GC-MS/MS Between-Sample Accuracy at 25 ppb (Group Calibrated Analytes) 17](#_Toc169004017)

[Table S 18 - GC-MS/MS Between-Sample Accuracy at 75 ppb (Individually Calibrated Analytes) 17](#_Toc169004018)

[Table S 19 - GC-MS/MS Between-Sample Accuracy at 75 ppb (Group Calibrated Analytes) 18](#_Toc169004019)

[Table S 20 - GC-MS/MS Between-Sample Accuracy at 1250 ppb (Individually Calibrated Analytes) 18](#_Toc169004020)

[Table S 21 - GC-MS/MS Between-Sample Accuracy at 1250 ppb (Group Calibrated Analytes) 18](#_Toc169004021)

[Table S 22 - LC-MS/MS Analyte Recovery (Individually Calibrated Analytes) 19](#_Toc169004022)

[Table S 23 - LC-MS/MS Ion Suppression 21](#_Toc169004023)

[Supplementary Figures 25](#_Toc169004024)

[Figure S 2 - LC-MS/MS Calibration Curves (1 of 8) 26](#_Toc169004025)

[Figure S 3 - LC-MS/MS Calibration Curves (2 of 8) 27](#_Toc169004026)

[Figure S 4 - LC-MS/MS Calibration Curves (3 of 8) 28](#_Toc169004027)

[Figure S 5 - LC-MS/MS Calibration Curves (4 of 8) 29](#_Toc169004028)

[Figure S 6 - LC-MS/MS Calibration Curve (5 of 8) 30](#_Toc169004029)

[Figure S 7 - LC-MS/MS Calibration Curves (6 of 8) 31](#_Toc169004030)

[Figure S 8 - LC-MS/MS Calibration Curves (7 of 8) 32](#_Toc169004031)

[Figure S 9 - LC-MS/MS Calibration Curves (8 of 8) 33](#_Toc169004032)

[Figure S 10 - GC-MS/MS Calibration Curves (1 of 3) 34](#_Toc169004033)

[Figure S 11 - GC-MS/MS Calibration Curves (2 of 3) 35](#_Toc169004034)

[Figure S 12 - GC-MS/MS Calibration Curves (3 of 3) 36](#_Toc169004035)

Figure S 13 - LC-MS/MS Selected Ion Chromatograms at 75 ppb (1 of 9)

Figure S 14 - LC-MS/MS Selected Ion Chromatograms at 75 ppb (2 of 9)

Figure S 15 - LC-MS/MS Selected Ion Chromatograms at 75 ppb (3 of 9)

Figure S 16 - LC-MS/MS Selected Ion Chromatograms at 75 ppb (4 of 9)

Figure S 17 - LC-MS/MS Selected Ion Chromatograms at 75 ppb (5 of 9)

Figure S 18 - LC-MS/MS Selected Ion Chromatograms at 75 ppb (6 of 9)

Figure S 19 - LC-MS/MS Selected Ion Chromatograms at 75 ppb (7 of 9)

Figure S 20 - LC-MS/MS Selected Ion Chromatograms at 75 ppb (8 of 9)

Figure S 21 - LC-MS/MS Selected Ion Chromatograms at 75 ppb (9 of 9)

Figure S 22 - GC-MS/MS Selected Ion Chromatograms at 75 ppb (1 of 3). Note: Kinoprene and Methoprene, highlighted with a bold outline, are displayed at 1250 ppb.

Figure S 23 - GC-MS/MS Selected Ion Chromatograms at 75 ppb (2 of 3). Note: cis and trans permethrins, highlighted with a bold outline, are displayed at 1250 ppb.

Figure S 24 - GC-MS/MS Selected Ion Chromatograms at 75 ppb. Note: Cypermethrins 1-4, highlighted with a bold border, are displayed at 250 ppb.

Figure S 25 - Effect of THCA and dilution on peak shape of dodemorph (RT = 4.9 min).

[*Figure S 26 - LC-MS/MS Chromatogram of Dodemorph, Spiroxamine, and Cyprodinil spiked at 75 ppb on matrix* 50](#_Toc169004049)

# Supplementary Tables

## Table S 1 - Global Settings for TSQ Altis

| Parameter | MS/MS Setting |
| --- | --- |
| Scan Type | MRM |
| Ion Source | H-ESI |
| Probe Position | 2.0 and M |
| Polarity | Positive / Negative |
| ISV (V) | 4000 / 3000 |
| Sheath Gas (arb) | 70 |
| Aux Gas (arb) | 25 |
| Sweep Gas (arb) | 2 |
| Transfer Tube Temperature (°C) | 310 |
| Vaporizer Temperature (°C) | 150 |
| RF Lens | Analyte Specific |
| Collision Gas (mTorr) | 2 |
| Cycle Time (s) | 0.4 |

## Table S 2 - Vanquish Flex LC-MS/MS Gradient Parameters

| Time (min) | Flow (mL/min) | %A | %B |
| --- | --- | --- | --- |
| 0 | 0.5 | 80 | 20 |
| 5.5 | 0.5 | 25 | 75 |
| 10.5 | 0.5 | 2 | 98 |
| 13 | 0.5 | 2 | 98 |
| 13.1 | 0.5 | 80 | 20 |
| 16 | 0.5 | 80 | 20 |

## Table S 3 - TSQ 9000 Global Settings

| Parameter | Setting |
| --- | --- |
| Method Type | Acquisition - Timed |
| MS Transfer Line Temperature (°C) | 325 |
| Ion Source Temperature (°C) | 330 |
| Ionization Mode | EI |

## Table S 4 - GC-MS/MS PTV Inlet Parameters and Temperature Settings

| PTV Step | Rate (°C/s) | Inlet Temperature (°C) | Time (min) | Flow (mL/min) |
| --- | --- | --- | --- | --- |
| Injection | - | 100 | 0.05 | 50 |
| Evap | 2.5 | 120 | 0.5 |  |
| Transfer | 2.5 | 325 | 1 |  |
| Cleaning | 14.5 | 340 | 20 | 100 |
|  |  |  |  |  |
| Temperature Settings | | | | |
| Enable Temperature Control | Yes |  |  |  |
| Temperature | 100 °C |  |  |  |
|  |  |  |  |  |
| Inlet Parameters | | | | |
| Operating mode | Splitless |  |  |  |
| Split flow control | Yes |  |  |  |
| Split flow | 60.0 mL/min |  |  |  |
| Split Ratio | 50 |  |  |  |
| Splitless Time | 1.00 min |  |  |  |
| Purge flow control | Yes |  |  |  |
| Purge flow | 20.000 mL/min |  |  |  |
| Constant septum purge | Yes |  |  |  |
| Vacuum compensation | Yes |  |  |  |
| Enable gas saver mode | Yes |  |  |  |
| Gas saver flow | 20.0 mL/min |  |  |  |
| Gas saver time | 5.00 min |  |  |  |

## Table S 5 - GC-MS/MS Oven Temperature Program

| Retention Time (min) | Rate (°C/min) | Target Value (°C) | Hold Time (min) |
| --- | --- | --- | --- |
| 1.5 | 0 | 40 | 1.5 |
| 7.1 | 25 | 180 | 0 |
| 18.975 | 8 | 275 | 0 |
| 30.308 | 15 | 325 | 8 |

Table S 6 - LC-MS/MS Transitions (Quantification Transitions Identified in Bold)

| Analyte | RT (min) | RT Window (min) | Polarity | Precursor (m/z) | Product (m/z) | CE (V) | RF Lens (V) |
| --- | --- | --- | --- | --- | --- | --- | --- |
| Daminozide | 0.61 | 0.5 | + | **161.09** | **44** | 21 | 36 |
| Daminozide | 0.61 | 0.5 | + | 161.09 | 142.917 | 10 | 36 |
| Daminozide-d4 | 0.61 | 0.5 | + | 165.112 | 44 | 20 | 36 |
| Daminozide-d4 | 0.61 | 0.5 | + | 165.112 | 45.083 | 22 | 36 |
| Daminozide-d4 | 0.61 | 0.5 | + | 165.112 | 62.073 | 13 | 36 |
| Daminozide-d4 | 0.61 | 0.5 | + | **165.112** | **147.083** | 12 | 36 |
| Acephate | 0.91 | 0.5 | + | 183.95 | 124.8 | 18 | 30 |
| Acephate | 0.91 | 0.5 | + | **183.95** | **142.97** | 9 | 30 |
| Dinotefuran | 1.4 | 0.5 | + | **203.047** | **113.071** | 10.23 | 30 |
| Dinotefuran | 1.4 | 0.5 | + | 203.047 | 129.071 | 11.63 | 30 |
| Oxamyl (+NH4) | 1.42 | 0.5 | + | **237.1** | **72.125** | 12 | 30 |
| Oxamyl (+NH4) | 1.42 | 0.5 | + | 237.1 | 90.125 | 9 | 30 |
| Flonicamid | 2 | 1 | + | 230.1 | 174.04 | 17 | 41 |
| Flonicamid | 2 | 1 | + | **230.1** | **203.11** | 16 | 41 |
| Methomyl | 1.81 | 0.5 | + | **162.85** | **88.042** | 9 | 30 |
| Methomyl | 1.81 | 0.5 | + | 162.85 | 106.113 | 10 | 30 |
| Thiamethoxam | 2.13 | 1 | + | 292.03 | 181.04 | 23 | 52 |
| Thiamethoxam | 2.13 | 1 | + | **292.03** | **211.1** | 12 | 52 |
| Thiamethoxam-d4 | 2.13 | 1 | + | 296.175 | 183 | 23 | 54 |
| Thiamethoxam-d4 | 2.13 | 1 | + | 296.175 | 214.083 | 11 | 54 |
| Thiamethoxam-d4 | 2.13 | 1 | + | **296.175** | **215.083** | 13 | 54 |
| Mevinphos Iso 1 | 2.9 | 2 | + | **224.988** | **126.988** | 10.23 | 31 |
| Mevinphos Iso 1 | 2.9 | 2 | + | 224.988 | 192.97 | 8 | 31 |
| Pirimicarb | 2.7 | 1 | + | **239.088** | **72.125** | 21.49 | 47 |
| Pirimicarb | 2.7 | 1 | + | 239.088 | 182.071 | 16.07 | 47 |
| Imidacloprid | 2.8 | 1 | + | 256.1 | 175.125 | 19 | 41 |
| Imidacloprid | 2.8 | 1 | + | **256.1** | **209.113** | 16 | 41 |
| Dimethoate | 3.08 | 1 | + | 230 | 124.887 | 21 | 30 |
| Dimethoate | 3.08 | 1 | + | **230** | **199.03** | 11 | 30 |
| Clothianidin | 3.26 | 1 | + | 249.974 | 112.93 | 27 | 34 |
| Clothianidin | 3.26 | 1 | + | **249.974** | **131.917** | 16.56 | 34 |
| Acetamiprid | 3.27 | 1 | + | 223.1 | 55.889 | 16.32 | 47 |
| Acetamiprid | 3.27 | 1 | + | **223.1** | **125.92** | 20 | 47 |
| Aldicarb (+NH4) | 3.5 | 1 | + | 208.1 | 89.04 | 17 | 68 |
| Aldicarb (+NH4) | 3.5 | 1 | + | **208.1** | **116.04** | 10 | 68 |
| Imazalil | 4.5 | 2 | + | **297.1** | **159.042** | 22 | 64 |
| Imazalil | 4.5 | 2 | + | 297.1 | 255.071 | 18.24 | 64 |
| Thiacloprid | 4.03 | 1 | + | 253.1 | 90.1 | 36 | 56 |
| Thiacloprid | 4.03 | 1 | + | **253.1** | **125.91** | 21 | 56 |
| Dichlorvos | 4.17 | 1 | + | **220.866** | **109.042** | 17.77 | 86 |
| Dichlorvos | 4.17 | 1 | + | 220.866 | 145.042 | 13.87 | 86 |
| Propoxur | 4.2 | 1 | + | **210.1** | **111.04** | 14 | 30 |
| Propoxur | 4.2 | 1 | + | 210.1 | 168.11 | 8 | 30 |
| Carbofuran | 4.3 | 1 | + | 222.1 | 122.988 | 21 | 32 |
| Carbofuran | 4.3 | 1 | + | **222.1** | **165.125** | 12 | 32 |
| Dodemorph | 5 | 2 | + | 282.225 | 98.13 | 26.53 | 52 |
| Dodemorph | 5 | 2 | + | **282.225** | **116.1** | 20.92 | 52 |
| Imazalil-d5 | 4.5 | 1 | + | **302.2** | **159** | 24 | 71 |
| Imazalil-d5 | 4.5 | 1 | + | 302.2 | 203 | 19 | 71 |
| Imazalil-d5 | 4.5 | 1 | + | 302.2 | 255.083 | 19 | 71 |
| Thiophanate Methyl | 4.8 | 1 | + | **342.962** | **151.04** | 19.82 | 47 |
| Thiophanate Methyl | 4.8 | 1 | + | 342.962 | 268 | 10.25 | 47 |
| Metalaxyl | 4.9 | 1 | + | 280.1 | 192.14 | 20 | 50 |
| Metalaxyl | 4.9 | 1 | + | **280.1** | **220.13** | 15 | 50 |
| Carbaryl | 5.05 | 1 | + | 202.1 | 126.97 | 28 | 30 |
| Carbaryl | 5.05 | 1 | + | **202.1** | **145.042** | 11 | 30 |
| Azadirachtin | 5.05 | 1 | + | **703.222** | **585.2** | 13.79 | 77 |
| Azadirachtin | 5.05 | 1 | + | 703.222 | 685.2 | 10.23 | 77 |
| Cyantraniliprole | 5.24 | 1 | + | **474.95** | **285.905** | 14.25 | 70 |
| Cyantraniliprole | 5.24 | 1 | + | 474.95 | 443.93 | 18.83 | 70 |
| Spiroxamine | 5.8 | 2 | + | **298.3** | **100.2** | 30 | 48 |
| Spiroxamine | 5.8 | 2 | + | 298.3 | 144.2 | 20 | 48 |
| Fensulfothion | 5.3 | 1 | + | 308.962 | 235 | 22.44 | 58 |
| Fensulfothion | 5.3 | 1 | + | **308.962** | **280.935** | 14.93 | 58 |
| Naled | 5.4 | 1 | + | **380.8** | **126.97** | 18 | 72 |
| Naled | 5.4 | 1 | + | 382.8 | 126.97 | 15 | 72 |
| Dimethomorph | 6 | 2 | + | 388 | 165.2 | 35 | 76 |
| Dimethomorph | 6 | 2 | + | **388** | **301** | 25 | 76 |
| Azoxystrobin | 5.8 | 1 | + | 404.1 | 344.111 | 25.12 | 49 |
| Azoxystrobin | 5.8 | 1 | + | **404.1** | **372.1** | 13 | 49 |
| Chlorantraniliprole | 5.86 | 1 | + | **481.9** | **283.9** | 13 | 54 |
| Chlorantraniliprole | 5.86 | 1 | + | 481.9 | 450.9 | 17 | 54 |
| Phosmet | 6.04 | 1 | + | **318** | **160** | 10 | 77 |
| Phosmet | 6.04 | 1 | + | 335 | 160 | 20 | 77 |
| Methiocarb | 6.1 | 1 | + | 226.1 | 121.042 | 19 | 36 |
| Methiocarb | 6.1 | 1 | + | **226.1** | **169.054** | 10 | 36 |
| Ethoprophos | 6.1 | 1 | + | 243.1 | 96.917 | 32.44 | 45 |
| Ethoprophos | 6.1 | 1 | + | **243.1** | **130.9** | 20 | 45 |
| Iprodione | 6.1 | 1 | + | 330.125 | 216.125 | 34.61 | 103 |
| Iprodione | 6.1 | 1 | + | **330.125** | **298.054** | 22.23 | 103 |
| Malathion | 6.1 | 1 | + | **330.95** | **126.99** | 12 | 63 |
| Malathion | 6.1 | 1 | + | 330.95 | 284.92 | 10.23 | 63 |
| Spirotetramat | 6.1 | 1 | + | **374.2** | **216.08** | 34.41 | 55 |
| Spirotetramat | 6.1 | 1 | + | 374.2 | 330.165 | 15.56 | 55 |
| Paclobutrazol | 6.17 | 1 | + | 294.1 | 70.12 | 22 | 52 |
| Paclobutrazol | 6.17 | 1 | + | **294.1** | **124.97** | 33 | 52 |
| Cyprodinil | 6.69 | 2 | + | **226.1** | **93.1** | 33 | 86 |
| Cyprodinil | 6.69 | 2 | + | 226.1 | 108.054 | 26.79 | 86 |
| Myclobutanil | 6.28 | 1 | + | **289.1** | **70** | 18 | 62 |
| Myclobutanil | 6.28 | 1 | + | 289.1 | 125 | 31 | 62 |
| Myclobutanil-d4 | 6.28 | 1 | + | **293.1** | **70** | 18 | 62 |
| Myclobutanil-d4 | 6.28 | 1 | + | 293.1 | 129 | 31 | 62 |
| Boscalid | 6.29 | 1 | + | **343.05** | **272.111** | 31.34 | 71 |
| Boscalid | 6.29 | 1 | + | 343.05 | 307.1 | 19 | 71 |
| Propiconazole | 6.8 | 2 | + | 342 | 69 | 20 | 64 |
| Propiconazole | 6.8 | 2 | + | **342** | **159.03** | 29 | 64 |
| Bifenazate | 6.4 | 1 | + | 301.2 | 170.12 | 21 | 34 |
| Bifenazate | 6.4 | 1 | + | **301.2** | **198.13** | 8 | 34 |
| Fluopyram | 6.4 | 1 | + | 397.012 | 145.02 | 51.21 | 70 |
| Fluopyram | 6.4 | 1 | + | **397.012** | **208.04** | 21.79 | 70 |
| Spinosyn A | 7 | 2 | + | 732.42 | 98.155 | 45 | 94 |
| Spinosyn A | 7 | 2 | + | **732.42** | **142.125** | 29 | 94 |
| Tetrachlorvinphos | 6.6 | 1 | + | **364.95** | **127.042** | 12.77 | 73 |
| Tetrachlorvinphos | 6.6 | 1 | + | 364.95 | 203.804 | 38.17 | 73 |
| Kresoxim Methyl | 6.65 | 1 | + | 314.09 | 235.071 | 16.22 | 30 |
| Kresoxim Methyl | 6.65 | 1 | + | **314.09** | **267.13** | 7 | 30 |
| Kresoxim Methyl | 6.65 | 1 | + | 331.3 | 235.071 | 16 | 31 |
| Kresoxim Methyl (+NH4) | 6.65 | 1 | + | 331.3 | 267.13 | 7 | 31 |
| Kresoxim Methyl (+NH4) | 6.65 | 1 | + | 331.3 | 314.09 | 5 | 31 |
| Kresoxim-Methyl-d7(+NH4) | 6.65 | 1 | + | 338.338 | 116 | 19 | 31 |
| Kresoxim-Methyl-d7(+NH4) | 6.65 | 1 | + | 338.338 | 206.083 | 10 | 31 |
| Kresoxim-Methyl-d7(+NH4) | 6.65 | 1 | + | **338.338** | **321.25** | 5 | 31 |
| Tebufenozide | 6.7 | 1 | + | **353.15** | **133.071** | 19.29 | 30 |
| Tebufenozide | 6.7 | 1 | + | 353.15 | 297.15 | 10.23 | 30 |
| Spinetoram L | 7.2 | 2 | + | 760.462 | 98.083 | 47 | 116 |
| Spinetoram L | 7.2 | 2 | + | **760.462** | **142.125** | 29.9 | 116 |
| Fenoxycarb | 6.8 | 1 | + | 302.1 | 88.113 | 18 | 46 |
| Fenoxycarb | 6.8 | 1 | + | **302.1** | **116.042** | 11 | 46 |
| Diazinon | 6.8 | 1 | + | 305.1 | 153.1 | 21 | 75 |
| Diazinon | 6.8 | 1 | + | **305.1** | **169.1** | 20 | 75 |
| Diazinon-d10 | 6.8 | 1 | + | 315.235 | 98.917 | 36 | 75 |
| Diazinon-d10 | 6.8 | 1 | + | 315.235 | 154.083 | 22 | 75 |
| Diazinon-d10 | 6.8 | 1 | + | **315.235** | **170.083** | 22 | 75 |
| Tetramethrin Iso 1 | 7.4 | 2 | + | 332.188 | 135.083 | 18.19 | 33 |
| Tetramethrin Iso 1 | 7.4 | 2 | + | **332.188** | **164.054** | 23.61 | 33 |
| Tebuconazole | 7 | 1 | + | **308.2** | **70.1** | 24 | 55 |
| Tebuconazole | 7 | 1 | + | 308.2 | 151 | 25 | 55 |
| Spinosyn D | 7.5 | 2 | + | 746.446 | 98.125 | 44.92 | 104 |
| Spinosyn D | 7.5 | 2 | + | **746.446** | **142.155** | 29.68 | 104 |
| Spinetoram J | 7.5 | 2 | + | 748.499 | 98.071 | 42.91 | 114 |
| Spinetoram J | 7.5 | 2 | + | **748.499** | **142.125** | 29.03 | 114 |
| Fludioxonil | 7 | 1 | - | **247** | **126.14** | 32 | 89 |
| Fludioxonil | 7 | 1 | - | 247 | 180.05 | 28 | 89 |
| Fenthion | 7.1 | 1 | + | **279** | **169** | 18.4 | 55 |
| Fenthion | 7.1 | 1 | + | 279 | 246.9 | 19 | 55 |
| Prallethrin | 7.2 | 1 | + | 301.18 | 123.125 | 14.74 | 75 |
| Prallethrin | 7.2 | 1 | + | **301.18** | **151.125** | 10.23 | 75 |
| Pyraclostrobin | 7.2 | 1 | + | 388.068 | 163.054 | 24.03 | 42 |
| Pyraclostrobin | 7.2 | 1 | + | **388.068** | **194.06** | 12.27 | 42 |
| Benzovindiflupyr | 7.2 | 1 | + | 398.063 | 342.125 | 17.39 | 77 |
| Benzovindiflupyr | 7.2 | 1 | + | **398.063** | **378.042** | 13.75 | 77 |
| Fipronil | 7.2 | 1 | - | 434.9 | 249.958 | 26 | 77 |
| Fipronil | 7.2 | 1 | - | **434.9** | **329.958** | 15 | 77 |
| Pyrethrin II | 7.33 | 1 | + | 373.2 | 143.083 | 19 | 53 |
| Pyrethrin II | 7.33 | 1 | + | **373.2** | **161.083** | 12 | 53 |
| Trifloxystrobin | 7.35 | 1 | + | **409.09** | **186.07** | 17 | 60 |
| Trifloxystrobin | 7.35 | 1 | + | 409.09 | 206.13 | 14 | 60 |
| Coumaphos | 7.4 | 1 | + | **362.912** | **226.97** | 25.85 | 61 |
| Coumaphos | 7.4 | 1 | + | 362.912 | 334.988 | 16.79 | 61 |
| Clofentezine | 7.6 | 1 | + | 303.09 | 102.04 | 35 | 84 |
| Clofentezine | 7.6 | 1 | + | **303.09** | **138.04** | 13 | 84 |
| Buprofezin | 7.8 | 1 | + | **306.098** | **115.97** | 16.14 | 37 |
| Buprofezin | 7.8 | 1 | + | 306.098 | 201.08 | 12.05 | 37 |
| Piperonyl Butoxide (+NH4) | 7.8 | 1 | + | 356.2 | 119.1 | 33 | 32 |
| Piperonyl Butoxide (+NH4) | 7.8 | 1 | + | **356.2** | **177.1** | 12 | 32 |
| Piperonylbutoxide-d9 | 7.8 | 1 | + | 365.315 | 91 | 52 | 32 |
| Piperonylbutoxide-d9 | 7.8 | 1 | + | 365.315 | 119.083 | 36 | 32 |
| Piperonylbutoxide-d9 | 7.8 | 1 | + | **365.315** | **177.083** | 13 | 32 |
| Allethrin | 7.9 | 1 | + | 303.208 | 123.07 | 17.01 | 30 |
| Allethrin | 7.9 | 1 | + | **303.208** | **135** | 10.23 | 30 |
| Etoxazole | 8.2 | 1 | + | **360.2** | **140.97** | 29 | 66 |
| Etoxazole | 8.2 | 1 | + | 360.2 | 177.04 | 19.51 | 66 |
| Novaluron | 8.2 | 1 | + | 493.02 | 140.95 | 44.73 | 78 |
| Novaluron | 8.2 | 1 | + | **493.02** | **157.97** | 19.21 | 78 |
| Hexythiazox | 8.3 | 1 | + | 353.1 | 168.1 | 25 | 43 |
| Hexythiazox | 8.3 | 1 | + | **353.1** | **227.99** | 16 | 43 |
| Pyrethrin I | 8.33 | 1 | + | **329.2** | **143.083** | 17 | 47 |
| Pyrethrin I | 8.33 | 1 | + | 329.2 | 161.083 | 11 | 47 |
| Spiromesifen (+NH4) | 8.4 | 1 | + | 371.125 | 273.183 | 10.25 | 30 |
| Spiromesifen (+NH4) | 8.4 | 1 | + | **388.023** | **273.083** | 15.31 | 30 |
| Resmethrin | 9 | 2 | + | **339.112** | **171.071** | 14.97 | 70 |
| Resmethrin | 9 | 2 | + | 339.112 | 293.083 | 13.72 | 70 |
| Fenpyroximate | 8.52 | 1 | + | 422.2 | 215.111 | 25.42 | 60 |
| Fenpyroximate | 8.52 | 1 | + | **422.2** | **366.1** | 16 | 60 |
| Chlorpyrifos | 8.6 | 1 | + | 349.9 | 197.9 | 19 | 49 |
| Chlorpyrifos | 8.6 | 1 | + | 349.9 | 321.9 | 12 | 49 |
| Spirodiclofen | 8.61 | 1 | + | 411.074 | 71.083 | 16.56 | 69 |
| Spirodiclofen | 8.61 | 1 | + | **411.074** | **312.97** | 11.18 | 69 |
| Abamectin B1a (+NH4) | 8.9 | 1 | + | **890.526** | **305.238** | 25 | 61 |
| Abamectin B1a (+NH4) | 8.9 | 1 | + | 890.526 | 567.304 | 14 | 61 |
| Pyridaben | 9 | 1 | + | **365.1** | **147.13** | 25 | 64 |
| Pyridaben | 9 | 1 | + | 365.1 | 309.07 | 12 | 64 |
| Teflubenzuron | 9.1 | 1 | - | **379.025** | **195.917** | 22 | 55 |
| Teflubenzuron | 9.1 | 1 | - | 379.025 | 338.917 | 11 | 55 |
| Teflubenzuron | 9.1 | 1 | - | 379.025 | 358.917 | 5 | 55 |
| Methoprene | 9.2 | 1 | + | **279.29** | **191.17** | 8 | 35 |
| Methoprene | 9.2 | 1 | + | 311.07 | 191.083 | 12.92 | 30 |
| Fenvalerate | 9.28 | 1 | + | 437.32 | 125 | 39.5 | 52 |
| Fenvalerate | 9.28 | 1 | + | **437.32** | **167.2** | 17 | 52 |
| Deltamethrin (+NH4) | 9.5 | 1 | + | **522.95** | **280.905** | 16.33 | 69 |
| Deltamethrin (+NH4) | 9.5 | 1 | + | 522.95 | 505.84 | 10.23 | 69 |
| Deltamethrin-d5 | 9.5 | 1 | + | 528.142 | 185.083 | 39 | 69 |
| Deltamethrin-d5 | 9.5 | 1 | + | **528.142** | **280.917** | 17 | 69 |
| Deltamethrin-d5 | 9.5 | 1 | + | 528.142 | 511 | 11 | 69 |
| Permethrin (Both) (+NH4) | 9.7 | 1 | + | **408.09** | **183.054** | 19 | 62 |
| Permethrin (Both) (+NH4) | 9.7 | 1 | + | 408.09 | 355 | 8 | 62 |
| trans-Permethrin-d6 | 9.7 | 1 | + | 414.3 | 153.083 | 45 | 53 |
| trans-Permethrin-d6 | 9.7 | 1 | + | 414.3 | 168.083 | 42 | 53 |
| trans-Permethrin-d6 | 9.7 | 1 | + | **414.3** | **183.083** | 18 | 53 |
| Phenothrin | 9.75 | 1 | + | 351.212 | 183.083 | 20 | 51 |
| Phenothrin | 9.75 | 1 | + | **351.212** | **249.083** | 19 | 51 |
| Phenothrin | 9.75 | 1 | + | 351.212 | 305.167 | 13 | 51 |
| Etofenprox (+NH4) | 9.9 | 1 | + | 394.1 | 106.982 | 39.83 | 36 |
| Etofenprox (+NH4) | 9.9 | 1 | + | **394.1** | **177.13** | 13 | 36 |
| Bifenthrin (+NH4) | 9.92 | 1 | + | 440.2 | 166.1 | 39 | 75 |
| Bifenthrin (+NH4) | 9.92 | 1 | + | **440.2** | **181.1** | 12 | 75 |
| Acequinocyl (+NH4) | 11.07 | 1 | + | 343.2 | 189 | 22 | 91 |
| Acequinocyl (+NH4) | 11.07 | 1 | + | 402.3 | 189 | 28 | 40 |
| Acequinocyl (+NH4) | 11.07 | 1 | + | **402.3** | **343.2** | 15 | 40 |
|  |  |  |  |  |  |  |  |
|  |  |  |  |  |  |  |  |

## Table S 7 - GC-MS/MS Transitions (Quantification Transitions Identified in Bold)

| Analyte | RT (min) | RT Window (min) | Ion Polarity | Mass | Product Mass | CE (V) |
| --- | --- | --- | --- | --- | --- | --- |
| Etridiazole | 8.02 | 1 | + | 183 | 140 | 12 |
| Etridiazole | 8.02 | 1 | + | **211** | **183** | 12 |
| Quintozene | 10.38 | 1 | + | **141.9** | **106.9** | 30 |
| Quintozene | 10.38 | 1 | + | 248.8 | 213.9 | 20 |
| Quintozene-13C6 | 10.4 | 1 | + | 148 | 113 | 25 |
| Quintozene-13C6 | 10.4 | 1 | + | 219.9 | 185 | 10 |
| Quintozene-13C6 | 10.4 | 1 | + | 254.9 | 219.9 | 10 |
| Quintozene-13C6 | 10.4 | 1 | + | **300.9** | **241.8** | 15 |
| Diazinon-d10 | 10.43 | 1 | + | 138.1 | 85 | 10 |
| Diazinon-d10 | 10.43 | 1 | + | **153.1** | **138.1** | 5 |
| Diazinon | 10.49 | 1 | + | **137** | **84** | 10 |
| Diazinon | 10.49 | 1 | + | 304 | 179 | 10 |
| Methyl Parathion | 11.54 | 1 | + | 125 | 47 | 10 |
| Methyl Parathion | 11.54 | 1 | + | **263** | **109** | 10 |
| Chlorpyrifos | 12.28 | 1 | + | **197** | **169** | 14 |
| Chlorpyrifos | 12.28 | 1 | + | 314 | 258 | 12 |
| Kinoprene | 12.29 | 1 | + | 149.1 | 77 | 15 |
| Kinoprene | 12.29 | 1 | + | **149.1** | **103.1** | 5 |
| Fenthion | 12.35 | 1 | + | 278 | 109 | 18 |
| Fenthion | 12.35 | 1 | + | **278** | **169** | 17 |
| MGK-264 I | 12.75 | 1 | + | **164.1** | **98** | 14 |
| MGK-264 I | 12.75 | 1 | + | 210 | 98 | 10 |
| MGK-264 II | 12.99 | 1 | + | **164.1** | **98** | 14 |
| MGK-264 II | 12.99 | 1 | + | 210 | 98 | 10 |
| Endosulfan alpha | 13.9 | 1 | + | 195 | 159 | 6 |
| Endosulfan alpha | 13.9 | 1 | + | **240.9** | **206** | 10 |
| Methyl Kresoxim | 14.47 | 1 | + | 116 | 89 | 15 |
| Methyl Kresoxim | 14.47 | 1 | + | **206.1** | **116.1** | 10 |
| Chlorfenapyr | 14.67 | 1 | + | 59 | 31 | 5 |
| Chlorfenapyr | 14.67 | 1 | + | **247** | **227** | 10 |
| Endosulfan beta | 15.17 | 1 | + | **207** | **172** | 15 |
| Endosulfan beta | 15.17 | 1 | + | 241 | 206 | 12 |
| Endosulfan sulfate | 15.98 | 1 | + | 239 | 204 | 12 |
| Endosulfan sulfate | 15.98 | 1 | + | **273.84** | **238.93** | 10 |
| Permethrin, cis- | 19.22 | 2 | + | 127 | 91 | 10 |
| Permethrin, cis- | 19.22 | 2 | + | **163** | **127** | 5 |
| Permethrin, trans- | 19.37 | 2 | + | **163** | **127** | 5 |
| Permethrin, trans- | 19.37 | 2 | + | 183 | 153 | 10 |
| trans-Permethrin-d6 | 19.4 | 2 | + | **169.1** | **96.1** | 10 |
| trans-Permethrin-d6 | 19.4 | 2 | + | 169.1 | 133.1 | 5 |
| trans-Permethrin-d6 | 19.4 | 2 | + | 171.1 | 96.1 | 10 |
| Cyfluthrin 1 | 19.83 | 1 | + | 163 | 127 | 6 |
| Cyfluthrin 1 | 19.83 | 1 | + | **226** | **206** | 10 |
| Cyfluthrin 2 | 19.93 | 1 | + | 163 | 127 | 6 |
| Cyfluthrin 2 | 19.93 | 1 | + | **226** | **206** | 10 |
| Cyfluthrin 3 | 20 | 1 | + | 163 | 127 | 6 |
| Cyfluthrin 3 | 20 | 1 | + | **226** | **206** | 10 |
| Cyfluthrin 4 | 20.05 | 1 | + | 163 | 127 | 6 |
| Cyfluthrin 4 | 20.05 | 1 | + | **226** | **206** | 10 |
| Cypermethrin 1 | 20.17 | 1 | + | 163 | 127 | 6 |
| Cypermethrin 1 | 20.17 | 1 | + | **165** | **129** | 6 |
| Cypermethrin 2 | 20.28 | 1 | + | 163 | 127 | 6 |
| Cypermethrin 2 | 20.28 | 1 | + | **165** | **129** | 6 |
| Cypermethrin 3 | 20.34 | 1 | + | 163 | 127 | 6 |
| Cypermethrin 3 | 20.34 | 1 | + | **165** | **129** | 6 |
| Cypermethrin 4 | 20.38 | 1 | + | 163 | 127 | 6 |
| Cypermethrin 4 | 20.38 | 1 | + | **165** | **129** | 6 |
| Etofenprox | 20.5 | 1 | + | **163** | **107** | 18 |
| Etofenprox | 20.5 | 1 | + | 163 | 135 | 10 |
| Fenvalerate I | 21.06 | 1 | + | **167** | **125** | 8 |
| Fenvalerate I | 21.06 | 1 | + | 419 | 225 | 5 |
| Fenvalerate II | 21.25 | 1 | + | **167** | **125** | 8 |
| Fenvalerate II | 21.25 | 1 | + | 419 | 225 | 5 |

## Table S 8 - Master Calibration Solution Preparation

| Stock Solution | Concentration (µg/mL) | Volume used (µL) | Concentration in mixture (µg/mL) |
| --- | --- | --- | --- |
| SPEX CAN-CAN-1 | 100 | 1000 | 8.33 |
| SPEX CAN-CAN-2 | 100 | 1000 | 8.33 |
| SPEX CAN-CAN-3 | 100 | 1000 | 8.33 |
| SPEX CAN-CAN-4 | 100 | 1000 | 8.33 |
| SPEX CAN-CAN-5 | 100 | 1000 | 8.33 |
| SPEX CAN-CAN-6 | 100 | 1000 | 8.33 |
| SPEX LCS-2650 (Naled) | 1000 | 100 | 8.33 |
| SPEX LCS-3970-ACN (Dimethomorph) | 1000 | 100 | 8.33 |
| Acetonitrile | Neat | 5800 | - |

## Table S 9 - Spiking Solution Preparation

| Spiking Solution | Stock Used | Volume of Stock (µL) | Volume of Acetonitrile (µL) | Final Spiking Solution Volume (µL) | Concentration (µg/mL) |
| --- | --- | --- | --- | --- | --- |
| Spike 1 | MCS | 10 | 990 | 1000 | 0.08 |
| Spike 2 | MCS | 20 | 980 | 1000 | 0.17 |
| Spike 3 | MCS | 40 | 960 | 1000 | 0.33 |
| Spike 4 | MCS | 100 | 900 | 1000 | 0.83 |
| Spike 5 | MCS | 200 | 800 | 1000 | 1.67 |
| Spike 6 | MCS | 400 | 600 | 1000 | 3.33 |
| Spike 7 | MCS | 500 | 500 | 1000 | 4.17 |
| Spike 8 | MCS | 8000 | 0 | 8000 | 8.33 |
| Spike 9 | MCS | 50 | 950 | 1000 | 0.42 |
| Spike 10 | MCS | 150 | 850 | 1000 | 1.25 |

## Table S 10 - Internal Standard Spiking Solution

| Dissolution | | | | Dilution | | |
| --- | --- | --- | --- | --- | --- | --- |
| Pesticide | Mass (mg) | Initial Solvent Volume (mL ACN) | Concentration (mg/mL) | Volume Used (µL) | Final Volume (mL) | Final Concentration (µg/mL) |
| Daminozide-d4 | 0.207 | 1 | 0.207 | 290 | 20.00 | 3.00 |
| Myclobutanil-d4 | 0.204 | 1 | 0.204 | 196 | 20.00 | 2.00 |
| Deltamethrin-d5 | 0.313 | 1 | 0.313 | 192 | 20.00 | 3.00 |
| Piperonylbutoxide-d9 | 0.763 | 1 | 0.763 | 52 | 20.00 | 2.00 |
| Diazinon-d10 | 0.570 | 1 | 0.57 | 70 | 20.00 | 2.00 |
| Kresoxim-Methyl-d7 | 0.169 | 1 | 0.1686 | 474 | 20.00 | 4.00 |
| Trans-permethrin-d6 | 0.405 | 1 | 0.405 | 99 | 20.00 | 2.00 |
| Thiamethoxam-d4 | 0.571 | 1 | 0.571 | 70 | 20.00 | 2.00 |
| Quintozene 13C6 | 1.000 | 1 | 1 | 60 | 20.00 | 3.00 |
| Imazalil-d5 | 0.735 | 1 | 0.735 | 109 | 20.00 | 4.00 |

## Table S 11 - Spiking Volumes Used

| Calibrant | Mass of Cannabis Used (g) | Spiking Solution Used | Analyte Spiking Volume (µL) | Internal Standard Spiking Volume (µL) |
| --- | --- | --- | --- | --- |
| 5 ppb | 1.00 | Spike 1 | 60 | 100 |
| 10 ppb | 1.00 | Spike 2 | 60 | 100 |
| 20 ppb | 1.00 | Spike 3 | 60 | 100 |
| 50 ppb | 1.00 | Spike 4 | 60 | 100 |
| 100 ppb | 1.00 | Spike 5 | 60 | 100 |
| 200 ppb | 1.00 | Spike 6 | 60 | 100 |
| 500 ppb | 1.00 | Spike 8 (MCS) | 60 | 100 |
| 1000 ppb | 1.00 | Spike 8 (MCS) | 120 | 100 |
| 1500 ppb | 1.00 | Spike 8 (MCS) | 180 | 100 |
| 2000 ppb | 1.00 | Spike 8 (MCS) | 240 | 100 |
| QC 25 | 1.00 | Spike 9 | 60 | 100 |
| QC 75 | 1.00 | Spike 10 | 60 | 100 |
| QC 250 | 1.00 | Spike 7 | 60 | 100 |
| QC 1250 | 1.00 | Spike 8 (MCS) | 150 | 100 |

## Table S 12 - LC-MS/MS Between Sample Accuracy at 25 ppb (Individually Calibrated Analytes)

| Analyte | CS-1 | CS-2 | CS-3 | CS-4 | CS-5 | CS-6 | Hemp | Cal Blend |
| --- | --- | --- | --- | --- | --- | --- | --- | --- |
| THCA (mg/g) | 127 | 5.0 | 0.4 | 146 | 272 | 47.1 | 1.0 | 57.3 |
| CBDA (mg/g) | 0.3 | 146 | 56.4 | 0.4 | 0.6 | 108 | 14.6 | 84.5 |
| Accuracy (%) | | | | | | | | |
| Daminozide | 178.8 | 119.9 | 98.0 | 145.1 | 139.5 | 141.3 | 51.1 | 99.3 |
| Acephate | 114.5 | 108.5 | 107.1 | 108.6 | 104.8 | 102.1 | 107.5 | 106.0 |
| Dinotefuran | 105.5 | 104.1 | 99.5 | 102.6 | 101.7 | 101.4 | 94.9 | 102.2 |
| Oxamyl (+NH4) | 114.1 | 104.5 | 100.5 | 102.5 | 98.1 | 101.3 | 98.3 | 98.6 |
| Methomyl | 108.5 | 102.4 | 99.3 | 105.1 | 101.3 | 96.2 | 99.1 | 103.2 |
| Flonicamid | 105.2 | 100.8 | 91.1 | 107.0 | 103.3 | 101.0 | 95.5 | 103.7 |
| Thiamethoxam | 110.2 | 105.0 | 95.2 | 99.0 | 98.5 | 96.8 | 99.3 | 99.0 |
| Pirimicarb | 109.7 | 110.2 | 95.9 | 104.7 | 116.5 | 99.0 | 91.7 | 99.0 |
| Imidacloprid | 109.3 | 98.4 | 82.4 | 94.5 | 88.6 | 90.5 | 88.4 | 96.4 |
| Dimethoate | 112.3 | 100.3 | 97.5 | 96.1 | 99.7 | 95.8 | 97.6 | 99.5 |
| Clothianidin | 100.4 | 101.8 | 95.7 | 95.7 | 96.7 | 97.2 | 81.6 | 99.5 |
| Acetamiprid | 111.7 | 103.6 | 93.0 | 99.5 | 99.6 | 97.7 | 90.0 | 100.7 |
| Aldicarb (+NH4) | 104.4 | 88.6 | 94.1 | 103.3 | 97.2 | 98.4 | 95.6 | 97.4 |
| Thiacloprid | 111.2 | 98.1 | 87.2 | 101.7 | 101.4 | 90.4 | 83.2 | 99.4 |
| Dichlorvos | 115.9 | 102.3 | 91.3 | 100.6 | 96.2 | 105.8 | 96.8 | 108.7 |
| Propoxur | 114.3 | 107.8 | 93.5 | 104.6 | 105.6 | 95.0 | 93.6 | 102.7 |
| Carbofuran | 105.6 | 106.4 | 94.0 | 100.1 | 99.9 | 95.0 | 87.7 | 99.5 |
| Imazalil | 107.7 | 91.8 | 93.3 | 102.9 | 122.0 | 99.9 | 89.2 | 100.8 |
| Dodemorph | 132.5 | 89.7 | 105.1 | 75.8 | 100.5 | 85.2 | 114.1 | 96.0 |
| Thiophanate Methyl | 128.7 | 87.6 | 67.0 | 77.3 | 39.3 | 84.4 | 121.8 | 101.0 |
| Metalaxyl | 97.8 | 102.2 | 96.1 | 95.6 | 89.6 | 99.7 | 85.4 | 101.1 |
| Azadirachtin | 109.0 | 73.4 | 100.4 | 117.8 | 42.2 | 113.9 | 104.5 | 96.0 |
| Carbaryl | 96.5 | 103.9 | 93.5 | 89.9 | 93.7 | 102.9 | 90.1 | 104.0 |
| Spiroxamine | 143.5 | 84.8 | 73.0 | 136.3 | 158.6 | 81.6 | 82.7 | 95.5 |
| Cyantraniliprole | 85.4 | 122.2 | 100.7 | 87.6 | 64.8 | 87.7 | 71.0 | 98.1 |
| Fensulfothion | 105.1 | 109.6 | 107.1 | 103.0 | 105.9 | 97.0 | 95.8 | 102.8 |
| Naled | 104.2 | 103.0 | 101.5 | 110.0 | 96.8 | 95.5 | 90.6 | 104.9 |
| Azoxystrobin | 129.7 | 100.9 | 97.4 | 112.9 | 122.3 | 99.2 | 64.8 | 108.8 |
| Chlorantraniliprole | 122.0 | 106.3 | 95.9 | 110.2 | 105.5 | 95.9 | 105.3 | 106.8 |
| Phosmet | 111.1 | 93.5 | 91.1 | 93.7 | 86.5 | 86.6 | 89.2 | 104.8 |
| Iprodione | 111.5 | 81.8 | 63.2 | 78.1 | 111.6 | 75.0 | 64.0 | 78.0 |
| Spirotetramat | 131.4 | 67.9 | 82.2 | 100.1 | 91.6 | 81.0 | 100.6 | 97.0 |
| Malathion | 100.3 | 110.6 | 101.7 | 108.8 | 106.2 | 90.5 | 93.2 | 109.6 |
| Methiocarb | 100.4 | 104.7 | 103.0 | 105.3 | 96.6 | 94.9 | 94.2 | 103.3 |
| Ethoprophos | 102.4 | 110.0 | 98.5 | 93.5 | 102.1 | 91.7 | 101.9 | 103.3 |
| Paclobutrazol | 124.7 | 100.7 | 102.6 | 114.0 | 129.0 | 102.9 | 116.9 | 101.9 |
| Myclobutanil | 116.3 | 94.6 | 100.1 | 104.9 | 100.9 | 93.3 | 96.0 | 101.3 |
| Boscalid | 113.4 | 104.3 | 115.2 | 105.2 | 112.4 | 109.9 | -229.3 | 110.0 |
| Fluopyram | 94.0 | 104.8 | 117.1 | 87.3 | 85.3 | 104.0 | 86.2 | 99.4 |
| Bifenazate | 108.2 | 108.2 | 108.6 | 95.2 | 98.8 | 100.5 | 97.2 | 103.3 |
| Tetrachlorvinphos | 117.3 | 101.2 | 98.7 | 97.5 | 100.9 | 93.9 | 123.9 | 105.0 |
| Kresoxim Methyl | 99.6 | 96.8 | 96.6 | 102.4 | 108.1 | 92.4 | 122.8 | 98.2 |
| Tebufenozide | 107.9 | 92.5 | 85.8 | 95.2 | 92.2 | 97.0 | 114.3 | 99.6 |
| Propiconazole | 132.1 | 96.1 | 92.5 | 108.9 | 118.9 | 116.1 | 108.3 | 108.3 |
| Cyprodinil | 143.8 | 76.8 | 69.1 | 141.5 | * | 87.7 | 80.9 | 97.9 |
| Diazinon | 110.1 | 103.1 | 98.2 | 93.9 | 98.8 | 101.0 | 105.6 | 102.3 |
| Fenoxycarb | 119.8 | 96.9 | 97.3 | 97.1 | 113.4 | 104.9 | 126.2 | 107.0 |
| Fludioxonil | 97.6 | 121.2 | 117.3 | 105.3 | 70.9 | 93.0 | 124.3 | 88.8 |
| Tebuconazole | 133.8 | 106.9 | 110.5 | 117.3 | 92.4 | 103.0 | 106.2 | 106.6 |
| Fenthion | 119.9 | 108.6 | 110.6 | 104.5 | 116.2 | 100.5 | 109.4 | 111.3 |
| Fipronil | 101.0 | 101.7 | 89.4 | 96.4 | 99.2 | 101.1 | 103.6 | 95.4 |
| Benzovindiflupyr | 114.8 | 95.8 | 101.8 | 104.6 | 112.8 | 88.7 | 100.0 | 100.1 |
| Prallethrin | 99.1 | 48.6 | 74.4 | 75.6 | 94.6 | 101.2 | -131.4 | 85.8 |
| Pyraclostrobin | 109.0 | 101.1 | 102.5 | 102.3 | 105.9 | 93.7 | 98.8 | 100.6 |
| Trifloxystrobin | 105.7 | 100.4 | 100.4 | 101.9 | 97.5 | 98.0 | 105.0 | 102.3 |
| Coumaphos | 111.9 | 95.2 | 89.2 | 96.5 | 107.5 | 92.2 | 73.0 | 100.3 |
| Buprofezin | 118.2 | 101.5 | 99.1 | 100.3 | 102.8 | 99.5 | 99.4 | 100.9 |
| Piperonyl Butoxide (+NH4) | 107.9 | 99.0 | 102.8 | 97.2 | 104.5 | 91.3 | 97.1 | 98.6 |
| Clofentezine | 54.3 | 62.2 | 85.4 | 27.4 | 24.1 | 66.5 | 49.0 | 94.3 |
| Allethrin | 64.3 | 60.9 | -179.0 | 48.6 | 46.0 | 92.4 | -68.6 | 74.7 |
| Novaluron | 147.4 | 104.1 | 86.6 | 94.9 | 113.9 | 87.8 | 135.8 | 95.8 |
| Spiromesifen (+NH4) | 110.8 | 113.3 | 100.5 | 99.6 | 116.2 | 104.9 | 101.5 | 99.0 |
| Etoxazole | 93.0 | 106.2 | 107.1 | 84.8 | 87.9 | 107.1 | 100.6 | 101.4 |
| Hexythiazox | 127.5 | 103.5 | 114.7 | 81.5 | 105.2 | 87.2 | 137.1 | 99.8 |
| Chlorpyrifos | 118.4 | 107.2 | 113.8 | 94.7 | 109.6 | 99.7 | 109.5 | 104.0 |
| Fenpyroximate | 87.4 | 120.0 | 87.3 | 74.7 | 117.8 | 104.2 | 89.4 | 98.8 |
| Spirodiclofen | 82.8 | 144.3 | 128.1 | 118.4 | 134.8 | 127.1 | -40.4 | 103.1 |
| Abamectin B1a (+NH4) | BLQ | BLQ | BLQ | BLQ | BLQ | BLQ | BLQ | BLQ |
| Teflubenzuron | 125.2 | 135.3 | 61.3 | 89.5 | 114.4 | 51.7 | 93.2 | 92.7 |
| Methoprene | 105.3 | 105.5 | 99.4 | 99.7 | 95.5 | 74.4 | 89.9 | 101.2 |
| Pyridaben | 107.4 | 101.4 | 102.0 | 98.5 | 103.7 | 103.0 | 114.4 | 101.4 |
| Deltamethrin (+NH4) | 111.8 | 98.6 | 89.3 | 98.5 | 86.5 | 82.2 | 82.6 | 94.9 |
| Fenvalerate | 145.1 | 73.3 | 142.6 | 167.2 | 130.4 | 100.1 | 21.0 | 85.8 |
| trans-Permethrin (+NH4) | 51.1 | 108.0 | 109.2 | 72.6 | 63.1 | 98.5 | 96.0 | 96.0 |
| Phenothrin | 114.9 | 133.8 | 115.7 | 105.8 | 138.5 | 97.0 | 168.2 | 97.4 |
| Etofenprox (+NH4) | -62.8 | 128.2 | 18.9 | -141.0 | -134.7 | 124.4 | -99.9 | 94.8 |
| Bifenthrin (+NH4) | 97.6 | 128.7 | 103.5 | 97.8 | 133.0 | 98.7 | 56.6 | 106.8 |
| Acequinocyl (+NH4) | 22.8 | 115.1 | 115.6 | 105.7 | 112.3 | 104.5 | 123.1 | 85.5 |
| * Cyprodinil gives a poor peak shape in high THCA samples such that it is indistinguishable from noise at 25 ppb in this sample. Dilution (10x in 20:80 MP-A:MP-B) restores peak shape. | | | | | | | | |

## Table S 13 - LC-MS/MS Between-Sample Accuracy at 25 ppb (Group Calibrated Analytes)

| Analyte | CS-1 | CS-2 | CS-3 | CS-4 | CS-5 | CS-6 | Hemp | Cal Blend |
| --- | --- | --- | --- | --- | --- | --- | --- | --- |
| THCA (mg/g) | 127 | 5.0 | 0.4 | 146 | 272 | 47.1 | 1.0 | 57.3 |
| CBDA (mg/g) | 0.3 | 146 | 56.4 | 0.4 | 0.6 | 108 | 14.6 | 84.5 |
| Accuracy (%) | | | | | | | | |
| Dimethomorph Group | 130.5 | 91.5 | 90.5 | 104.1 | 113.3 | 91.5 | 92.1 | 103.1 |
| Mevinphos Group | 109.6 | 101.2 | 98.5 | 103.6 | 103.6 | 96.5 | 88.8 | 96.0 |
| Pyrethrins | 192.9 | 91.5 | 169.3 | 148.4 | 161.5 | 110.7 | -27.1 | 147.0 |
| Resmethrin Group | 41.6 | 76.3 | 45.8 | 35.2 | 46.3 | 49.5 | 31.5 | 85.7 |
| Spinetoram Group | 119.1 | 105.1 | 102.1 | 107.0 | 104.1 | 94.8 | 169.2 | 86.9 |
| Spinosad Group | 113.5 | 108.2 | 109.6 | 105.5 | 81.2 | 82.5 | 162.9 | 86.0 |
| Tetramethrin Group | 96.1 | 83.1 | -13.6 | 81.9 | 112.9 | 84.4 | -26.8 | 93.8 |

## Table S 14 - LC-MS/MS Between-Sample Accuracy at 250 ppb (Individually Calibrated Analytes)

| Analyte | CS-1 | CS-2 | CS-3 | CS-4 | CS-5 | CS-6 | Hemp | Cal Blend |
| --- | --- | --- | --- | --- | --- | --- | --- | --- |
| THCA (mg/g) | 127 | 5.0 | 0.4 | 146 | 272 | 47.1 | 1.0 | 57.3 |
| CBDA (mg/g) | 0.3 | 146 | 56.4 | 0.4 | 0.6 | 108 | 14.6 | 84.5 |
| Accuracy (%) | | | | | | | | |
| Daminozide | 102.7 | 90.8 | 90.9 | 86.8 | 104.1 | 104.9 | 74.8 | 98.3 |
| Acephate | 103.1 | 106.0 | 107.3 | 108.6 | 104.0 | 107.4 | 105.0 | 105.9 |
| Dinotefuran | 99.3 | 101.4 | 101.3 | 99.8 | 99.4 | 107.9 | 96.3 | 103.0 |
| Oxamyl (+NH4) | 101.1 | 100.5 | 102.7 | 99.1 | 98.8 | 107.9 | 97.8 | 102.6 |
| Methomyl | 99.9 | 99.0 | 104.0 | 96.6 | 103.8 | 105.1 | 100.3 | 103.8 |
| Flonicamid | 90.4 | 94.0 | 92.1 | 100.4 | 99.9 | 107.7 | 91.4 | 102.6 |
| Thiamethoxam | 97.5 | 96.8 | 95.5 | 98.2 | 100.4 | 102.5 | 95.3 | 101.6 |
| Pirimicarb | 91.8 | 102.5 | 90.8 | 107.0 | 112.2 | 106.5 | 91.7 | 100.8 |
| Imidacloprid | 105.7 | 93.5 | 92.1 | 99.5 | 101.0 | 108.1 | 90.0 | 103.9 |
| Dimethoate | 98.0 | 97.4 | 100.4 | 97.3 | 99.6 | 102.8 | 97.7 | 103.2 |
| Clothianidin | 94.6 | 97.1 | 97.0 | 96.1 | 104.9 | 109.6 | 90.3 | 103.1 |
| Acetamiprid | 100.8 | 96.5 | 94.4 | 97.3 | 103.3 | 107.5 | 87.7 | 103.4 |
| Aldicarb (+NH4) | 101.7 | 99.0 | 102.4 | 99.6 | 108.1 | 107.1 | 98.8 | 102.3 |
| Thiacloprid | 100.9 | 92.9 | 90.0 | 100.9 | 106.9 | 97.5 | 84.0 | 97.9 |
| Dichlorvos | 102.0 | 99.8 | 99.2 | 101.3 | 107.0 | 106.7 | 103.5 | 104.3 |
| Propoxur | 102.3 | 100.1 | 98.1 | 100.8 | 107.3 | 104.3 | 93.9 | 102.8 |
| Carbofuran | 95.7 | 100.0 | 96.0 | 95.0 | 98.5 | 102.3 | 89.9 | 99.4 |
| Imazalil | 90.1 | 87.8 | 93.9 | 74.6 | 92.2 | 97.0 | 93.9 | 99.0 |
| Dodemorph | 137.9 | 82.3 | 111.7 | 107.7 | 103.2 | 103.9 | 93.2 | 94.4 |
| Thiophanate Methyl | 127.6 | 70.7 | 67.9 | 96.6 | 44.2 | 96.3 | 122.8 | 102.8 |
| Metalaxyl | 88.3 | 100.0 | 99.4 | 100.5 | 95.8 | 98.8 | 83.9 | 97.4 |
| Azadirachtin | 77.6 | 104.4 | 103.2 | 95.2 | 39.7 | 100.4 | 81.2 | 98.8 |
| Carbaryl | 77.3 | 105.9 | 98.4 | 93.5 | 87.2 | 98.7 | 85.0 | 99.7 |
| Spiroxamine | 146.1 | 68.5 | 93.6 | 169.1 | 162.4 | 108.3 | 46.4 | 93.7 |
| Cyantraniliprole | 77.8 | 116.6 | 98.8 | 90.0 | 78.6 | 98.5 | 68.1 | 99.5 |
| Fensulfothion | 90.5 | 104.1 | 112.0 | 103.2 | 96.7 | 102.1 | 92.6 | 99.5 |
| Naled | 89.1 | 103.3 | 95.1 | 105.8 | 86.8 | 95.9 | 84.0 | 101.0 |
| Azoxystrobin | 104.0 | 102.7 | 99.5 | 105.0 | 112.8 | 96.6 | 81.0 | 99.4 |
| Chlorantraniliprole | 108.4 | 103.4 | 99.1 | 100.7 | 101.1 | 98.2 | 98.3 | 102.6 |
| Phosmet | 96.1 | 91.7 | 90.6 | 92.8 | 87.4 | 95.4 | 84.5 | 101.9 |
| Iprodione | 126.7 | 100.3 | 98.1 | 110.3 | 126.4 | 98.0 | 85.1 | 103.0 |
| Spirotetramat | 115.6 | 92.1 | 93.7 | 103.7 | 93.5 | 92.7 | 87.5 | 99.6 |
| Malathion | 92.5 | 106.4 | 111.7 | 103.0 | 99.6 | 103.7 | 94.5 | 102.8 |
| Methiocarb | 91.6 | 104.1 | 107.0 | 101.0 | 93.2 | 102.6 | 91.7 | 100.4 |
| Ethoprophos | 95.7 | 103.4 | 109.9 | 102.3 | 106.6 | 101.8 | 95.6 | 99.6 |
| Paclobutrazol | 111.8 | 94.9 | 100.9 | 115.7 | 95.0 | 100.8 | 102.3 | 98.6 |
| Myclobutanil | 96.2 | 99.6 | 102.4 | 96.5 | 101.9 | 102.1 | 96.3 | 100.6 |
| Boscalid | 90.8 | 99.8 | 103.2 | 101.2 | 98.7 | 96.5 | 60.3 | 102.6 |
| Fluopyram | 81.0 | 104.0 | 119.2 | 89.8 | 88.2 | 111.4 | 87.0 | 99.6 |
| Bifenazate | 90.9 | 100.3 | 115.1 | 94.7 | 95.4 | 107.8 | 93.9 | 99.5 |
| Tetrachlorvinphos | 101.5 | 97.1 | 103.5 | 94.1 | 94.6 | 101.4 | 132.4 | 103.9 |
| Kresoxim Methyl | 98.0 | 97.9 | 106.4 | 94.7 | 93.6 | 106.2 | 133.3 | 103.4 |
| Tebufenozide | 93.1 | 98.9 | 107.5 | 101.7 | 89.8 | 106.9 | 136.6 | 101.1 |
| Propiconazole | 116.8 | 93.4 | 102.0 | 111.9 | 113.9 | 112.8 | 98.8 | 105.3 |
| Cyprodinil | 127.7 | 86.1 | 74.8 | 136.5 | 122.2 | 108.9 | 69.7 | 101.0 |
| Diazinon | 101.0 | 98.0 | 99.5 | 96.0 | 98.6 | 106.3 | 94.5 | 98.3 |
| Fenoxycarb | 107.9 | 101.5 | 104.5 | 102.8 | 106.7 | 105.8 | 138.6 | 105.7 |
| Fludioxonil | 81.2 | 108.3 | 118.9 | 81.2 | 92.7 | 103.5 | 126.1 | 107.3 |
| Tebuconazole | 127.1 | 102.6 | 111.4 | 111.5 | 129.5 | 105.9 | 111.0 | 107.2 |
| Fenthion | 109.5 | 103.6 | 112.1 | 102.8 | 110.5 | 105.6 | 101.6 | 106.7 |
| Fipronil | 94.7 | 110.1 | 104.9 | 107.9 | 99.5 | 112.0 | 101.2 | 100.7 |
| Benzovindiflupyr | 110.8 | 100.2 | 112.1 | 105.0 | 118.5 | 104.8 | 99.8 | 102.5 |
| Prallethrin | 107.3 | 99.6 | 104.5 | 102.0 | 120.0 | 99.5 | 77.9 | 98.9 |
| Pyraclostrobin | 104.9 | 101.5 | 108.2 | 102.5 | 107.9 | 106.6 | 101.2 | 100.4 |
| Trifloxystrobin | 99.4 | 99.0 | 101.6 | 101.5 | 96.1 | 102.8 | 104.4 | 102.3 |
| Coumaphos | 116.4 | 92.3 | 99.1 | 104.3 | 111.7 | 102.2 | 93.6 | 103.1 |
| Buprofezin | 107.8 | 99.3 | 102.4 | 103.8 | 107.4 | 106.8 | 96.0 | 104.4 |
| Piperonyl Butoxide (+NH4) | 105.9 | 103.8 | 109.1 | 105.7 | 106.5 | 104.5 | 94.2 | 102.4 |
| Clofentezine | 74.0 | 91.2 | 117.1 | 48.1 | 41.0 | 96.9 | 79.2 | 101.0 |
| Allethrin | 50.9 | 98.1 | 89.5 | 29.8 | 28.5 | 99.1 | 40.9 | 101.6 |
| Novaluron | 151.7 | 93.0 | 91.3 | 88.5 | 122.2 | 94.5 | 153.0 | 100.6 |
| Spiromesifen (+NH4) | 98.5 | 104.8 | 104.3 | 99.2 | 115.2 | 110.8 | 96.9 | 101.1 |
| Etoxazole | 101.1 | 103.0 | 105.2 | 97.8 | 108.4 | 107.5 | 99.7 | 100.3 |
| Hexythiazox | 126.7 | 102.7 | 121.8 | 91.6 | 114.5 | 98.1 | 140.1 | 103.1 |
| Chlorpyrifos | 107.6 | 103.5 | 115.5 | 99.2 | 112.4 | 107.1 | 114.0 | 103.9 |
| Fenpyroximate | 82.6 | 112.7 | 91.9 | 80.3 | 119.2 | 115.1 | 83.1 | 100.2 |
| Spirodiclofen | 92.1 | 115.9 | 98.4 | 97.2 | 128.0 | 112.9 | 86.9 | 97.8 |
| Abamectin B1a (+NH4) | 72.2 | 98.5 | 120.0 | 70.8 | 70.2 | 112.9 | 96.0 | 86.0 |
| Teflubenzuron | 111.5 | 122.8 | 75.8 | 97.6 | 112.4 | 106.1 | 113.5 | 95.7 |
| Methoprene | 103.7 | 101.4 | 103.9 | 98.9 | 99.9 | 105.9 | 107.0 | 98.4 |
| Pyridaben | 102.5 | 100.6 | 103.7 | 99.4 | 101.3 | 106.0 | 108.3 | 101.1 |
| Deltamethrin (+NH4) | 102.3 | 98.8 | 103.1 | 97.7 | 100.7 | 104.3 | 102.5 | 97.7 |
| Fenvalerate | 110.4 | 103.9 | 106.0 | 99.9 | 101.3 | 102.0 | 103.2 | 99.6 |
| trans-Permethrin (+NH4) | 85.6 | 102.3 | 105.5 | 101.1 | 99.8 | 108.1 | 98.5 | 102.4 |
| Phenothrin | 115.9 | 102.3 | 93.6 | 109.6 | 126.5 | 102.0 | 115.4 | 100.9 |
| Etofenprox (+NH4) | -77.3 | 126.6 | 12.5 | -145.8 | -124.3 | 127.8 | -113.2 | 101.4 |
| Bifenthrin (+NH4) | 85.8 | 122.0 | 103.2 | 95.1 | 136.3 | 109.0 | 56.4 | 100.1 |
| Acequinocyl (+NH4) | 100.7 | 93.2 | 112.0 | 103.8 | 104.2 | 103.0 | 113.6 | 98.3 |

## Table S 15 - LC-MS/MS Between Sample Accuracy at 250 ppb (Group Calibrated Analytes)

| Analyte | CS-1 | CS-2 | CS-3 | CS-4 | CS-5 | CS-6 | Hemp | Cal Blend |
| --- | --- | --- | --- | --- | --- | --- | --- | --- |
| THCA (mg/g) | 127 | 5.0 | 0.4 | 146 | 272 | 47.1 | 1.0 | 57.3 |
| CBDA (mg/g) | 0.3 | 146 | 56.4 | 0.4 | 0.6 | 108 | 14.6 | 84.5 |
| Accuracy (%) | | | | | | | | |
| Dimethomorph Group | 124.9 | 96.3 | 99.6 | 112.4 | 113.6 | 96.5 | 90.7 | 102.3 |
| Mevinphos Group | 97.6 | 95.6 | 102.0 | 95.3 | 100.1 | 99.5 | 95.4 | 98.9 |
| Pyrethrins | 103.4 | 107.2 | 116.9 | 106.1 | 107.8 | 101.1 | 107.7 | 101.0 |
| Resmethrin Group | 65.7 | 100.6 | 103.0 | 76.4 | 84.2 | 109.2 | 66.1 | 101.0 |
| Spinetoram Group | 141.1 | 81.3 | 136.2 | 172.7 | 105.4 | 124.2 | 128.6 | 97.7 |
| Spinosad Group | 123.4 | 87.6 | 129.7 | 165.2 | 90.3 | 125.5 | 125.5 | 97.2 |
| Tetramethrin Group | 98.8 | 93.2 | 93.1 | 87.5 | 93.8 | 103.7 | 82.8 | 102.6 |

## Table S 16 - GC-MS/MS Between-Sample Accuracy at 25 ppb (Individually Calibrated Analytes)

| Analyte | CS-1 | CS-6 | CS-7 | CS-8 | CS-9 | CS-10 | Hemp | Cal Blend |
| --- | --- | --- | --- | --- | --- | --- | --- | --- |
| THCA (mg/g) | 127 | 47.1 | 3.4 | 37 | 177 | 44.5 | 1.0 | 57.3 |
| CBDA (mg/g) | 0.3 | 108 | 135 | 102 | 0.4 | 87.5 | 14.6 | 84.5 |
| Accuracy (%) | | | | | | | | |
| Etridiazole | 105.0 | 91.6 | 99.1 | 99.4 | 100.5 | 99.4 | 128.1 | 95.3 |
| Quintozene | 81.4 | 98.0 | 87.8 | 94.6 | 99.5 | 94.6 | 108.5 | 91.2 |
| Diazinon | 87.2 | 97.9 | 87.0 | 86.0 | 85.5 | 86.0 | 105.3 | 90.9 |
| Methyl Parathion | 93.4 | 94.9 | 103.8 | 88.0 | 104.5 | 88.0 | 121.3 | 93.5 |
| Chlorpyrifos | 77.4 | 108.2 | 99.3 | 107.4 | 122.5 | 107.4 | -69.5 | 90.1 |
| Kinoprene | BLQ | BLQ | BLQ | BLQ | BLQ | BLQ | BLQ | BLQ |
| Fenthion | 73.5 | 90.5 | 97.5 | 94.6 | 79.2 | 94.6 | 108.5 | 92.0 |
| Endosulfan alpha | 84.9 | 57.4 | 107.6 | 23.3 | 63.4 | 23.3 | 103.5 | 91.7 |
| Chlorfenapyr | BLQ | BLQ | BLQ | BLQ | BLQ | BLQ | BLQ | BLQ |
| Endosulfan beta | 65.5 | 87.5 | 136.3 | 122.0 | 107.9 | 122.0 | 76.9 | 90.3 |
| Endosulfan Sulfate | 87.0 | 97.8 | 90.3 | 92.8 | 96.3 | 92.8 | 86.9 | 93.9 |
| Etofenprox | BLQ | BLQ | BLQ | BLQ | BLQ | BLQ | BLQ | BLQ |

## Table S 17 – GC-MS/MS Between-Sample Accuracy at 25 ppb (Group Calibrated Analytes)

| Analyte | CS-1 | CS-6 | CS-7 | CS-8 | CS-9 | CS-10 | Hemp | Cal Blend |
| --- | --- | --- | --- | --- | --- | --- | --- | --- |
| THCA (mg/g) | 127 | 47.1 | 3.4 | 37 | 177 | 44.5 | 1.0 | 57.3 |
| CBDA (mg/g) | 0.3 | 108 | 135 | 102 | 0.4 | 87.5 | 14.6 | 84.5 |
| Accuracy (%) | | | | | | | | |
| Cyfluthrin group | BLQ | BLQ | BLQ | BLQ | BLQ | BLQ | BLQ | BLQ |
| Cypermethrin group | BLQ | BLQ | BLQ | BLQ | BLQ | BLQ | BLQ | BLQ |
| Fenvalerate group | BLQ | BLQ | BLQ | BLQ | BLQ | BLQ | BLQ | BLQ |
| MGK group | 90.2 | 109.5 | 111.9 | 103.3 | 85.8 | 103.3 | 101.4 | 95.8 |
| Permethrin group | BLQ | BLQ | BLQ | BLQ | BLQ | BLQ | BLQ | BLQ |

## Table S 18 - GC-MS/MS Between-Sample Accuracy at 75 ppb (Individually Calibrated Analytes)

| Analyte | CS-1 | CS-6 | CS-7 | CS-8 | CS-9 | CS-10 | Hemp | Cal Blend |
| --- | --- | --- | --- | --- | --- | --- | --- | --- |
| THCA (mg/g) | 127 | 47.1 | 3.4 | 37 | 177 | 44.5 | 1.0 | 57.3 |
| CBDA (mg/g) | 0.3 | 108 | 135 | 102 | 0.4 | 87.5 | 14.6 | 84.5 |
| Accuracy (%) | | | | | | | | |
| Etridiazole | 98.1 | 92.9 | 104.1 | 93.3 | 93.9 | 93.3 | 106.5 | 96.1 |
| Quintozene | 91.4 | 88.3 | 97.3 | 102.9 | 88.6 | 102.9 | 104.8 | 96.8 |
| Diazinon | 88.6 | 97.2 | 94.3 | 99.5 | 89.6 | 99.5 | 93.1 | 95.0 |
| Methyl Parathion | 108.4 | 106.2 | 113.4 | 101.6 | 104.9 | 101.6 | 103.2 | 98.3 |
| Chlorpyrifos | 96.9 | 103.7 | 103.2 | 95.7 | 100.9 | 95.7 | 79.6 | 96.1 |
| Kinoprene | BLQ | BLQ | BLQ | BLQ | BLQ | BLQ | BLQ | BLQ |
| Fenthion | 91.6 | 97.7 | 112.8 | 90.4 | 85.8 | 90.4 | 99.7 | 96.6 |
| Endosulfan alpha | 104.3 | 84.6 | 111.0 | 80.8 | 89.7 | 80.8 | 89.2 | 96.1 |
| Chlorfenapyr | 106.4 | 82.3 | 99.6 | 98.9 | 109.1 | 98.9 | 87.9 | 94.5 |
| Endosulfan beta | 92.8 | 98.8 | 105.0 | 87.6 | 97.1 | 87.6 | 77.4 | 91.3 |
| Endosulfan Sulfate | 94.5 | 100.9 | 97.5 | 96.7 | 107.2 | 96.7 | 77.6 | 97.1 |
| Etofenprox | 110.6 | 112.8 | 113.9 | 103.0 | 106.4 | 103.0 | 87.1 | 91.0 |

## Table S 19 - GC-MS/MS Between-Sample Accuracy at 75 ppb (Group Calibrated Analytes)

| Analyte | CS-1 | CS-6 | CS-7 | CS-8 | CS-9 | CS-10 | Hemp | Cal Blend |
| --- | --- | --- | --- | --- | --- | --- | --- | --- |
| THCA (mg/g) | 127 | 47.1 | 3.4 | 37 | 177 | 44.5 | 1.0 | 57.3 |
| CBDA (mg/g) | 0.3 | 108 | 135 | 102 | 0.4 | 87.5 | 14.6 | 84.5 |
| Accuracy (%) | | | | | | | | |
| Cyfluthrin group | 107.0 | 99.4 | 107.5 | 97.8 | 112.1 | 97.8 | 70.8 | 94.8 |
| Cypermethrin group | BLQ | BLQ | BLQ | BLQ | BLQ | BLQ | BLQ | BLQ |
| Fenvalerate group | 108.8 | 109.9 | 119.8 | 94.2 | 99.7 | 94.2 | 76.2 | 97.2 |
| MGK group | 91.7 | 102.9 | 103.9 | 104.3 | 97.5 | 104.3 | 90.4 | 95.8 |
| Permethrin group | BLQ | BLQ | BLQ | BLQ | BLQ | BLQ | BLQ | BLQ |

## Table S 20 - GC-MS/MS Between-Sample Accuracy at 1250 ppb (Individually Calibrated Analytes)

| Analyte | CS-1 | CS-6 | CS-7 | CS-8 | CS-9 | CS-10 | Hemp | Cal Blend |
| --- | --- | --- | --- | --- | --- | --- | --- | --- |
| THCA (mg/g) | 127 | 47.1 | 3.4 | 37 | 177 | 44.5 | 1.0 | 57.3 |
| CBDA (mg/g) | 0.3 | 108 | 135 | 102 | 0.4 | 87.5 | 14.6 | 84.5 |
| Accuracy (%) | | | | | | | | |
| Etridiazole | 93.3 | 90.2 | 92.2 | 97.9 | 93.5 | 97.9 | 96.8 | 95.5 |
| Quintozene | 94.4 | 95.6 | 97.1 | 101.5 | 99.5 | 101.5 | 87.5 | 96.5 |
| Diazinon | 91.8 | 93.1 | 95.0 | 102.4 | 99.1 | 102.4 | 83.6 | 96.5 |
| Methyl Parathion | 101.7 | 101.1 | 104.1 | 106.3 | 98.8 | 106.3 | 101.5 | 97.6 |
| Chlorpyrifos | 94.2 | 92.0 | 94.8 | 102.6 | 99.3 | 102.6 | 112.5 | 95.6 |
| Kinoprene | 92.2 | 100.6 | 95.0 | 112.9 | 113.5 | 112.9 | 93.1 | 97.1 |
| Fenthion | 88.3 | 88.5 | 96.7 | 100.6 | 92.8 | 100.6 | 96.0 | 98.3 |
| Endosulfan alpha | 91.0 | 91.0 | 97.8 | 102.1 | 101.3 | 102.1 | 83.2 | 97.6 |
| Chlorfenapyr | 105.8 | 90.0 | 96.2 | 103.3 | 108.1 | 103.3 | 80.1 | 97.1 |
| Endosulfan beta | 97.6 | 93.0 | 92.8 | 103.1 | 102.7 | 103.1 | 76.8 | 97.2 |
| Endosulfan Sulfate | 114.2 | 105.0 | 92.0 | 103.1 | 102.0 | 103.1 | 76.3 | 95.5 |
| Etofenprox | 96.7 | 97.6 | 97.1 | 98.6 | 93.8 | 98.6 | 80.8 | 95.9 |

## Table S 21 - GC-MS/MS Between-Sample Accuracy at 1250 ppb (Group Calibrated Analytes)

| Analyte | CS-1 | CS-6 | CS-7 | CS-8 | CS-9 | CS-10 | Hemp | Cal Blend |
| --- | --- | --- | --- | --- | --- | --- | --- | --- |
| THCA (mg/g) | 127 | 47.1 | 3.4 | 37 | 177 | 44.5 | 1.0 | 57.3 |
| CBDA (mg/g) | 0.3 | 108 | 135 | 102 | 0.4 | 87.5 | 14.6 | 84.5 |
| Accuracy (%) | | | | | | | | |
| Cyfluthrin group | 146.0 | 126.6 | 94.4 | 101.2 | 95.8 | 101.2 | 76.0 | 93.9 |
| Cypermethrin group | 140.6 | 123.9 | 102.4 | 99.8 | 95.2 | 99.8 | 68.3 | 92.8 |
| Fenvalerate group | 127.6 | 115.1 | 100.6 | 98.6 | 88.9 | 98.6 | 73.1 | 95.2 |
| MGK group | 93.5 | 98.8 | 96.9 | 107.8 | 103.2 | 107.8 | 83.3 | 99.4 |
| Permethrin group | 103.5 | 95.8 | 100.7 | 100.2 | 91.3 | 100.2 | 87.6 | 95.9 |

## Table S 22 - LC-MS/MS Analyte Recovery (Individually Calibrated Analytes)

|  | | Recovery (%) | | | | |
| --- | --- | --- | --- | --- | --- | --- |
| Analyte | RT (min) | | 25 ppb | 75 ppb | 250 ppb | Mean |
| Daminozide | 0.624 | | 74.5 | 55.4 | 43.5 | 49.4 |
| Acephate | 0.86 | | 90.2 | 86.7 | 84.0 | 87.0 |
| Dinotefuran | 1.33 | | 89.0 | 94.7 | 91.8 | 91.8 |
| Oxamyl (+NH4) | 1.342 | | 95.5 | 99.3 | 95.0 | 96.6 |
| Methomyl | 1.74 | | 95.6 | 104.3 | 101.4 | 100.4 |
| Flonicamid | 1.98 | | 98.2 | 104.1 | 98.1 | 100.1 |
| Thiamethoxam | 2.13 | | 89.1 | 95.4 | 97.3 | 93.9 |
| Mevinphos Iso 1 | 2.53 | | 100.5 | 90.5 | 95.4 | 95.5 |
| Pirimicarb | 2.77 | | 87.8 | 90.3 | 83.4 | 87.1 |
| Imidacloprid | 2.88 | | 94.9 | 99.1 | 97.6 | 97.2 |
| Dimethoate | 3.1 | | 93.0 | 101.3 | 99.7 | 98.0 |
| Mevinphos Iso 2 | 3.2 | | 93.7 | 102.6 | 95.5 | 97.3 |
| Clothianidin | 3.22 | | 90.3 | 97.3 | 95.3 | 94.3 |
| Acetamiprid | 3.3 | | 88.4 | 94.7 | 92.2 | 91.8 |
| Aldicarb (+NH4) | 3.55 | | 80.8 | 93.9 | 95.2 | 89.9 |
| Thiacloprid | 4.03 | | 93.2 | 103.0 | 96.6 | 97.6 |
| Dichlorvos | 4.2 | | 95.1 | 97.9 | 93.9 | 95.6 |
| Propoxur | 4.233 | | 94.5 | 112.3 | 97.3 | 101.4 |
| Carbofuran | 4.3 | | 94.8 | 103.8 | 94.9 | 97.8 |
| Imazalil | 4.35 | | 65.3 | 73.4 | 72.3 | 70.3 |
| Dodemorph | 4.65 | | 61.7 | 61.1 | 52.9 | 58.5 |
| Thiophanate Methyl | 4.71 | | 87.9 | 85.6 | 76.2 | 83.2 |
| Metalaxyl | 4.875 | | 94.6 | 94.0 | 87.8 | 92.1 |
| Azadirachtin | 5.02 | | 111.8 | 99.1 | 100.8 | 103.9 |
| Carbaryl | 5.06 | | 99.2 | 104.0 | 96.5 | 99.9 |
| Spiroxamine | 5.19 | | 55.9 | 62.9 | 47.6 | 55.5 |
| Cyantraniliprole | 5.212 | | 103.9 | 101.8 | 97.1 | 100.9 |
| Fensulfothion | 5.31 | | 86.5 | 96.3 | 87.2 | 90.0 |
| Naled | 5.43 | | 118.7 | 97.5 | 93.3 | 103.2 |
| Azoxystrobin | 5.78 | | 94.9 | 108.7 | 99.0 | 100.8 |
| Chlorantraniliprole | 5.86 | | 95.5 | 94.3 | 91.3 | 93.7 |
| Dimethomorph Iso 1 | 5.87 | | 77.7 | 91.5 | 86.2 | 85.2 |
| Phosmet | 6.038 | | 96.0 | 100.9 | 98.2 | 98.4 |
| Dimethomorph Iso 2 | 6.08 | | 79.6 | 85.2 | 83.1 | 82.6 |
| Iprodione | 6.09 | | 124.5 | 94.2 | 93.5 | 93.8 |
| Spirotetramat | 6.09 | | 109.7 | 99.7 | 91.3 | 100.2 |
| Malathion | 6.11 | | 107.7 | 105.3 | 99.1 | 104.0 |
| Methiocarb | 6.121 | | 93.2 | 96.3 | 95.3 | 94.9 |
| Ethoprophos | 6.129 | | 76.2 | 90.6 | 81.3 | 82.7 |
| Paclobutrazol | 6.19 | | 99.0 | 90.7 | 85.7 | 91.8 |
| Myclobutanil | 6.27 | | 86.4 | 92.2 | 88.4 | 89.0 |
| Boscalid | 6.3 | | 130.9 | 105.2 | 91.7 | 109.3 |
| Fluopyram | 6.37 | | 100.5 | 100.4 | 96.3 | 99.1 |
| Bifenazate | 6.4 | | 105.0 | 95.3 | 99.8 | 100.0 |
| Spinosyn A | 6.47 | | 47.0 | 59.7 | 44.8 | 52.2 |
| Tetrachlorvinphos | 6.6 | | 87.2 | 92.1 | 91.6 | 90.3 |
| Kresoxim Methyl | 6.69 | | 83.0 | 112.1 | 106.1 | 100.4 |
| Tebufenozide | 6.7 | | 95.9 | 103.7 | 93.1 | 97.6 |
| Spinetoram J | 6.76 | | 46.7 | 52.1 | 40.0 | 46.3 |
| Cyprodinil | 6.77 | | 43.7 | 61.0 | 31.7 | 45.4 |
| Propiconazole | 6.77 | | 73.5 | 85.2 | 71.4 | 78.3 |
| Spinosyn D | 6.78 | | 40.4 | 51.6 | 41.5 | 46.6 |
| Diazinon | 6.8 | | 90.6 | 96.6 | 90.9 | 92.7 |
| Fenoxycarb | 6.8 | | 91.6 | 97.3 | 92.4 | 93.8 |
| Fludioxonil | 6.96 | | 98.6 | 124.4 | 101.4 | 108.1 |
| Tebuconazole | 7 | | 74.4 | 82.4 | 77.2 | 78.0 |
| Spinetoram L | 7.12 | | 44.1 | 47.2 | 40.4 | 43.9 |
| Fipronil | 7.15 | | 111.6 | 103.5 | 107.4 | 107.5 |
| Fenthion | 7.16 | | 110.3 | 114.4 | 93.2 | 106.0 |
| Benzovindiflupyr | 7.2 | | 104.5 | 106.4 | 96.0 | 102.3 |
| Prallethrin | 7.21 | | 111.3 | 73.4 | 94.1 | 83.8 |
| Pyraclostrobin | 7.227 | | 96.6 | 99.9 | 101.3 | 99.3 |
| Pyrethrin II | 7.34 | | 89.2 | 99.3 | 93.0 | 96.1 |
| Trifloxystrobin | 7.36 | | 102.3 | 99.9 | 101.7 | 101.3 |
| Coumaphos | 7.37 | | 95.0 | 100.3 | 93.6 | 96.3 |
| Tetramethrin Iso 1 | 7.44 | | 108.8 | 102.1 | 95.4 | 102.1 |
| Buprofezin | 7.6 | | 106.2 | 97.8 | 86.7 | 96.9 |
| Tetramethrin Iso 2 | 7.65 | | 108.9 | 91.2 | 89.9 | 96.6 |
| Clofentezine | 7.67 | | 146.1 | 87.5 | 93.9 | 109.2 |
| Piperonyl Butoxide (+NH4) | 7.67 | | 87.6 | 97.1 | 91.7 | 92.1 |
| Allethrin | 7.71 | | 63.9 | 96.0 | 89.1 | 92.6 |
| Novaluron | 8.2 | | 102.4 | 101.4 | 96.7 | 100.2 |
| Spiromesifen (+NH4) | 8.2 | | 97.3 | 104.7 | 96.8 | 99.6 |
| Etoxazole | 8.27 | | 87.8 | 90.7 | 87.0 | 88.5 |
| Hexythiazox | 8.35 | | 91.9 | 87.5 | 88.7 | 89.3 |
| Pyrethrin I | 8.37 | | 101.4 | 143.2 | 94.9 | 119.0 |
| Chlorpyrifos | 8.42 | | 96.8 | 101.1 | 96.0 | 98.0 |
| Fenpyroximate | 8.6 | | 82.9 | 85.3 | 80.0 | 82.7 |
| Spirodiclofen | 8.67 | | 90.4 | 89.3 | 87.1 | 88.9 |
| Abamectin B1a (+NH4) | 8.89 | | 60.8 | 109.0 | 76.3 | 92.7 |
| Teflubenzuron | 8.91 | | 116.3 | 82.6 | 90.1 | 96.4 |
| Methoprene | 9.06 | | 82.1 | 78.9 | 76.5 | 79.2 |
| Pyridaben | 9.1 | | 90.0 | 90.7 | 88.8 | 89.8 |
| Resmethrin Iso 1 | 9.1 | | 69.7 | 76.5 | 86.3 | 81.4 |
| Resmethrin Iso 2 | 9.21 | | 68.0 | 85.3 | 80.6 | 82.9 |
| Deltamethrin (+NH4) | 9.29 | | 94.6 | 99.0 | 98.1 | 97.3 |
| Fenvalerate | 9.34 | | 72.5 | 141.3 | 96.0 | 118.7 |
| trans-Premethrin (+NH4) | 9.551 | | 91.8 | 102.4 | 89.9 | 96.2 |
| Phenothrin | 9.59 | | 102.2 | 100.9 | 90.8 | 98.0 |
| Etofenprox (+NH4) | 9.78 | | 87.5 | 98.1 | 85.3 | 90.3 |
| Bifenthrin (+NH4) | 10 | | 89.4 | 91.0 | 88.2 | 89.5 |
| Acequinocyl (+NH4) | 11.17 | | 160.9 | 93.2 | 80.7 | 111.6 |

## Table S 23 - LC-MS/MS Ion Suppression

|  | Ion Suppression (%) | | |
| --- | --- | --- | --- |
| Analyte | 25 ppb | 75 ppb | 250 ppb |
| Daminozide | 2.8 | -1.3 | 2.6 |
| Acephate | 1.3 | 8.0 | 8.1 |
| Dinotefuran | 3.8 | 9.7 | 5.5 |
| Oxamyl (+NH4) | -0.1 | 5.5 | 2.7 |
| Methomyl | -4.9 | 4.1 | 5.7 |
| Flonicamid | 22.5 | 27.4 | 23.2 |
| Thiamethoxam | -3.4 | 8.8 | 8.1 |
| Mevinphos Iso 1 | 18.4 | 7.1 | 7.8 |
| Pirimicarb | 19.2 | 23.0 | 18.8 |
| Imidacloprid | -4.5 | 4.5 | 2.2 |
| Dimethoate | 8.2 | 16.7 | 13.6 |
| Mevinphos Iso 2 | 1.4 | 17.5 | 6.8 |
| Clothianidin | 8.2 | 23.9 | 21.5 |
| Acetamiprid | 10.0 | 16.5 | 15.4 |
| Aldicarb (+NH4) | -17.9 | 20.1 | 7.8 |
| Thiacloprid | 13.6 | 21.7 | 15.8 |
| Dichlorvos | 22.4 | 17.6 | 11.5 |
| Propoxur | -3.1 | 16.4 | 4.1 |
| Carbofuran | 10.5 | 20.8 | 10.7 |
| Imazalil | 29.5 | 35.2 | 35.2 |
| Dodemorph | -3.0 | 8.4 | 9.4 |
| Thiophanate Methyl | 57.5 | 54.2 | 53.8 |
| Metalaxyl | 15.0 | 14.5 | 8.1 |
| Azadirachtin | 16.1 | -0.4 | 2.2 |
| Carbaryl | 13.5 | 17.3 | 15.3 |
| Spiroxamine | 25.8 | 35.1 | 29.6 |
| Cyantraniliprole | 16.2 | 25.1 | 22.5 |
| Fensulfothion | 10.6 | 14.2 | 10.9 |
| Naled | 28.0 | 22.3 | 16.7 |
| Azoxystrobin | 27.1 | 28.8 | 14.2 |
| Chlorantraniliprole | -4.2 | -0.6 | -7.7 |
| Dimethomorph Iso 1 | -15.4 | -4.8 | -7.7 |
| Phosmet | 24.9 | 30.8 | 24.0 |
| Dimethomorph Iso 2 | 5.5 | 0.2 | -2.6 |
| Iprodione | -15.4 | -23.6 | -30.0 |
| Spirotetramat | -37.3 | -18.3 | -33.2 |
| Malathion | 21.6 | 18.6 | 15.2 |
| Methiocarb | 18.2 | 21.7 | 19.1 |
| Ethoprophos | 15.1 | 19.3 | 16.1 |
| Paclobutrazol | 35.8 | 21.9 | 11.0 |
| Myclobutanil | 7.8 | 18.9 | 15.1 |
| Boscalid | 46.5 | 33.7 | 28.0 |
| Fluopyram | 27.4 | 29.1 | 30.2 |
| Bifenazate | 23.5 | 20.5 | 25.2 |
| Spinosyn A | 8.5 | 17.7 | 17.5 |
| Tetrachlorvinphos | 32.2 | 27.8 | 26.1 |
| Kresoxim Methyl | 24.1 | 23.0 | 20.6 |
| Tebufenozide | 5.6 | 17.6 | 8.6 |
| Spinetoram J | 11.5 | 17.2 | 14.0 |
| Cyprodinil | 59.8 | 55.4 | 31.8 |
| Propiconazole | 40.3 | 33.7 | 18.2 |
| Spinosyn D | 14.6 | 20.1 | 9.1 |
| Diazinon | 17.3 | 21.6 | 14.7 |
| Fenoxycarb | 23.6 | 26.7 | 25.3 |
| Fludioxonil | 7.0 | 21.1 | 13.2 |
| Tebuconazole | 9.1 | 12.2 | 8.9 |
| Spinetoram L | 5.1 | 21.4 | 14.0 |
| Fipronil | -39.5 | -28.6 | -22.8 |
| Fenthion | 40.4 | 37.0 | 29.9 |
| Benzovindiflupyr | 17.2 | 27.1 | 18.3 |
| Prallethrin | 38.6 | 11.0 | 13.6 |
| Pyraclostrobin | 19.0 | 18.8 | 17.9 |
| Pyrethrin II | -12.4 | 10.0 | 17.9 |
| Trifloxystrobin | 18.3 | 18.7 | 19.4 |
| Coumaphos | 26.4 | 24.7 | 24.7 |
| Tetramethrin Iso 1 | -19.4 | 11.6 | 12.6 |
| Buprofezin | 23.2 | 22.7 | 18.1 |
| Tetramethrin Iso 2 | 7.3 | 11.7 | 11.1 |
| Clofentezine | 84.6 | 68.2 | 59.8 |
| Piperonyl Butoxide (+NH4) | 9.3 | 14.2 | 15.5 |
| Allethrin | 48.9 | 41.9 | 44.3 |
| Novaluron | -20.0 | -20.8 | -23.0 |
| Spiromesifen (+NH4) | 18.9 | 26.9 | 24.0 |
| Etoxazole | 13.1 | 22.7 | 20.7 |
| Hexythiazox | 26.6 | 28.4 | 24.8 |
| Pyrethrin I | -937.3 | -121.9 | -23.4 |
| Chlorpyrifos | 19.7 | 23.9 | 19.6 |
| Fenpyroximate | 13.7 | 21.4 | 18.2 |
| Spirodiclofen | 24.3 | 31.5 | 25.8 |
| Abamectin B1a (+NH4) | -40.1 | 39.0 | 34.4 |
| Teflubenzuron | 9.5 | 22.2 | 29.3 |
| Methoprene | 1.6 | 13.8 | 19.8 |
| Pyridaben | 16.9 | 21.8 | 24.1 |
| Resmethrin Iso 1 | 29.0 | 23.4 | 26.1 |
| Resmethrin Iso 2 | 35.6 | 38.4 | 30.7 |
| Deltamethrin (+NH4) | 14.7 | 18.0 | 16.8 |
| Fenvalerate | 32.2 | 37.0 | 27.6 |
| trans-Permethrin (+NH4) | 31.4 | 36.2 | 32.2 |
| Phenothrin | 14.8 | 23.8 | 20.5 |
| Etofenprox (+NH4) | 77.4 | 79.2 | 76.7 |
| Bifenthrin (+NH4) | 60.3 | 65.5 | 63.2 |
| Acequinocyl (+NH4) | 15.1 | 36.7 | 36.7 |

## Table S 24 - GC-MS/MS Recovery

| Analyte | RT (min) | 25 ppb | 75 ppb | 250 ppb | 1250 ppb |
| --- | --- | --- | --- | --- | --- |
| Etridiazole | 8.05 | 85% | 96% | 94% | 88% |
| Quintozene | 10.4 | 76% | 88% | 78% | 83% |
| Diazinon | 10.52 | 92% | 85% | 83% | 82% |
| Methyl Parathion | 11.5 | 83% | 86% | 100% | 93% |
| Kinoprene | 12.3 | BLQ | BLQ | BLQ | 87% |
| Chlorpyrifos | 12.31 | 66% | 90% | 92% | 90% |
| Fenthion | 12.37 | 82% | 81% | 93% | 93% |
| MGK-264 I | 12.77 | 87% | 90% | 83% | 81% |
| MGK-264 II | 13.03 | 87% | 83% | 81% | 79% |
| Endosulfan alpha | 13.97 | 93% | 89% | 89% | 82% |
| Chlorfenapyr | 14.66 | BLQ | 88% | 98% | 93% |
| Endosulfan beta | 15.2 | 93% | 111% | 97% | 92% |
| Endosulfan Sulfate | 16 | 95% | 109% | 105% | 101% |
| Permethrin, cis- | 19.23 | BLQ | BLQ | 94% | 83% |
| Permethrin, trans- | 19.37 | BLQ | BLQ | 97% | 90% |
| Cyfluthrin 1 | 19.84 | BLQ | 147% | 116% | 116% |
| Cyfluthrin 2 | 19.95 | BLQ | 95% | 136% | 116% |
| Cyfluthrin 3 | 20 | BLQ | 139% | 121% | 134% |
| Cyfluthrin 4 | 20.06 | BLQ | 120% | 103% | 107% |
| Cypermethrin 1 | 20.18 | BLQ | BLQ | 120% | 114% |
| Cypermethrin 2 | 20.3 | BLQ | BLQ | 119% | 126% |
| Cypermethrin 3 | 20.35 | BLQ | BLQ | 130% | 120% |
| Cypermethrin 4 | 20.39 | BLQ | BLQ | 116% | 100% |
| Etofenprox | 20.5 | BLQ | 85% | 86% | 82% |
| Fenvalerate I | 21.06 | BLQ | 108% | 127% | 118% |
| Fenvalerate II | 21.25 | BLQ | 116% | 128% | 120% |

## Table S 25 - GC-MS/MS Ion Suppression

| Analyte | RT (min) | 25 ppb | 75 ppb | 250 ppb | 1250 ppb |
| --- | --- | --- | --- | --- | --- |
| Etridiazole | 8.05 | -26% | -45% | -49% | -52% |
| Quintozene | 10.4 | -88% | -92% | -113% | -94% |
| Diazinon | 10.52 | -170% | -189% | -140% | -117% |
| Methyl Parathion | 11.5 | -240% | -346% | -340% | -318% |
| Kinoprene | 12.3 | BLQ | BLQ | BLQ | -326% |
| Chlorpyrifos | 12.31 | -260% | -244% | -187% | -141% |
| Fenthion | 12.37 | -218% | -221% | -198% | -142% |
| MGK-264 I | 12.77 | -143% | -199% | -192% | -175% |
| MGK-264 II | 13.03 | -101% | -213% | -187% | -166% |
| Endosulfan alpha | 13.97 | -72% | -101% | -114% | -117% |
| Chlorfenapyr | 14.66 | BLQ | -163% | -189% | -204% |
| Endosulfan beta | 15.2 | -11% | -45% | -71% | -69% |
| Endosulfan Sulfate | 16 | -146% | -189% | -148% | -121% |
| Permethrin, cis- | 19.23 | BLQ | BLQ | -395% | -369% |
| Permethrin, trans- | 19.37 | BLQ | BLQ | -645% | -466% |
| Cyfluthrin 1 | 19.84 | BLQ | -257% | -237% | -190% |
| Cyfluthrin 2 | 19.95 | BLQ | -110% | -297% | -80% |
| Cyfluthrin 3 | 20 | BLQ | -41% | -330% | -138% |
| Cyfluthrin 4 | 20.06 | BLQ | -10% | -204% | -119% |
| Cypermethrin 1 | 20.18 | BLQ | BLQ | -203% | -133% |
| Cypermethrin 2 | 20.3 | BLQ | BLQ | -137% | -128% |
| Cypermethrin 3 | 20.35 | BLQ | BLQ | -271% | -151% |
| Cypermethrin 4 | 20.39 | BLQ | BLQ | -283% | -151% |
| Etofenprox | 20.5 | BLQ | -305% | -319% | -329% |
| Fenvalerate I | 21.06 | BLQ | #DIV/0! | -227% | -130% |
| Fenvalerate II | 21.25 | BLQ | -1015% | -571% | -280% |

# Supplementary Figures


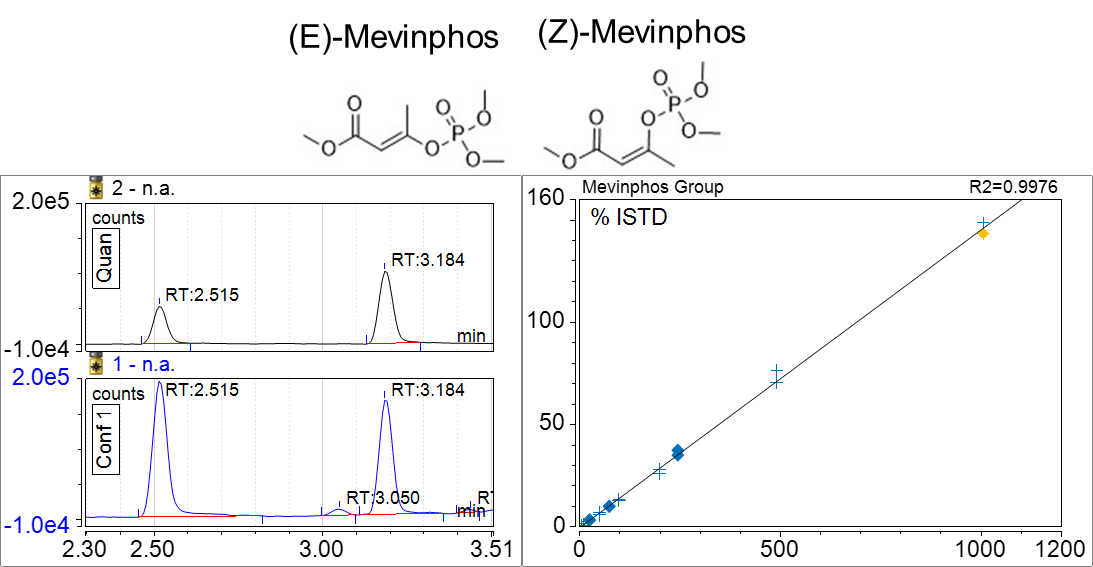
Figure S 1 - Example (Mevinphos Group) of group calibration - Two or more resolved signals contributing to a single calibration curve. Blue cross symbols indicate calibration standard injections. Blue diamonds indicate QC samples. Yellow diamond indicates the standard selected for the figure.

Figure S 2 - LC-MS/MS Calibration Curves (1 of 8). Blue cross symbols indicate calibration standard injections. Blue diamonds indicate QC samples. Yellow diamond indicates the standard selected for the figure.

## Figure S 3 - LC-MS/MS Calibration Curves (2 of 8)

## Figure S 4 - LC-MS/MS Calibration Curves (3 of 8)

## Figure S 5 - LC-MS/MS Calibration Curves (4 of 8)

## Figure S 6 - LC-MS/MS Calibration Curve (5 of 8)

## Figure S 7 - LC-MS/MS Calibration Curves (6 of 8)

## Figure S 8 - LC-MS/MS Calibration Curves (7 of 8)

## Figure S 9 - LC-MS/MS Calibration Curves (8 of 8)

Figure S 10 - GC-MS/MS Calibration Curves (1 of 3). Blue cross symbols indicate calibration standard injections. Blue diamonds indicate QC samples. Yellow diamond indicates the standard selected for the figure.

## Figure S 11 - GC-MS/MS Calibration Curves (2 of 3)

## Figure S 12 - GC-MS/MS Calibration Curves (3 of 3)


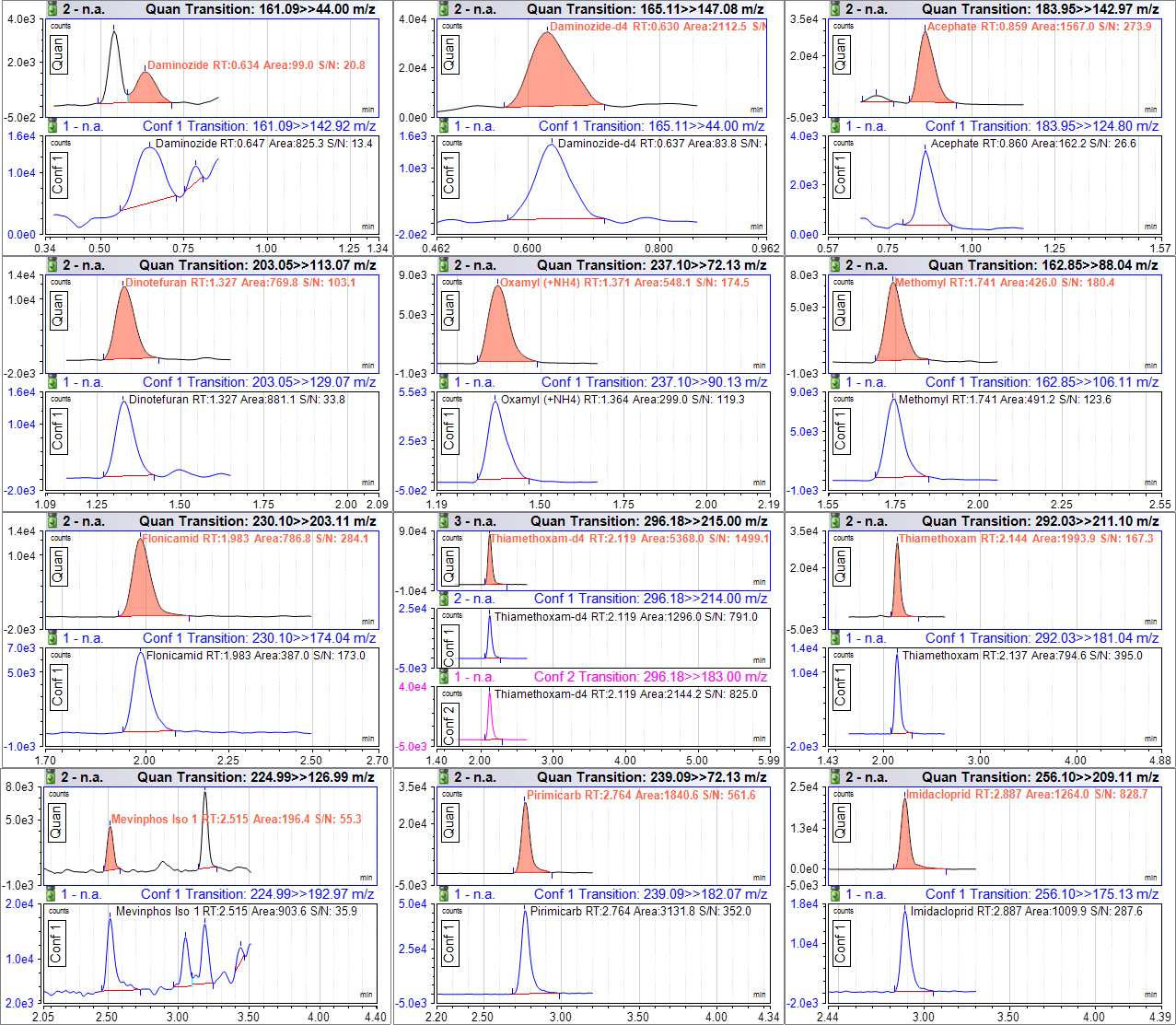


## Figure S 13 - LC-MS/MS Selected Ion Chromatograms at 75 ppb (1 of 9)


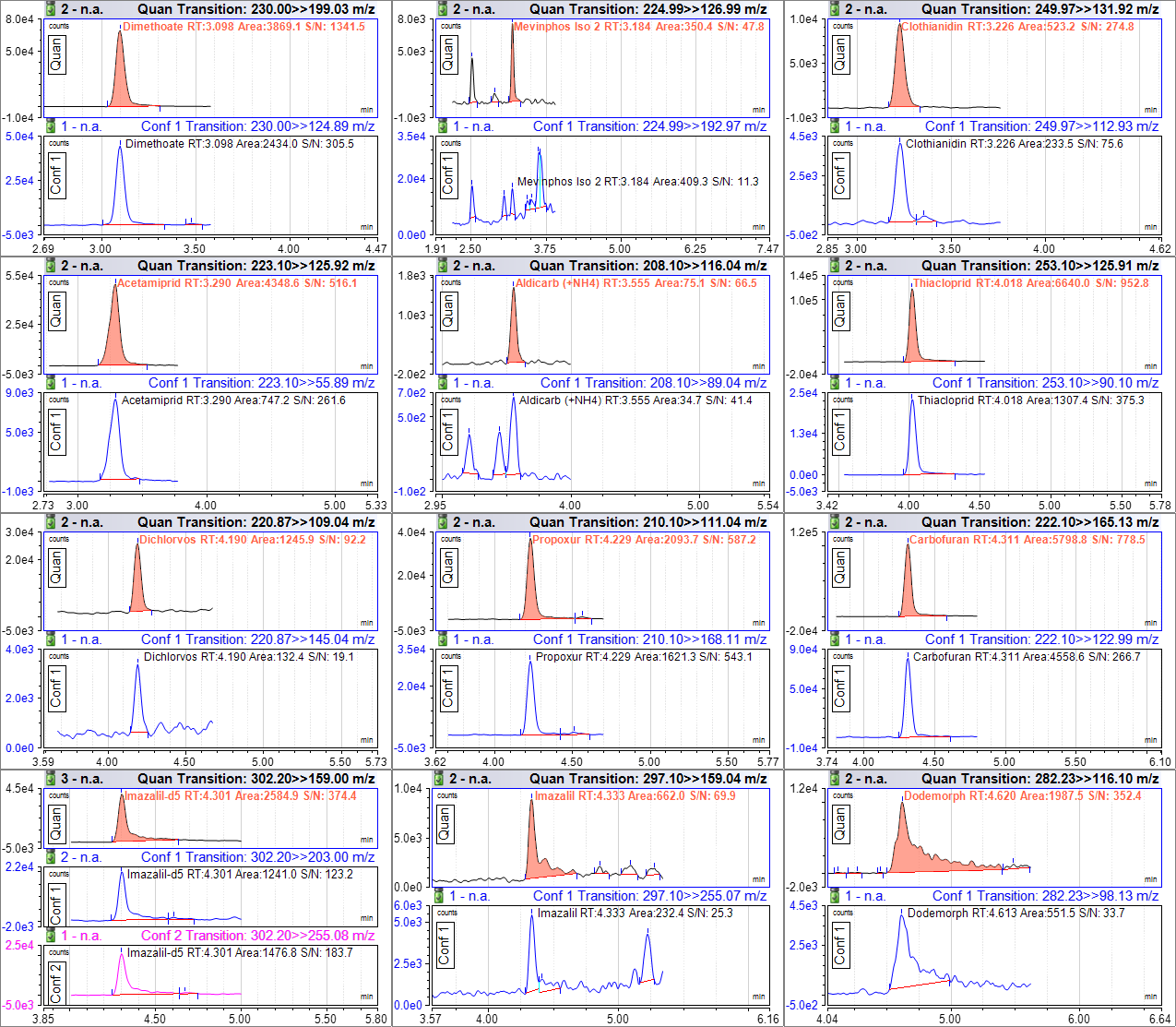


## Figure S 14 - LC-MS/MS Selected Ion Chromatograms at 75 ppb (2 of 9)


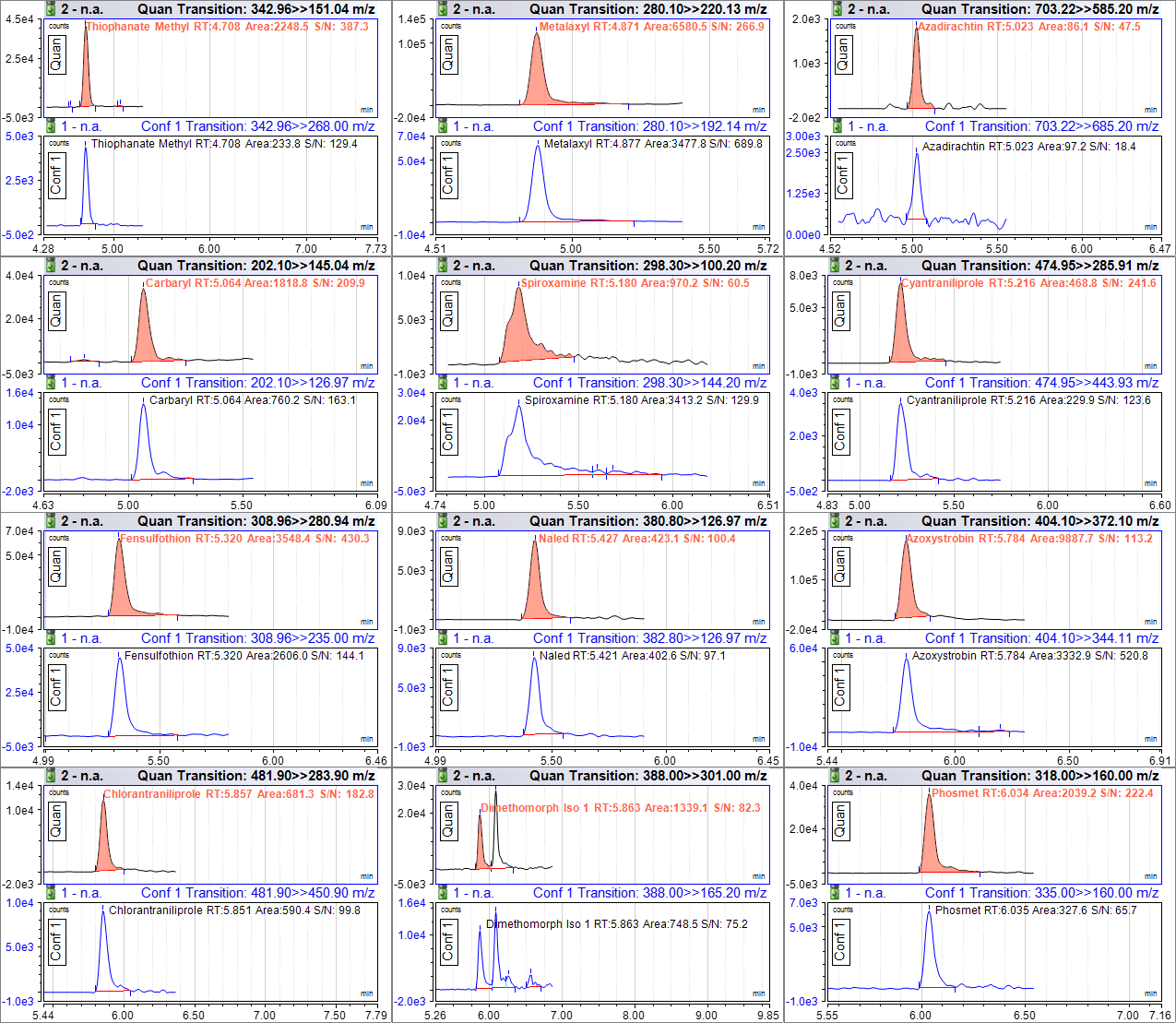


## Figure S 15 - LC-MS/MS Selected Ion Chromatograms at 75 ppb (3 of 9)


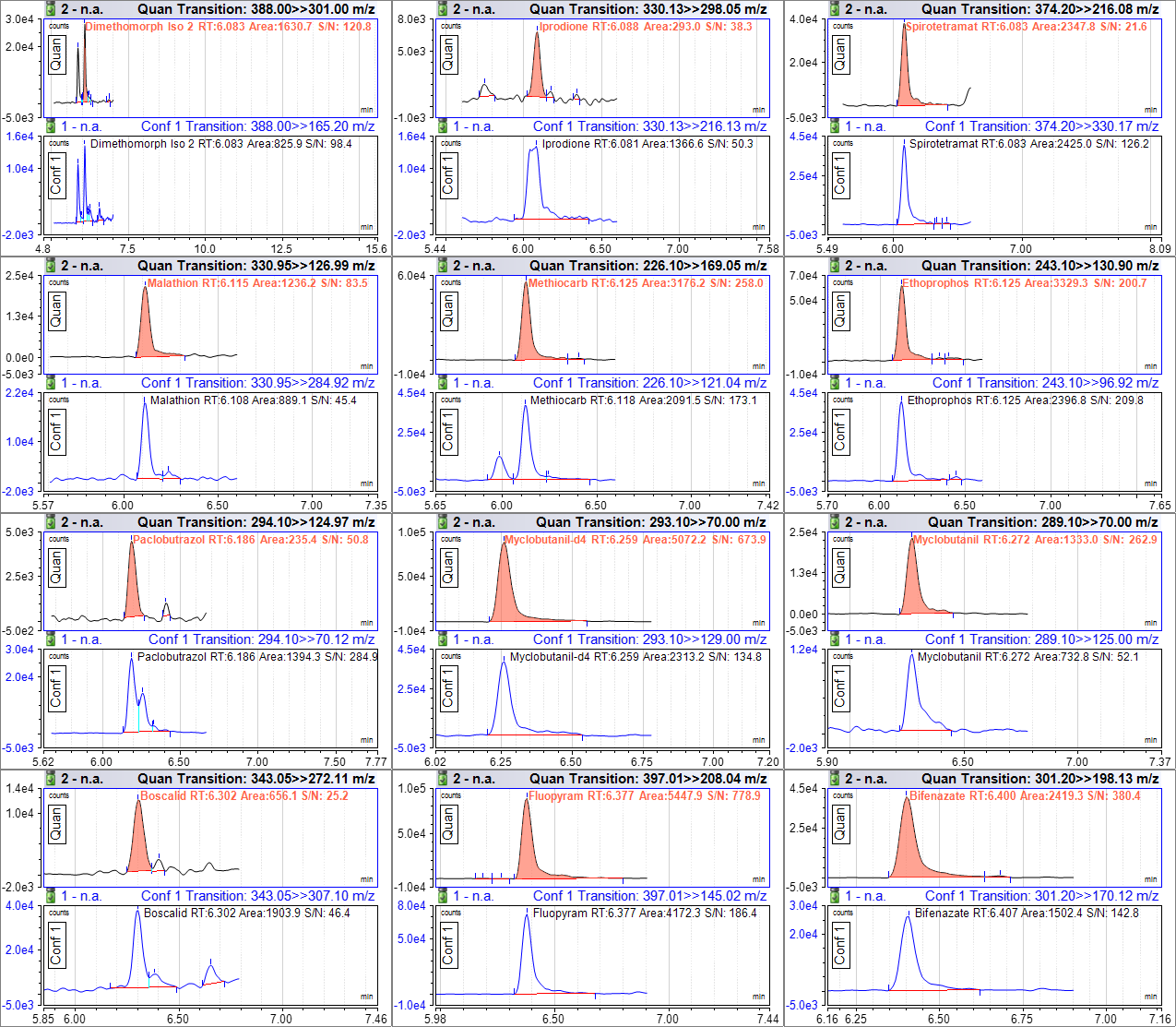


## Figure S 16 - LC-MS/MS Selected Ion Chromatograms at 75 ppb (4 of 9)


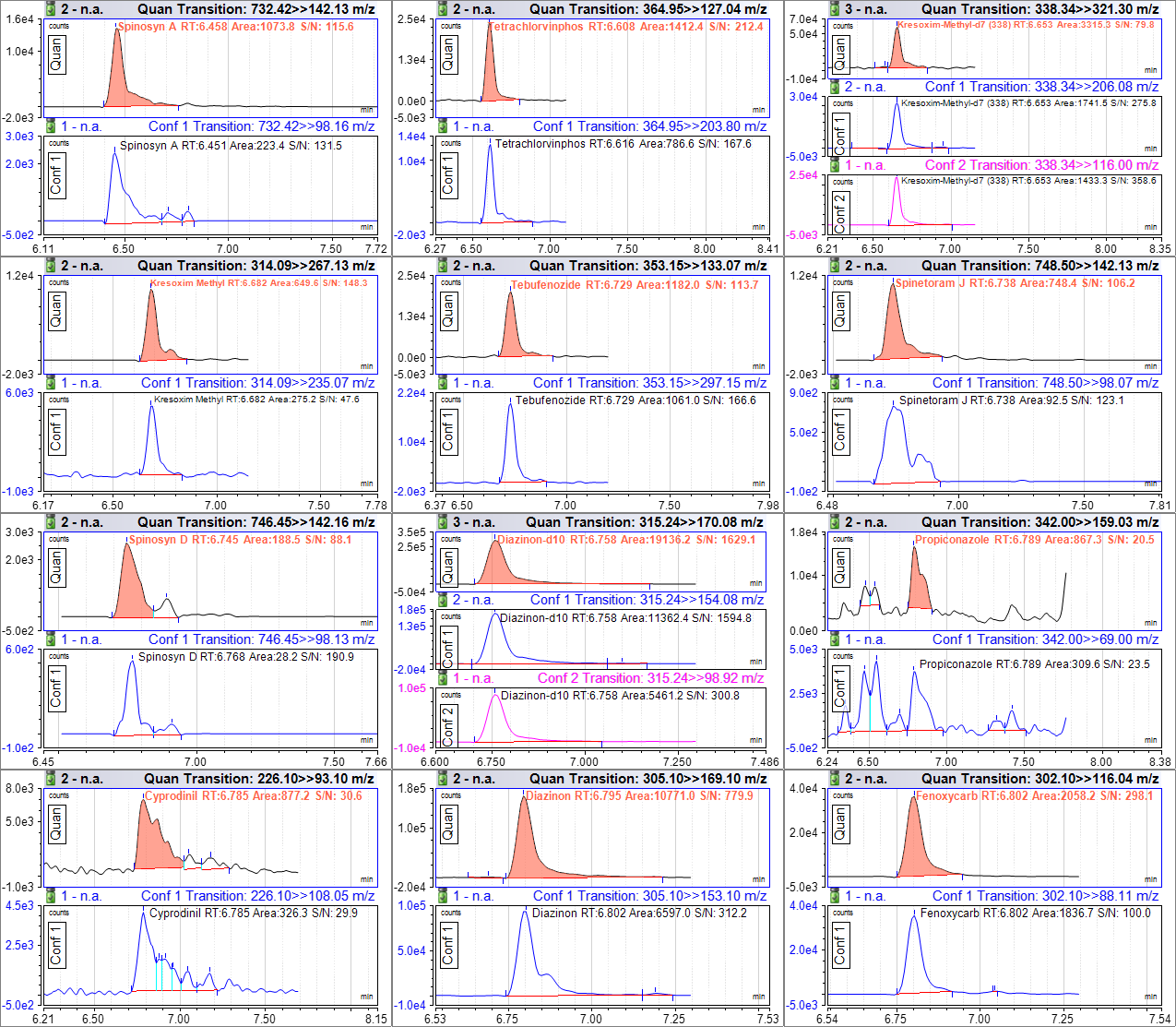


## Figure S 17 - LC-MS/MS Selected Ion Chromatograms at 75 ppb (5 of 9)


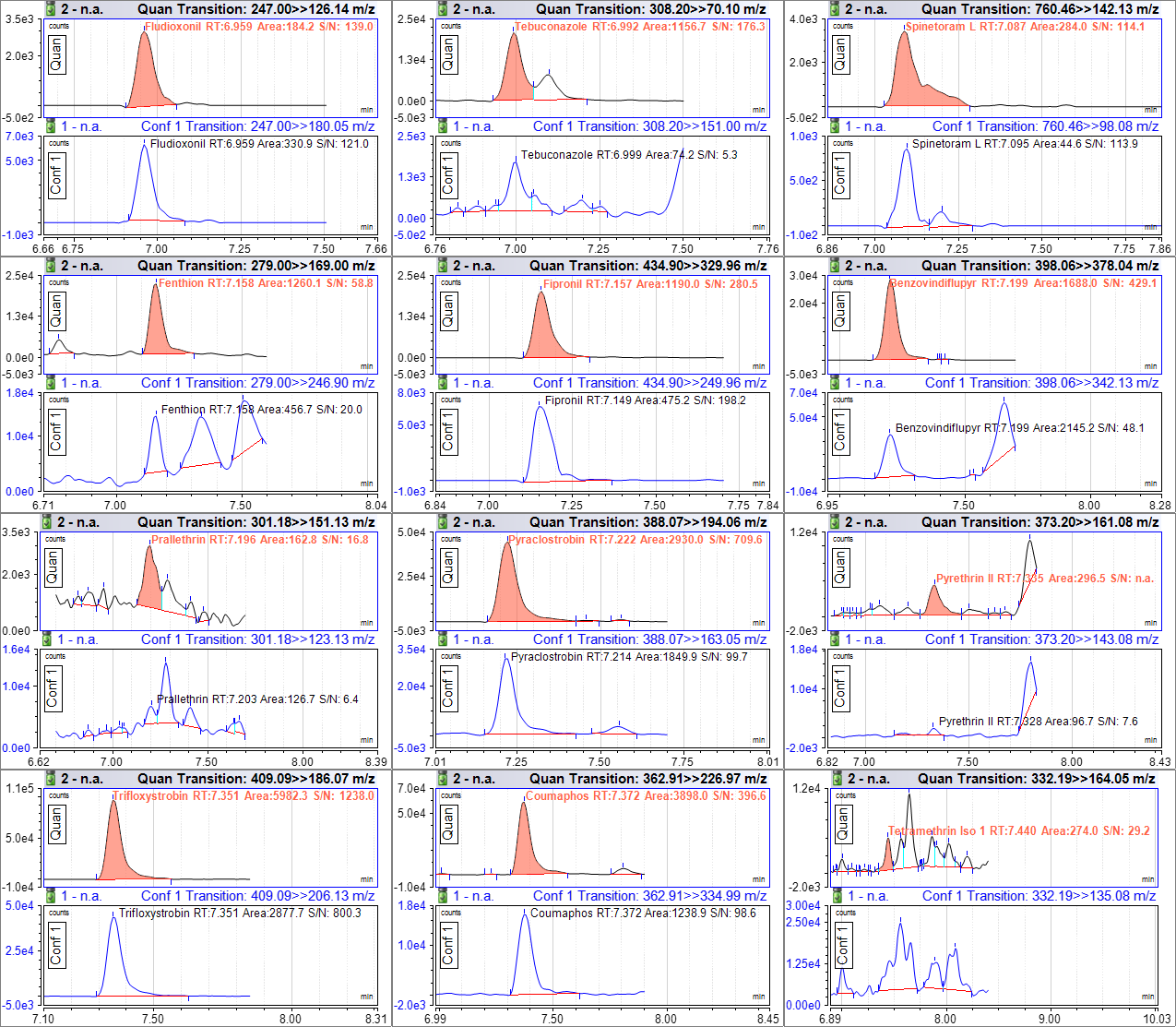


## Figure S 18 - LC-MS/MS Selected Ion Chromatograms at 75 ppb (6 of 9)


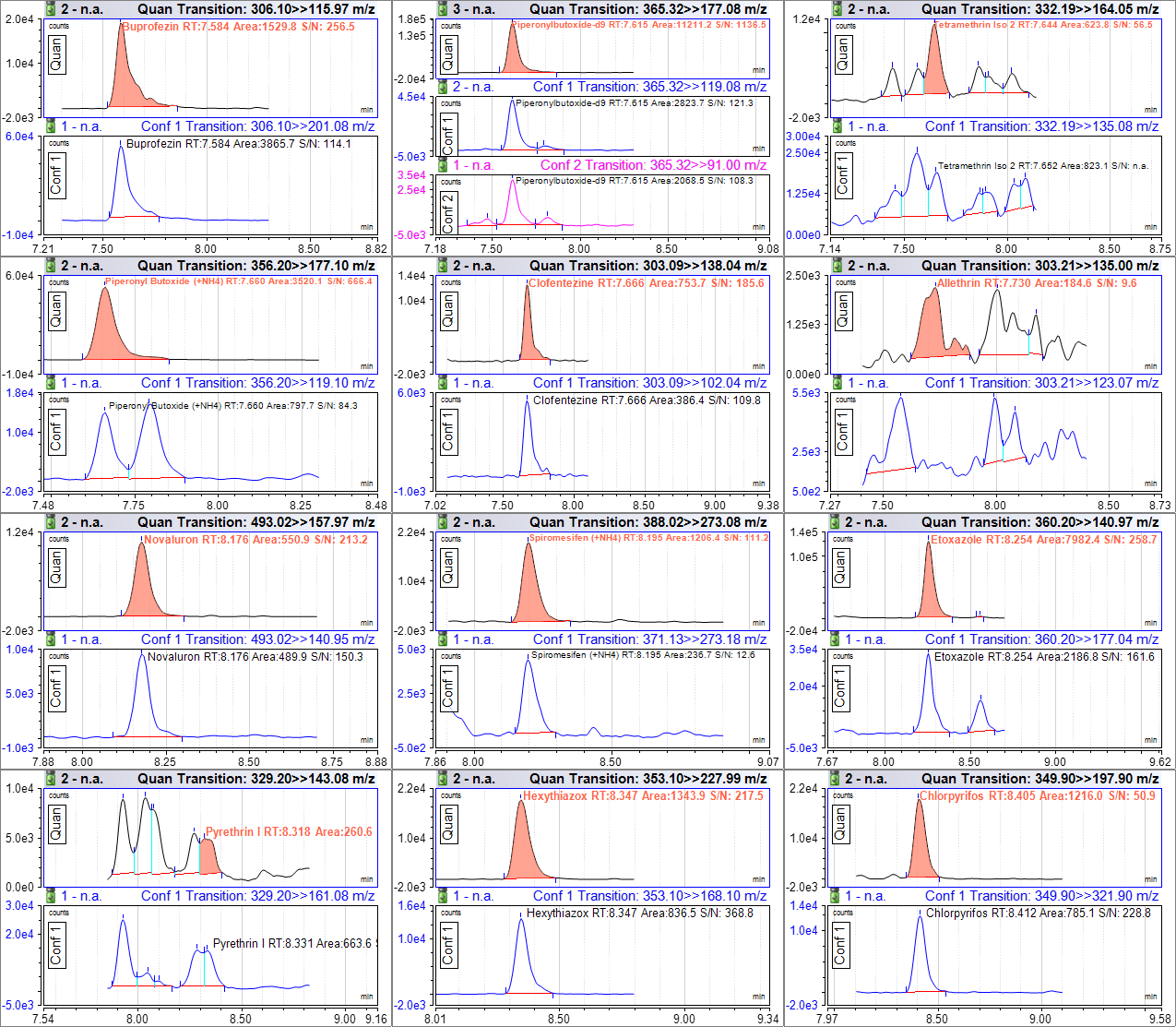


## Figure S 19 - LC-MS/MS Selected Ion Chromatograms at 75 ppb (7 of 9)


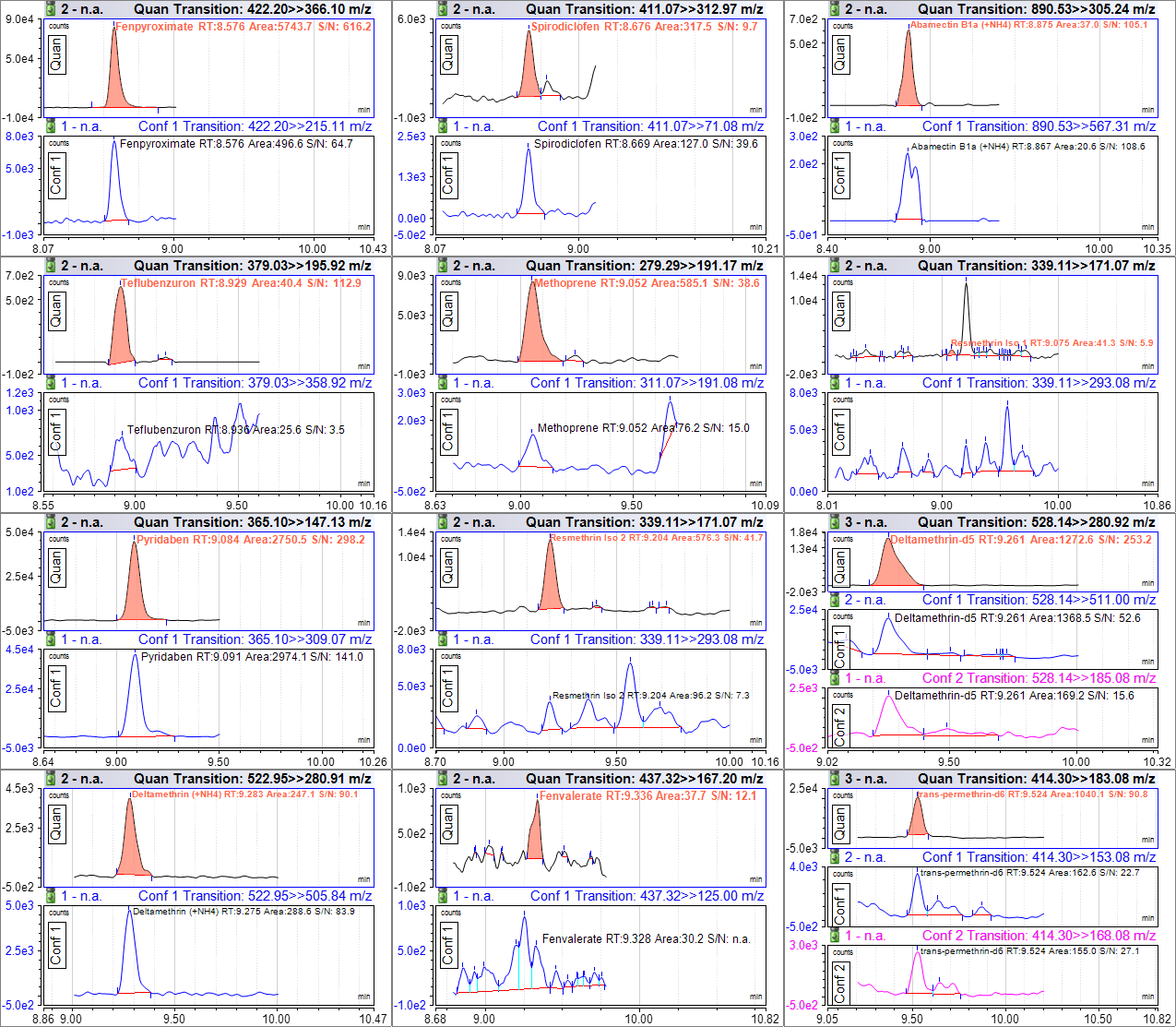


## Figure S 20 - LC-MS/MS Selected Ion Chromatograms at 75 ppb (8 of 9)

## Figure S 21 - LC-MS/MS Selected Ion Chromatograms at 75 ppb (9 of 9)


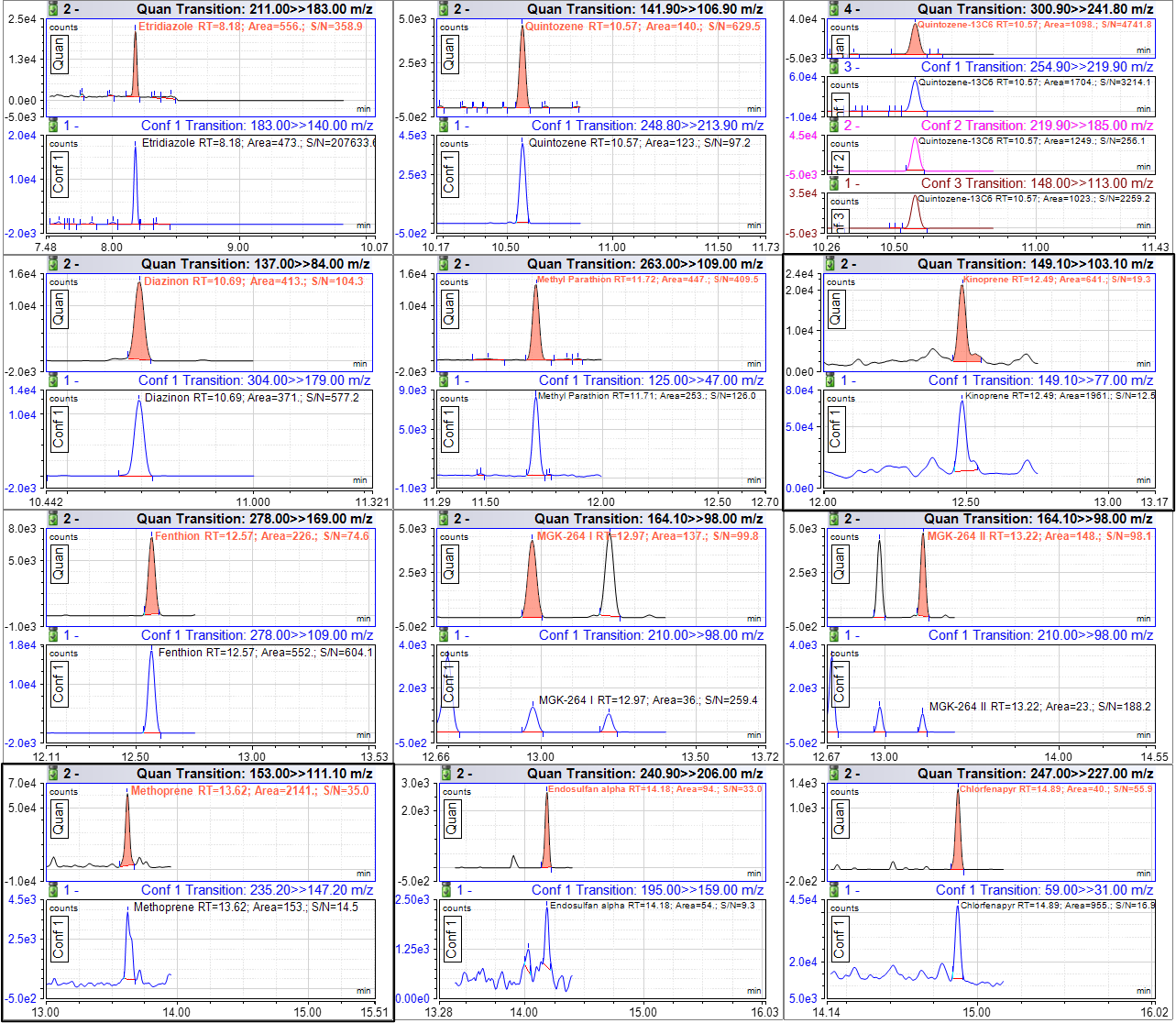


## Figure S 22 - GC-MS/MS Selected Ion Chromatograms at 75 ppb (1 of 3). Note: Kinoprene and Methoprene, highlighted with a bold outline, are displayed at 1250 ppb.


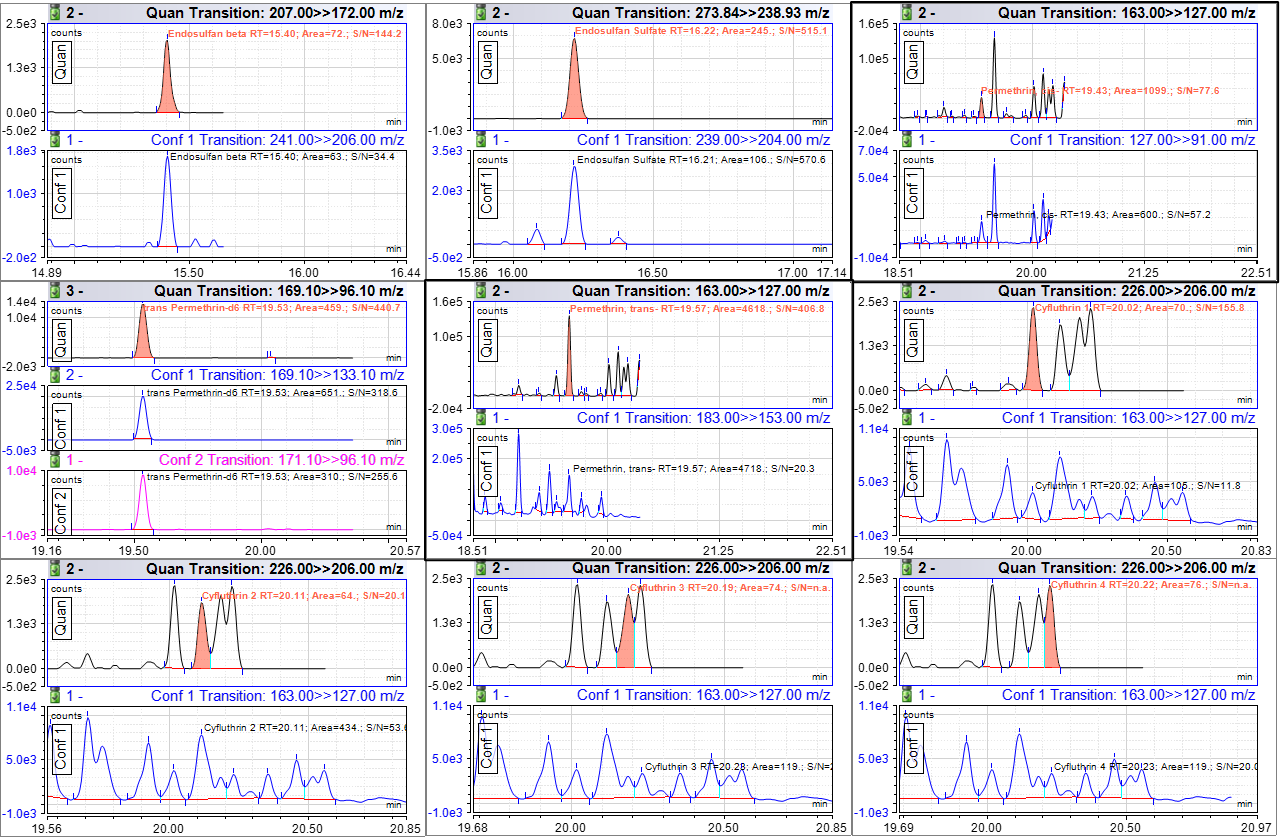


## Figure S 23 - GC-MS/MS Selected Ion Chromatograms at 75 ppb (2 of 3). Note: cis and trans permethrins, highlighted with a bold outline, are displayed at 1250 ppb.


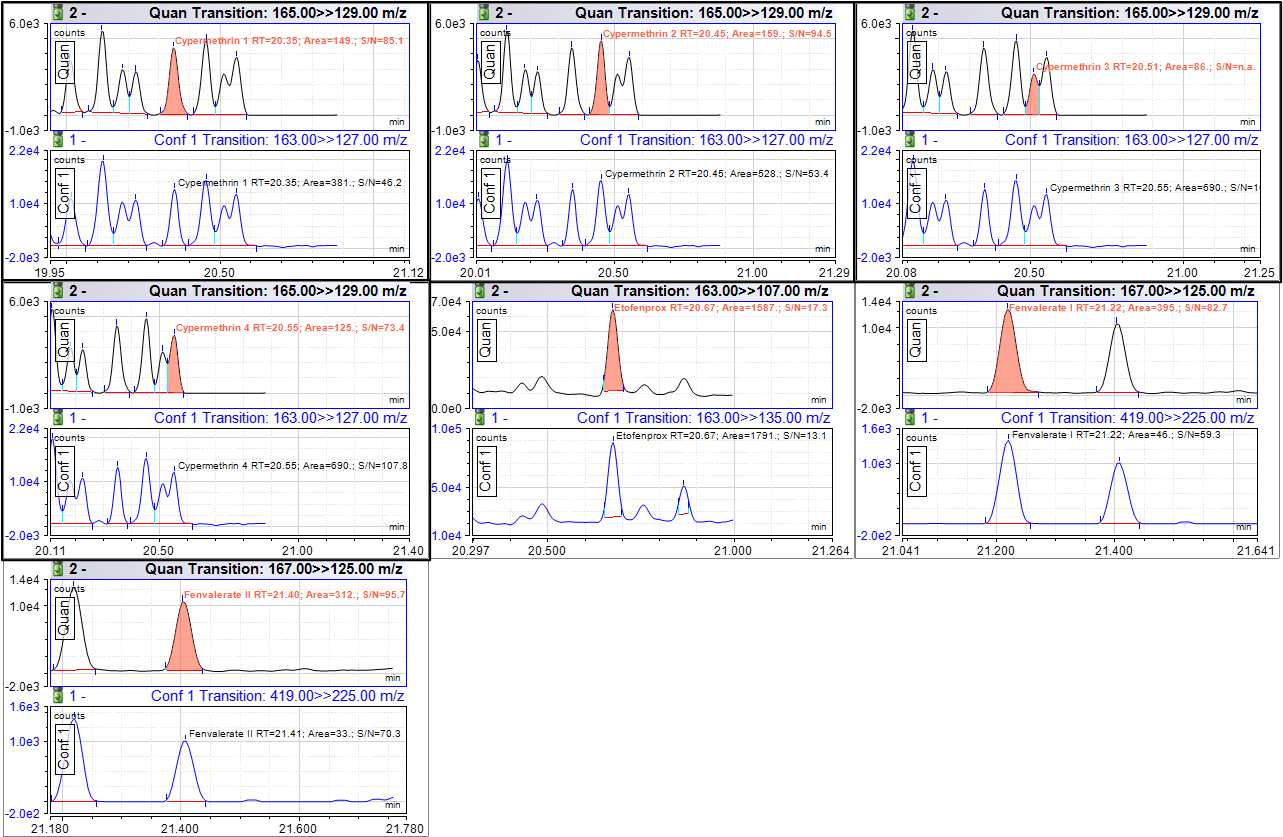


## Figure S 24 - GC-MS/MS Selected Ion Chromatograms at 75 ppb. Note: Cypermethrins 1-4, highlighted with a bold border, are displayed at 250 ppb.


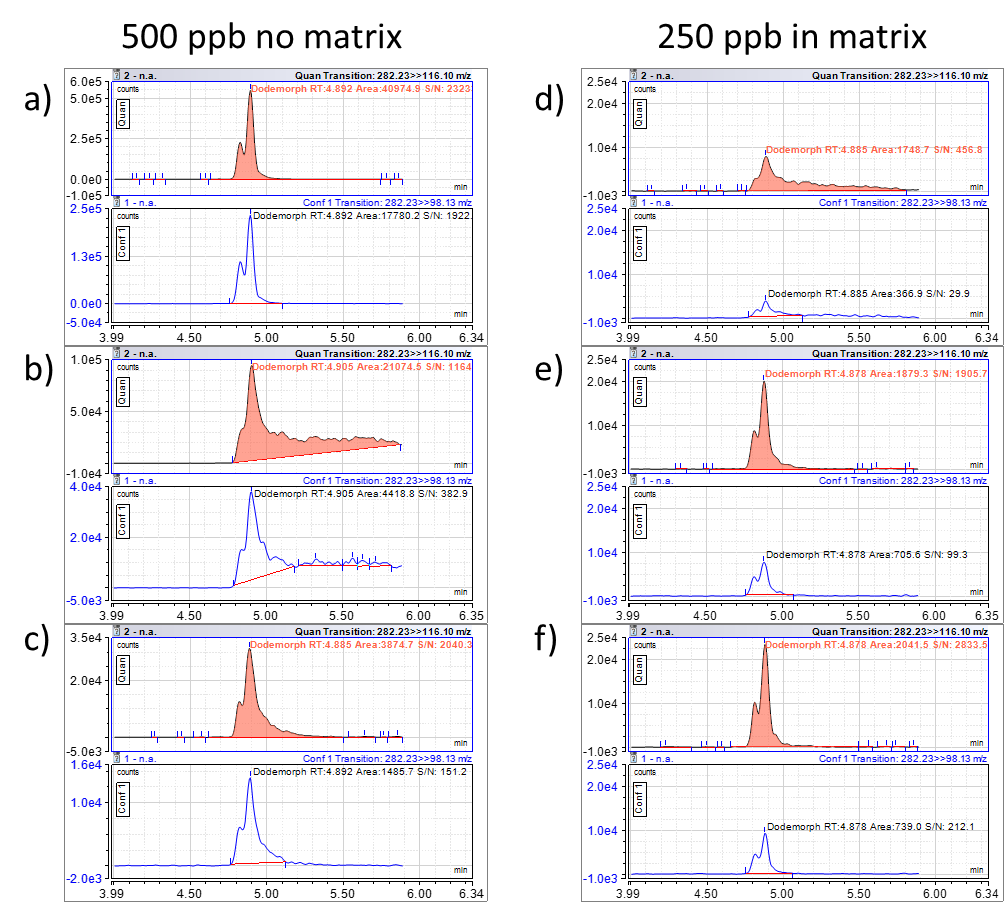


Figure S 25 - Effect of THCA and dilution on peak shape of dodemorph (RT = 4.9 min). a) 500 ppb dodemorph in clean solvent. b) Distorted peak shape of 500 ppb dodemorph in clean solvent with commercial THCA added. c) Partially restored peak shape of 500 ppb dodemorph in clean solvent with commercial THCA added diluted 10x. d) Distorted peak shape of 250 ppb dodemorph extracted from THCA-dominant cannabis cultivar. e) Peak shape of 250 ppb dodemorph extracted from a balanced cannabinoid cultivar. f) Peak shape of 250 ppb dodemorph extracted from a CBDA-balanced cultivar. Similar THCA-dependent peak shape behavior is observed for pesticides spiroxamine (RT = 5.5 min) and cyprodinil (RT = 6.8 min).

75 ppb spiked in matrix

Unspiked matrix

Figure S 26 - LC-MS/MS Chromatogram of Dodemorph, Spiroxamine, and Cyprodinil spiked at 75 ppb on matrix (left) vs unspiked cannabis matrix (right). Monitoring the SRM transitions in the blank matrix shows little to no interfering signals.
